# Multivariate Brain-Blood Signatures in Early-Stage Depression and Psychosis

**DOI:** 10.1001/jamapsychiatry.2025.3803

**Published:** 2025-12-17

**Authors:** David Popovic, Clara Weyer, Dominic B. Dwyer, Sian Lowri Griffiths, Paris Alexandros Lalousis, Nicholas M. Barnes, Clara Vetter, Lisa-Maria Neuner, Madalina-Octavia Buciuman, Elif Sarisik, Marco Paolini, Theresa Lichtenstein, Lana Kambeitz-Ilankovic, Joseph Kambeitz, Stephan Ruhrmann, Katharine Chisholm, Frauke Schultze-Lutter, Peter Falkai, Kolja Schiltz, Johann Steiner, Michael Ziller, Giulio Pergola, Giuseppe Blasi, Alessandro Bertolino, Georg Romer, Rebekka Lencer, Udo Dannlowski, Raimo K. R. Salokangas, Christos Pantelis, Paolo Brambilla, Stefan Borgwardt, Stephen J. Wood, Eva Meisenzahl, Nikolaos Koutsouleris, Rachel Upthegrove, and the PRONIA Consortium

**Affiliations:** 1 Max Planck Institute of Psychiatry, Munich, Germany; 2 Department of Psychiatry and Psychotherapy, Ludwig-Maximilian-University, Munich, Germany; 3 Graduate School of Systemic Neurosciences, Ludwig-Maximilian-University, Munich, Germany; 4 Centre for Youth Mental Health, University of Melbourne, Melbourne, Victoria, Australia; 5 Institute for Mental Health, University of Birmingham, Birmingham, United Kingdom; 6 Institute of Psychiatry, Psychology and Neuroscience, King’s College London, London, United Kingdom; 7 Neuropharmacology Research Group, College of Medicine and Health, University of Birmingham, Birmingham, United Kingdom; 8 International Max Planck Research School for Translational Psychiatry (IMPRS-TP), Munich, Germany; 9 Department of Radiology, LMU University Hospital, Ludwig Maximilian University Munich, Munich, Germany; 10 Department of Psychiatry and Psychotherapy, University of Cologne, Cologne, Germany; 11 Department of Psychology, Faculty of Psychology and Educational Sciences, Ludwig-Maximilian University, Munich, Germany; 12 School of Psychology, University of Sussex, Brighton, United Kingdom; 13 Department of Psychiatry and Psychotherapy, Medical Faculty and University Hospital Düsseldorf, Heinrich-Heine University, Düsseldorf, Germany; 14 Department of Psychology, Faculty of Psychology, Airlangga University, Surabaya, Indonesia; 15 University Hospital of Child and Adolescent Psychiatry and Psychotherapy, University of Bern, Bern, Switzerland; 16 German Center for Mental Health, partner site Munich-Augsburg, Germany; 17 Department of Psychiatry, Otto-von-Guericke-University Magdeburg, Magdeburg, Germany; 18 Center for Intervention and Research on Adaptive and Maladaptive Brain Circuits Underlying Mental Health, Site Jena-Magdeburg-Halle, Magdeburg, Germany; 19 German Center for Mental Health, Halle-Jena-Magdeburg, Germany; 20 Institute for Translational Psychiatry, University of Münster, Münster, Germany; 21 Department of Translational Biomedicine and Neuroscience, University of Bari Aldo Moro, Bari, Italy; 22 Lieber Institute for Brain Development, Johns Hopkins Medical Campus, Baltimore, Maryland; 23 Department of Psychiatry and Behavioral Sciences, Johns Hopkins School of Medicine, Baltimore, Maryland; 24 Department of Child and Adolescent Psychiatry, University of Münster, Münster, Germany; 25 Department of Psychiatry and Psychotherapy, University of Lübeck, Germany; 26 Department of Psychiatry, University of Turku, Turku, Finland; 27 Melbourne Neuropsychiatry Centre, University of Melbourne & Melbourne Health, Melbourne, Victoria, Australia; 28 Department of Neurosciences and Mental Health, Fondazione IRCCS Ca’ Granda Ospedale Maggiore Policlinico, Milano, Italy; 29 Department of Transplantation and Pathophysiology, University of Milan, Milan, Italy; 30 Orygen, the National Centre of Excellence for Youth Mental Health, Melbourne, Victoria, Australia; 31 Department of Psychiatry, Oxford University, Warneford Hospital, Oxford, United Kingdom; 32 Early Intervention Service, Birmingham Women’s and Children’s National Health Service Trust, Birmingham, United Kingdom; 33 National Institute for Health and Care Research, Oxford Health Biomedical Research Centre, Oxford, United Kingdom

## Abstract

**Importance:**

Inflammation is increasingly implicated in the pathophysiology of mood and psychotic disorders. Integrating blood biomarkers and brain imaging may help uncover mechanistic pathways and guide targeted interventions.

**Objective:**

To identify shared and distinct multivariate patterns of peripheral inflammation and gray matter volume (GMV) in early-stage depressive and psychotic disorders using a transdiagnostic machine learning approach.

**Design, Setting, and Participants:**

The naturalistic multicenter PRONIA study was conducted between February 2014 and May 2019 with a follow-up period of up to 36 months; baseline data were analyzed between August 2021 and April 2024. Eight sites, including inpatient and outpatient facilities, in 5 European countries (Germany, Italy, Switzerland, Finland, and the United Kingdom) were included. The study included individuals with recent-onset depression (ROD, n = 163) or psychosis (ROP, n = 177) or clinical high-risk states for psychosis (CHR-P, n = 172), all with minimal medication exposure, and healthy control (HC) individuals (n = 166).

**Exposures:**

Structural magnetic resonance imaging (MRI), peripheral assays of cytokines (eg, interleukin [IL] 6, IL-1β, tumor necrosis factor [TNF] α, C-reactive protein [CRP], brain-derived neurotrophic factor [BDNF], S100 calcium-binding protein B [S100B]); clinical assessments; neurocognitive testing.

**Main Outcomes and Measures:**

After data collection, sparse partial least squares was used to identify latent brain-blood signatures. Support vector machine classification evaluated psychosocial and neurocognitive predictors of signature expression using repeated nested cross-validation.

**Results:**

A total of 678 participants (346 [51.0%] female; median [IQR] age, 24.0 [20.9-28.9] years) were included. Four signatures were identified. A psychosis signature (ρ = 0.27; *P* = .002) differentiated ROP from CHR-P with elevated IL-6, TNF-α, and reduced CRP, alongside GMV shifts in corticothalamic circuits. A depression signature (ρ = 0.19; *P* = .02) differentiated ROD from HC individuals with elevated IL-1β, IL-2, IL-4, S100B, and BDNF and GMV reductions in limbic regions. Additional signatures reflected age (ρ = 0.67) and sex or MRI quality (ρ = 0.53). Psychosocial features, including a differential childhood trauma pattern, predicted both the psychosis (balanced accuracy [BAC] = 67.2%) and depression (BAC = 78.0%) signatures. Cognitive performance predicted only the psychosis signature (BAC = 65.1%).

**Conclusions and Relevance:**

In this study, early-stage depression and psychosis exhibited distinct neurobiological signatures involving immune and neuroanatomical markers, challenging fully dimensional disease models. These signatures are shaped by childhood trauma and cognition and may support biologically informed early interventions.

## Key Points

**Question:**

Can combined peripheral inflammatory markers with whole-brain gray matter volume in a multivariate framework distinguish early-stage depression and psychosis?

**Findings:**

In this cross-sectional study of 678 individuals from the multicenter PRONIA study, sparse partial least squares isolated 2 distinct blood-brain signatures: one differentiating recent-onset psychosis from clinical high-risk states for psychosis and another separating recent-onset depression from healthy control individuals, each with different cytokine and gray matter volume patterns.

**Meaning:**

The findings suggest that peripheral and neuroanatomical biomarkers may have the potential to support stage-specific differentiation and inform stratified approaches to psychiatric care.

## Teaser

This cross-sectional study uses machine learning to evaluate patterns of peripheral inflammation and gray matter volume in individuals with depressive and psychotic disorders.

## Introduction

Depressive and psychotic disorders often emerge in adolescence and early adulthood and contribute significantly to disability worldwide, highlighting the need for early intervention.^1,2^ In response to this need, numerous studies have aimed at identifying modifiable risk factors and biomarkers, and potentially new treatment strategies.^3,4^ There are considerable overlaps between depressive and psychotic disorders,^5,6,7^ including shared etiological mechanisms, such as synaptic dysfunction,^8,9^ genetic risk^10^ or dysfunction of the immune system.^11,12,13^ Among these, immune alterations have gained increasing attention both as potential and readily accessible biomarkers as well as promising therapeutic targets.^11,13^ Dysregulated levels of peripheral inflammatory markers, such as cytokines (eg, interleukins [IL], interferons [IFN], tumor necrosis factors [TNF], transforming growth factors [TGF]) and acute phase proteins (eg, C-reactive protein [CRP]), have been reported for both psychosis and depression, from early clinical high risk for psychosis (CHR-P) states and recent-onset depression (ROD) or psychosis (ROP) to chronic manifestations,^13,14,15,16,17^ at least in a subgroup of affected individuals.^11,12,13^ While certain inflammatory markers, such as IL-6, TNF-α, and CRP, were found to be altered in both disorders, other parameters (eg, IL-1β, IL-8, IL-12, and IL-18) appear to differ,^14,16^ suggesting an intricate pattern of shared and distinct immunological pathways. Notably, prior work has linked peripheral immune alterations to changes in brain structure. In psychosis, higher TNF-α levels have been associated with increased gray matter volume (GMV) of the left thalamus, while higher CRP levels were associated with increased volume of the left putamen.^18^ In depression, higher levels of IL-6 were associated with lower GMV in the hippocampus^19^ and higher levels of CRP with lower GMV in the prefrontal cortex.^20^ Several mechanisms may be involved in the association between immune dysregulation and structural brain alterations, potentially stemming from genetic and environmental risks (eg, substance misuse and stress).^12,21^

To explore whether these brain-inflammation signatures are shared, distinct, or both, comprehensive study designs covering the diagnostic spectrum across affective and psychotic boundaries are needed. Furthermore, inflammatory and brain structural processes may form a higher-order network^22^ of disease pathology characterized by a multitude of subtle, distributed, and complex patterns. This complexity demands sensitive methods that account for individual variation in peripheral inflammation and magnetic resonance imaging (MRI) data.^23,24^ Therefore, multivariate approaches have attracted increasing interest as they offer the potential to integrate multidimensional data and model large-scale complex effect patterns, while also accounting for interdependence and multicollinearity of features.^25,26^

To address this challenge, we designed a study aimed to better understand the impact of inflammatory processes and structural brain alterations in early-stage psychosis and depression. Delineating neurobiologically driven disease trajectories at the earliest possible stage could enhance resource allocation and enable targeted treatment at a vulnerable stage of illness for individuals facing a potentially severe mental disorder for the first time and caretakers facing diagnostic uncertainty. Because both blood-based biomarkers and brain structure are known to be modulated by pharmacological treatment,^27,28^ study samples are required in which disease-related alterations can be clearly isolated from medication-related effects. Consequently, we present a minimally medicated, multicentric, and mixed sample composed of adolescents and young adults aged 15 to 40 years with CHR-P, ROP, or ROD, along with healthy control (HC) individuals. We used the multivariate sparse partial least squares (SPLS) algorithm^29,30^ to find parsimonious data-driven signatures between voxelwise whole-brain GMV and a variety of blood parameters with potential inflammatory properties. In a second step, our aim was to investigate potential clinical embeddings of these inflammation-related brain signatures. To this end, we used support vector machine classification (SVM-C) to predict signature expression using psychosocial measures, medication, and neurocognitive performance, building on prior work linking inflammation to neurocognitive performance and psychosocial measures, such as childhood trauma,^18,31^ and to rule out effects of medication. We hypothesized that early-stage psychosis and depression exhibit both shared and distinct signatures of peripheral inflammation, brain structure, and associated clinical phenotypes. Using this 2-level unsupervised and supervised learning approach followed by a clinical-neurocognitive linkage analysis, we assessed to what extent the emerging neurobiological signatures adhered to established diagnostic boundaries in the earliest stages of the disease.

## Methods

The study followed the Strengthening the Reporting of Observational Studies in Epidemiology (STROBE) reporting guideline (eTable 1 in Supplement 2). Local research ethics committees approved the project according to the principles of the Declaration of Helsinki. All adults provided written informed consent; minors gave written assent, with guardian consent.

### Study Participants

Inpatients and outpatients with ROD, ROP, and CHR-P—according to the ultra–high risk (UHR) criteria^32^ and the basic symptom criterion Cognitive Disturbances^33^—as well as HC individuals were recruited as part of the multisite, prospective, naturalistic European PRONIA study (Personalized Prognostic Tools for Early Psychosis Management, German Clinical Trials Register: DRKS00005042) (eFigure 1 in Supplement 2). Recruitment was carried out between February 2014 and May 2019 at 10 sites in 5 European countries (Germany, Italy, Switzerland, Finland, and the United Kingdom) (eTables 2-4 in Supplement 2). Inclusion criteria allowed only for minimal antipsychotic medication. Baseline data from 678 individuals (163 with ROD, 177 with ROP, 172 with CHR-P, and 166 HC)—with 453 individuals from the discovery sample (100 with ROD, 95 with ROP, 93 with CHR-P, and 165 HC) and 225 individuals from the replication sample (63 with ROD, 82 with ROP, 79 with CHR-P, and 1 HC)—acquired from 8 sites (Munich, Cologne, Muenster, Basel, Milan, Udine, Birmingham, and Turku), where both structural MRI and blood data were available (≤20% missing), were used (Table; eMethods, eTables 2-7, and eFigure 2 in Supplement 2).

**Table.** Demographic and Clinical Characteristics of the Study Sample

Characteristic	Median (IQR)					χ^2^/*z*^a^	η^2^/*V*^b^	*P* value^c^
	All	CHR-P	HC	ROD	ROP			
No.	678	172	166	163	177	NA	NA	NA
Sociodemographic data								
Age, y	24.0 (20.9 to 28.9)	22.2 (19.9 to 26.5)	25.2 (21.4 to 30.4)	23.7 (20.7 to 29.3)	24.6 (21.3 to 30.9)	14.97	0.03	.03^d^
Sex, No. (%)								
Female	346 (51.0)	92 (53.5)	99 (59.6)	77 (47.2)	78 (44.1)			
Male	332 (49.0)	80 (46.5)	67 (40.4)	86 (52.8)	99 (55.9)	3.20	0.08	>.99
BMI	22.5 (20.4 to 25.1)	22.5 (20.2 to 25.3)	22.1 (20.2 to 24.4)	23.6 (20.3 to 25.3)	22.5 (20.8 to 25.2)	1.20	.00	>.99
Right-handedness, No. (%)	544 (80.0)	134 (77.9)	149 (89.8)	127 (77.9)	134 (75.7)	2.13	0.07	>.99
Education, y	14 (12 to 17)	13 (12 to 16)	16 (14 to 18)	14 (13 to 17)	13 (12 to 16)	6.55	.00	>.99
Tobacco use, No. (%)	257 (38.0)	72 (41.9)	35 (21.1)	68 (41.7)	82 (46.3)	1.02	0.05	>.99
Symptomatology								
BDI-II	18.0 (5.0 to 30.0)	28.0 (19.8 to 35.0)	2.0 (0.0 to 5.0)	26.5 (16.0 to 34.0)	19.0 (11.0 to 29.5)	27.77	0.06	<.001^d^
PANSS								
Total score	55.0 (45.0 to 69.0)	52.0 (44.0 to 65.5)	NA	47.0 (41.0 to 57.0)	68.0 (55.5 to 81.5)	106.19	0.21	<.001^d^
Positive symptoms	11 (8 to 16)	11 (9 to 14)	NA	8 (7 to 9)	19 (15 to 23)	296.41	0.60	<.001^d^
Negative symptoms	13 (9 to 18)	12 (9 to 18)	NA	12 (9 to 17)	15 (10 to 21)	13.66	0.02	.06
General symptoms	29 (24 to 36)	28 (24 to 35)	NA	27 (23 to 32)	33 (27 to 41)	37.88	0.07	<.001^d^
Blood parameters								
IFN-γ, pg/mL	1.59 (1.14 to 3.33)	1.59 (1.14 to 2.68)	1.59 (1.16 to 3.33)	1.59 (1.14 to 3.33)	1.59 (1.14 to 3.33)	0.75	.00	>.99
IL-1RA, pg/mL	480.51 (366.94 to 657.19)	481.28 (375.01 to 671.34)	455.96 (324.31 to 618.23)	522.82 (384.60 to 693.21)	476.80 (366.85 to 637.74)	4.06	.00	>.99
IL-4, pg/mL	8.01 (3.10 to 11.73)	8.01 (3.83 to 13.59)	8.03 (3.10 to 8.36)	8.01 (3.10 to 11.73)	8.01 (3.10 to 9.203)	1.60	.00	>.99
S100B, pg/mL	37.51 (12.10 to 78.90)	41.85 (14.37 to 85.09)	26.40 (12.10 to 55.38)	42.54 (12.10 to 86.92)	42.54 (12.10 to 86.92)	0.15	.00	>.99
IL-1β, pg/mL	0.57 (0.48 to 1.46)	0.73 (0.48 to 1.93)	0.57 (0.10 to 0.99)	0.57 (0.48 to 1.46)	0.73 (0.48 to 1.46)	2.63	.00	>.99
IL-2, pg/mL	0.72 (0.30 to 1.43)	0.74 (0.43 to 1.43)	0.43 (0.27 to 0.97)	0.74 (0.43 to 1.43)	0.74 (0.44 to 2.04)	0.95	.00	>.99
IL-6, pg/mL	0.53 (0.22 to 1.03)	0.64 (0.22 to 1.11)	0.44 (0.25 to 0.85)	0.59 (0.14 to 1.03)	0.49 (0.22 to 1.11)	0.30	.00	>.99
TNF-α, pg/mL	1.52 (0.71 to 2.43)	1.52 (0.75 to 2.55)	1.34 (0.71 to 2.05)	1.66 (0.66 to 2.60)	1.72 (0.75 to 2.55)	0.21	.00	>.99
CRP, mg/L	0.58 (0.22 to 1.46)	0.58 (0.25 to 1.39)	0.48 (0.18 to 1.46)	0.61 (0.19 to 1.60)	0.57 (0.24 to 1.39)	0.29	.00	>.99
TGF-β, ng/mL	149.6 (122.0 to 226.4)	149.6 (117.7 to 204.9)	147.7 (112.5 to 585.3)	158.2 (127.6 to 210.8)	146.5 (124.1 to 203.7)	2.88	.00	>.99
BDNF, ng/mL	22.73 (18.06 to 27.27)	22.96 (18.08 to 27.56)	22.36 (17.77 to 27.18)	23.15 (18.74 to 26.77)	22.50 (17.58 to 27.87)	0.60	.00	>.99
Length of storage, d	1385.0 (1062.0 to 1632.0)	1258.0 (832.8 to 1555.5)	1608.0 (1461.0 to 1699.8)	1321.0 (1045.5 to 1579.5)	1235.0 (885.0 to 1529.8)	2.19	.00	>.99
Level of functioning								
GAF:S, past mo	55 (45 to 75)	51 (45 to 60)	85 (85 to 91)	53 (49 to 60)	40 (31 to 51)	79.43	0.15	<.001^d^
GAF:D/I, past mo	55 (42 to 80)	51 (42 to 60)	85 (81 to 88)	51 (45 to 61)	40 (35 to 51)	59.36	0.11	<.001^d^
GF:S, current	7 (6 to 8)	6 (5 to 7)	9 (8 to 9)	6 (6 to 7)	6 (5 to 7)	21.14	0.04	.001^d^
GF:R, current	7 (5 to 8)	6 (5 to 7)	8 (8 to 9)	6 (5 to 7)	5 (4 to 6)	22.89	0.04	<.001^d^
NEO-FFI								
Neuroticism	38 (30 to 46)	44 (38 to 49)	27 (22 to 31)	43 (38 to 49)	39 (32 to 44)	27.32	0.06	<.001^d^
Extraversion	36 (31 to 43)	31 (27 to 36)	44 (40 to 47)	34 (30 to 39)	36 (31 to 42)	24.00	0.05	<.001^d^
Openness	41 (37 to 45)	41 (36 to 45)	42 (38 to 45)	39 (36 to 43)	41 (37 to 45)	7.42	0.01	>.99
Agreeableness	43 (38 to 48)	40 (36 to 46)	47 (43 to 51)	44 (39 to 47)	42 (37 to 46)	16.06	0.03	.02^d^
Conscientiousness	41 (36 to 46)	37 (32 to 42)	47 (42 to 51)	39 (35 to 44)	40 (35 to 46)	14.58	0.03	.04^d^
WHOQOL-BREF								
Physical	25 (21 to 30)	22 (19 to 25)	31 (30 to 33)	22 (19 to 26)	25 (21 to 28)	22.02	0.04	<.001^d^
Psychosocial	18 (14 to 23)	15 (12 to 18)	24 (23 to 26)	15 (12 to 19)	18 (14 to 22)	41.51	0.09	<.001^d^
Social relationships	10 (8 to 12)	9 (7 to 11)	12 (11 to 13)	9 (7 to 11)	10 (7 to 11)	1.81	.00	>.99
Environment	30 (27 to 34)	29 (25 to 31)	34 (31 to 36)	29 (26 to 32)	29 (25 to 33)	1.28	.00	>.99
CTQ								
Emotional abuse	8 (6 to 11)	9 (7 to 14)	6 (5 to 7)	8 (6 to 11)	9 (6 to 11)	7.36	.00	>.99
Physical abuse	5 (5 to 6)	5 (5 to 6)	5 (5 to 5)	5 (5 to 7)	5 (5 to 7)	0.67	.00	>.99
Sexual abuse	5 (5 to 5)	5 (5 to 5)	5 (5 to 5)	5 (5 to 5)	5 (5 to 5)	2.50	.00	>.99
Emotional neglect	10 (7 to 14)	11 (9 to 16)	7 (5 to 10)	10 (8 to 14)	10 (7 to 14)	2.76	.00	>.99
Physical neglect	6 (5 to 8)	7 (5 to 9)	5 (5 to 6)	6 (5 to 9)	7 (5 to 9)	2.44	.00	>.99
MATRICS battery								
Social cognition	−0.01 (−0.42 to 0.80)	−0.01 (−0.42 to 0.80)	0.40 (−0.01 to 0.80)	0.40 (−0.42 to 0.80)	−0.01 (−0.93 to 0.40)	13.28	0.02	.07
Working memory	−0.09 (−0.60 to 0.67)	0.16 (−0.60 to 0.67)	0.41 (−0.35 to 1.17)	−0.09 (−0.85 to 0.67)	−0.35 (−0.85 to 0.16)	20.55	0.04	.002^d^
Speed of processing	0.05 (−0.31 to 0.37)	−0.03 (−0.35 to 0.29)	0.295 (−0.01 to 0.67)	0.14 (−0.19 to 0.42)	−0.29 (−0.77 to 0.06)	51.66	0.10	<.001^d^
Verbal learning	0.14 (−0.55 to 0.71)	0.14 (−0.55 to 0.53)	0.483 (−0.09 to 0.94)	0.14 (−0.32 to 0.71)	−0.32 (−1.10 to 0.37)	19.83	0.04	.003^d^
Reasoning	0.25 (−0.51 to 0.76)	0.25 (−0.25 to 0.76)	0.51 (−0.25 to 0.82)	0.25 (−0.51 to 0.76)	−0.25 (−1.27 to 0.51)	19.25	0.10	.004^d^
Attention	0.20 (−0.99 to 1.30)	0.08 (−1.235 to 1.19)	0.83 (−0.38 to 1.63)	0.25 (−0.71 to 1.44)	−0.30 (−1.63 to 0.59)	12.93	0.02	.08
Global score	0.54 (−2.24 to 2.97)	0.34 (−2.27 to 2.73)	2.25 (0.17 to 4.63)	0.73 (−1.59 to 3.33)	−1.68 (−4.93 to 1.05)	36.29	0.08	<.001^d^
Medication								
Chlorpromazine-Eq, mg	0 (0 to 1170)	0 (0 to 637.5)	0 (0 to 0)	0 (0 to 0)	3840 (920 to 12375)	168.99	0.42	<.001^d^
Olanzapine-Eq, mg	0 (0 to 42.81)	0 (0 to 26.81)	0 (0 to 0)	0 (0 to 0)	131.33 (30.7 to 412.5)	167.35	0.42	<.001^d^
SSRI-Eq, mg	0 (0 to 730.96)	622.22 (0 to 3340.25)	0 (0 to 0)	466.67 (10.34 to 2438.70)	0 (0 to 331.50)	42.17	0.10	<.001^d^
Diazepam-Eq, mg	0 (0 to 0)	0 (0 to 6.50)	0 (0 to 0)	0 (0 to 42.50)	0 (0 to 182.75)	11.52	0.02	.17
Additional information								
Image quality rating	1.88 (1.82 to 1.97)	1.88 (1.82 to 1.95)	1.89 (1.82 to 2.01)	1.86 (1.82 to 1.98)	1.88 (1.82 to 1.96)	0.370	.00	>.99
Site	NA	NA	NA	NA	NA	34.48	.00	.26

Abbreviations: BDI-II, Beck Depression Inventory II; BDNF, brain-derived neurotrophic factor; BMI, body mass index (calculated as weight in kilograms divided by height in meters squared); CHR-P, clinical high risk for psychosis; CRP, C-reactive protein; CTQ, Childhood Trauma Questionnaire; Eq, equivalents; GAF, Global Assessment of Functioning; GF, global functioning; IFN, interferon; IL, interleukin; NA, not applicable; NEO-FFI, NEO Five-Factor Inventory; PANSS, Positive and Negative Syndrome Scale; ROP, recent-onset psychosis; ROD, recent-onset depression; S100B, S100 calcium-binding protein B; SSRI, selective serotonin reuptake inhibitor; TGF, transforming growth factor; TNF, tumor necrosis factor; WHOQOL-BREF, World Health Organization Quality of Life–Brief Version.

^a^ Group-level differences were assessed using the Kruskal-Wallis test for continuous variables and the χ^2^ test for categorical variables (eTables 6 and 7 in Supplement 2).

^b^ Effect sizes were estimated using η^2^ for continuous variables and Cramér *V* for categorical variables.

^c^ *P* values were adjusted for multiple testing using Bonferroni correction, the entire table representing a family of tests.

^d^ Significant group-level differences between the diagnostic subgroups (excluding healthy control individuals) were found for age; BDI; PANSS total; positive and general subscale scores; GAF:S; GAF:D/I; GF:S; and GF:R within the past month; the NEO-FFI personality domains neuroticism, extraversion, agreeableness, and conscientiousness; the quality of life domains physical and psychosocial; and the neurocognitive domains working memory, processing speed, verbal learning, reasoning, and the global score. Antipsychotic and antidepressant treatment also differed significantly between subgroups.

### Peripheral Blood Parameter Assays

Serum concentrations were determined with the Luminex platform (Bio-Plex 200 system with Bio-Plex Manager software [Bio-Techne]) using a commercial multiplex kit for IFN-γ, IL-1β, IL-1RA, IL-2, IL-4, IL-6, S100B, and TNF-α, and using commercial singleplex kits for high-sensitivity CRP, BDNF, and TGF-β following the manufacturer’s instructions. Assays were blinded to case status and analyzed at the Birmingham Barnes Laboratory (eMethods in Supplement 2).

### MRI Data Acquisition and Visualization

T1-weighted structural MRI scans were resliced to 3 mm and preprocessed using the CAT12 toolbox version 1207, an extension of the SPM12 software version 6685 (Wellcome Department of Cognitive Neurology). Using SPM12, we created a sample-specific gray matter mask; GMV was adjusted for total intracranial volume. Overall image quality was evaluated using the CAT12 image quality rating, a composite score of noise and spatial resolution (eMethods and eFigure 3 in Supplement 2). SPLS voxel weights were mapped to anatomical brain regions and large-scale brain networks using the Brainnetome,^34^ Diedrichsen,^35^ and the Yeo^36^ and Buckner^37^ atlases, respectively.

### Statistical Analysis

#### SPLS

We used SPLS using the toolbox by Popovic et al^29^ to identify multivariate associations between 2 data domains: (1) a blood-based matrix (20 features) including 11 blood parameters (IFN-γ, IL-1β, IL-1RA, IL-2, IL-4, IL-6, S100B, TNF-α, CRP, BDNF, and TGF-β), and associated factors (age, sex, body mass index [BMI], study group, and image quality rating) and (2) a brain matrix (47 888 features) containing vectorized voxelwise whole-brain GMV. The SPLS algorithm uses singular value decomposition to generate latent variables (LVs), each consisting of weight vectors assigning weights to each feature in the respective data matrix. These weights indicate the direction and strength of the covariance between the respective features. For each LV, individual latent scores are calculated by multiplying the blood and brain data of each participant by the corresponding weight vectors, resulting in individualized loadings on these weight vectors. The correlation between these latent scores, reflecting the strength of covariance captured by the LV, served as the optimization criterion. We embedded the SPLS algorithm within a nested cross-validation framework^38^ (eFigure 4 in Supplement 2) with group stratification, adjusting the GMV data for MR scanner effects^39^ and assessing significance through permutation testing. Feature stability was evaluated using bootstrap ratios,^40^ and significant LVs were iteratively removed using projection deflation^30^ (eMethods in Supplement 2).

#### Exploration of SPLS Signatures Using SVM-C

We grouped individuals into those with high scores (ie, both blood parameter and brain latent scores in the top quartile of the latent score distribution) and low scores (ie, both latent scores in the bottom quartile of the latent score distribution) on the LVs of interest. Then, we used SVM-C to predict those with high vs low scores based on 3 baseline predictor domains: (1) psychosocial (43 features: Childhood Trauma Questionnaire [CTQ],^41^ Global Assessment of Functioning [GAF],^42^ Global Functioning [GF],^43^ Neuroticism, Extraversion, Openness Five Factor Inventory [NEO-FFI],^44^ Premorbid Adjustment Scale [PAS],^45^ WHO Quality of Life–Brief Version [WHOQOL-BREF])^46^; (2) neurocognition (7 features: Measurement and Treatment Research to Improve Cognition in Schizophrenia [MATRICS] domains^47^) (eTable 8 in Supplement 2); and (3) medication (4 features: dosage equivalents of chlorpromazine, olanzapine, selective serotonin reuptake inhibitors, and diazepam). SVM-C was used to jointly model integrated brain-blood signatures. We used a linear-kernel SVM, a repeated nested CV framework with 10 folds and 10 permutations each at the CV_1_ and CV_2_ level, using the NeuroMiner platform version 1.1. Balanced accuracy (BAC), the mean of sensitivity and specificity, served as the optimization criterion.^38^ Model significance was tested using 5000 label permutations. Significance and stability of the predictive features was assessed by means of sign-based consistency^48^ (eMethods in Supplement 2).

## Results

### SPLS Results

**Figure 1.**
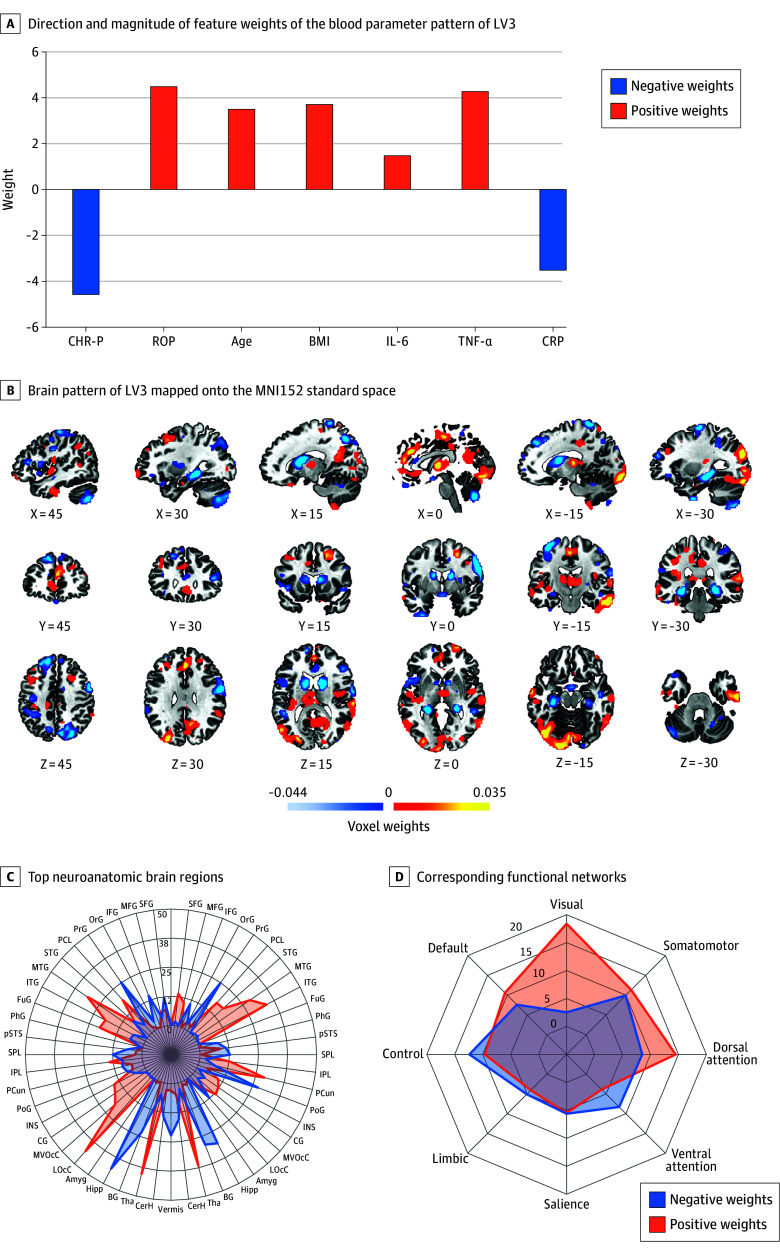
Psychosis Signature A, The bar plot displays both direction and magnitude of feature weights of the blood parameter pattern of latent variable 3 (LV3). If 2 feature weights had the same sign (ie, both positive or both negative), the respective features covaried positively with each other; an opposite direction of feature weights represents a negative covariation. Zero weights indicate that there was no significant contribution of the respective features to the covariance signature. Positive weights were assigned to recent-onset psychosis (ROP) status, age, body mass index (BMI), and levels of interleukin 6 (IL-6), tumor necrosis factor α (TNF-α), clinical high risk for psychosis (CHR-P), and C-reactive protein (CRP) status were negatively weighted. B, The brain pattern of LV3 was mapped onto the MNI152 standard space via the open-source 3-dimensional rendering software Connectome Workbench version 1.4.2. The spider plots highlight the top neuroanatomic brain regions (C; derived from the Brainnetome^34^ and Diedrichsen^35^ atlases) and corresponding functional networks (D; derived from an adapted, 8-network solution of the Yeo^36^ and Buckner^37^ atlases) according to the percentage of positive and negative voxels in these regions. Amyg indicates amygdala; BG, basal ganglia; CerH, cerebellum hemisphere; CG, cingulate gyrus; FuG, fusiform gyrus; Hipp, hippocampus; IFG, inferior frontal gyrus; INS, insular gyrus; IPL, inferior parietal lobule; ITG, inferior temporal gyrus; LOcC, lateral occipital cortex; MFG, middle frontal gyrus; MTG, middle temporal gyrus; MVOcC, medioventral occipital cortex; OrG, orbital gyrus; PCun, precuneus; PCL, paracentral lobule; PhG, parahippocampal gyrus; PoG, postcentral gyrus; PrG, precentral gyrus; pSTS, posterior superior temporal sulcus; SFG, superior frontal gyrus; SPL, superior parietal lobule; STG, superior temporal gyrus; Tha, thalamus; TNF, tumor necrosis factor.

**Figure 2.**
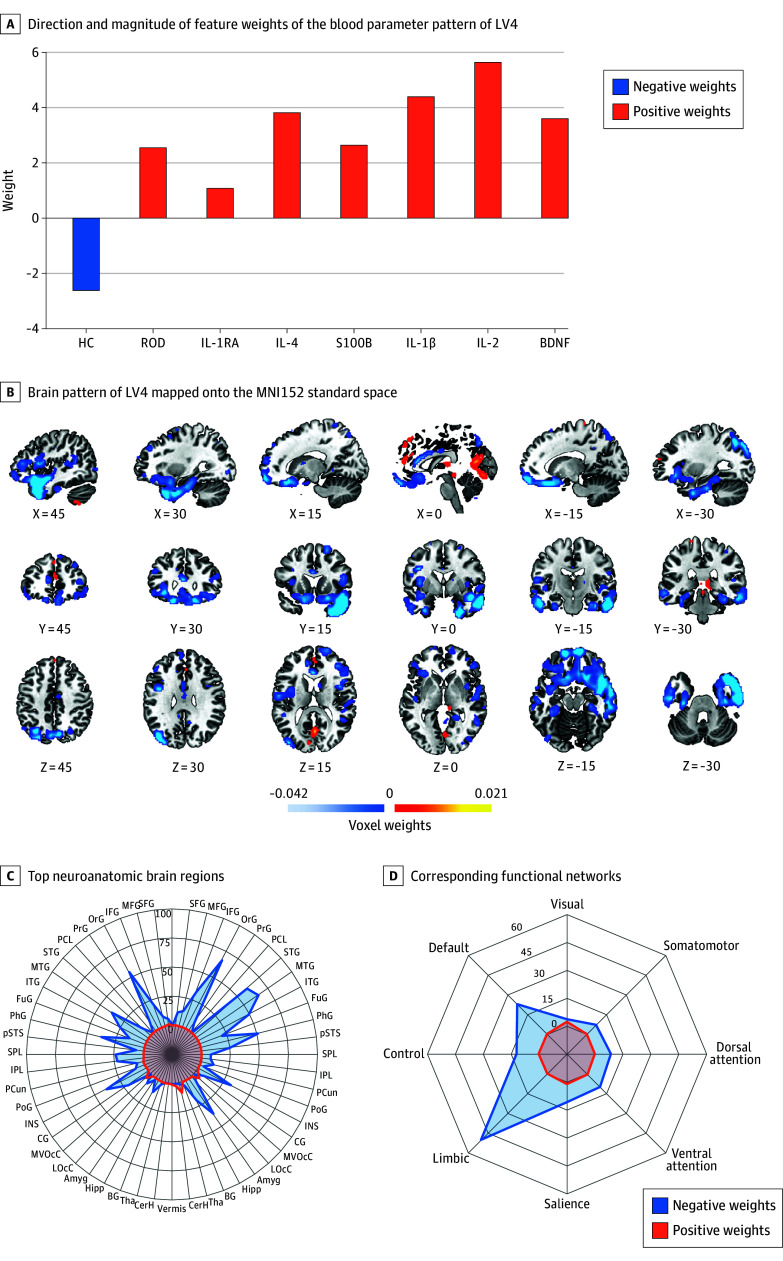
Depression Signature A, The bar plot displays both direction and magnitude of feature weights of the blood parameter pattern of latent variable 4 (LV4). If 2 feature weights had the same sign (ie, both positive or both negative), the respective features covaried positively with each other; an opposite direction of feature weights represents a negative covariation. Zero weights indicate that there was no significant contribution of the respective features to the covariance signature. Positive weights were assigned to recent-onset depression (ROD) status, interleukin 1 receptor antagonist (IL-1RA), interleukin 4 (IL-4), S100 calcium-binding protein B (S100B), interleukin 1β (IL-1β), interleukin 2 (IL-2), and brain-derived neurotrophic factor (BDNF). Healthy control status was negatively weighted. B, The brain pattern of LV4 was mapped onto the MNI152 standard space via the open-source 3-dimensional rendering software Connectome Workbench version 1.4.2. The spider plots highlight the top neuroanatomic brain regions (C; derived from the Brainnetome^34^ and Diedrichsen^35^ atlases) and corresponding functional networks (D; derived from an adapted, 8-network solution of the Yeo^36^ and Buckner^37^ atlases) according to the percentage of positive and negative voxels in these regions. Amyg indicates amygdala; BG, basal ganglia; CerH, cerebellum hemisphere; CG, cingulate gyrus; FuG, fusiform gyrus; Hipp, hippocampus; IFG, inferior frontal gyrus; INS, insular gyrus; IPL, inferior parietal lobule; ITG, inferior temporal gyrus; LOcC, lateral occipital cortex; MFG, middle frontal gyrus; MTG, middle temporal gyrus; MVOcC, medioventral occipital cortex; OrG, orbital gyrus; PCun, precuneus; PCL, paracentral lobule; PhG, parahippocampal gyrus; PoG, postcentral gyrus; PrG, precentral gyrus; pSTS, posterior superior temporal sulcus; SFG, superior frontal gyrus; SPL, superior parietal lobule; STG, superior temporal gyrus; Tha, thalamus.

Of 678 participants, 346 were female (51.0%) and 332 (49.0%) were male, and the median (IQR) age was 24.0 (20.9-28.9) years. SPLS analysis of the 678 individuals produced 4 significant LVs (LV1-LV4), which generalized across the discovery and the replication sample, representing multiple layers of associative effects between brain and blood parameter data (Figures 1 and 2; eTables 9-12 and 16 and eFigures 5-7 in Supplement 2; eTables 13 and 14 in Supplement 3).

#### LV1: Age 

The results for LV1 were as follows: full sample: ρ = 0.67, *P* < .001; discovery: ρ = 0.60, *P* < .001; and replication: ρ = 0.65, *P* < .001. The blood parameter pattern was primarily driven by age, with additional, smaller contributions from BMI, ROP, and CHR-P status, TNF-α, CRP, IL-6, and IFN-γ. The brain pattern yielded negative weights in the frontal, insular, and cingulate gyri. At the network level, these regions corresponded to the default, salience, and ventral attention networks.

#### LV2: Sex and Image Quality Rating 

The results for LV2 were as follows: full sample: ρ = 0.534, *P* < .001; discovery: ρ = 0.56, *P* < .001; and replication: ρ = 0.48, *P* < .001. The blood parameter pattern showed positive weights for male sex, image quality rating, and TGF-β, while female sex was negatively weighted. The brain pattern included positive weights for the thalami, posterior temporal sulci, postcentral, parahippocampal, fusiform and cingulate gyri, and hippocampi. At the network level, positive weights were found for the limbic network, while negative weights were observed for the control, somatomotor, dorsal attention, and default mode networks.

#### LV3: Psychosis 

The results for LV3 were as follows: full sample: ρ = 0.27, *P* = .002; discovery: ρ = 0.27, *P* < .001; and replication: ρ = 0.19, *P* < .005. The blood parameter pattern revealed that ROP status was associated with higher age, BMI, IL-6, and TNF-α and lower CRP levels compared to CHR-P status. The corresponding brain pattern showed an association between ROP status and increased GMV in the middle and inferior temporal gyri, precunei, lateral occipital cortices, and thalami and decreased GMV in the precentral and postcentral gyri, superior parietal lobules, hippocampi, basal ganglia, cerebellar hemispheres, and vermis compared to individuals with CHR-P. At the network level, positive weights mapped onto the visual system, dorsal attention, somatomotor, and default mode networks, whereas the control and ventral attention networks yielded predominantly negative weights.

#### LV4: Depression 

The results for LV4 were as follows: full sample: ρ = 0.19, *P* = .021; discovery: ρ = 0.22, *P* < .001; and replication: ρ = 0.21, *P* < .001. The blood parameter pattern showed an association between ROD status and higher levels of IL-1RA, IL-4, S100B, IL-1β, IL-2, and BDNF compared to HC individuals. ROD status was associated with GMV reductions in temporal, insular, and limbic gyri, along with the insular and parahippocampal gyri, posterior superior temporal sulci, amygdala, and hippocampi. These weights mapped onto limbic, default, control, salience, and ventral attention networks.

### Exploration of SPLS Signatures Using SVM-C

#### LV3: Psychosis 

**Figure 3.**
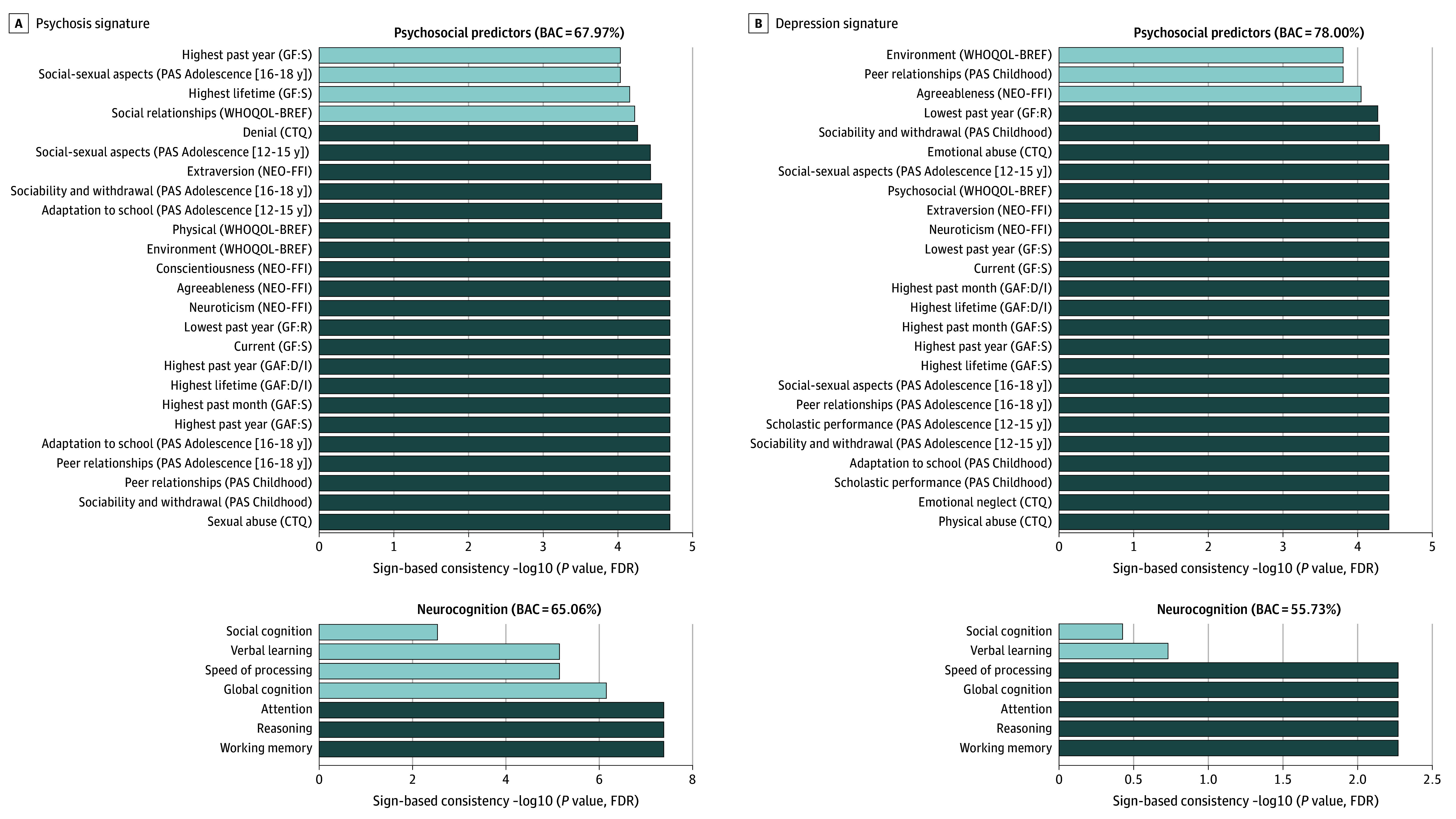
Post Hoc Exploration of the Psychosis and Depression Signature Prediction of latent variables (LVs) 3 (A) and 4 (B) high and low scorers using Support Vector Machine Classification with psychosocial (ie, Childhood Trauma Questionnaire [CTQ], Global Assessment of Functioning, Disability and Impairment [GAF:D/I], Global Assessment of Functioning, Symptoms [GAF:S], Global Functioning, Role [GF:R], Global Functioning, Social [GF:S], Premorbid Adjustment Scale [PAS], NEO Five-Factor Inventory [NEO-FFI], World Health Organization Quality of Life–Brief Version [WHOQOL-BREF]) (LV3: balanced accuracy [BAC], 67.97%; LV4: BAC, 78.00%), and neurocognitive data (ie, social cognition, verbal learning, speed of processing, global cognition, attention, reasoning, and working memory) (LV3: BAC, 65.06%; LV4: BAC, 55.73%). The significance of the predictive features was assessed by means of sign-based consistency. FDR indicates false discovery rate.

The results for LV3 are displayed in Figure 3A, eTables 18-21 and eFigures 8-9 in Supplement 2, and eTable 17 in Supplement 3. Psychosocial data (CTQ [sexual abuse and denial], NEO-FFI [neuroticism, agreeableness, conscientiousness, and extraversion], PAS, GAF, and GF) were predictive of the psychosis signature with a BAC of 67.2% (area under the receiver operating characteristic curve [AUC] = 0.71; *P* < .002), neurocognitive features (working memory, reasoning, and attention) with a BAC of 65.1% (AUC = 0.71; *P* < .002). Medication (BAC = 44.92%; AUC = 0.41; *P* = .86) did not significantly predict the psychosis signature.

#### LV4: Depression

The results for LV4 are displayed in Figure 3B, eTables 18 and 22-24 and eFigures 8-9 in Supplement 2, and eTable 17 in Supplement 3. Psychosocial data (CTQ [physical abuse, emotional neglect, and emotional abuse], NEO-FFI [neuroticism and extraversion], PAS, GAF, and GF) were predictive of the depression signature with a BAC of 78.0% (AUC = 0.83; *P* < .002). Neurocognitive performance (BAC of 55.73%, AUC = 0.61; *P* = .12) and medication (BAC = 52.06%; AUC = 0.63, *P* = .32) did not yield any significant prediction models.

## Discussion

In this cross-sectional study, we investigated brain-blood patterns in early-stage depression and psychosis using data from the PRONIA study. We identified 4 brain-blood signatures, the first 2 of which were related to age (LV1) as well as sex and MRI quality effects (LV2) (eResults in Supplement 2), while the remaining 2 pointed toward a dichotomous separation of psychotic (LV3) and depressive (LV4) disorders. All signatures generalized across the discovery and the replication sample.

### Psychosis Signature

The psychosis signature distinguished individuals with CHR-P from those with ROP based on a peripheral blood parameter profile of the proinflammatory cytokines IL-6 and TNF-α^15^, as well as CRP—an acute-phase protein considered a nonspecific, indirect marker of low-grade systemic inflammation, albeit with inconsistent findings.^13,49^ Individuals with CHR-P had higher levels of CRP, which could reflect a biological stress response to a changing interaction with and perception of the environment.^50^ In contrast, those with ROP had higher IL-6 and TNF-α and lower CRP levels. Both IL-6 and TNF-α were previously found to be elevated in drug-naive individuals with first-episode psychosis.^15^ TNF-α contributes to IL-6 production,^51^ which is consistently elevated in psychosis.^12^ Moreover, elevated IL-6 alongside lower CRP suggests a shift toward IL-6 transsignaling over classical signaling,^12,52^ previously found to be associated with psychosis risk^13^ through reduced membrane-bound receptor expression.^12^

IL-6 and TNF-α increase brain endothelial permeability,^53^ which could induce brain structure alterations.^54^ Indeed, the psychosis signature revealed a GMV pattern predominantly in the cortico-thalamo-cerebellar circuitry, whose disruption may be a key mechanism in psychosis.^55^ GMV increases mapped to visual and dorsal attention networks; decreases involved control and ventral attention networks, key circuits in sensory integration and salience attribution—hallmarks of full-blown psychosis.^56,57^ Specifically, elevated IL-6 was associated with GMV increases in the middle temporal gyrus and thalamus, potentially reflecting inflammation-associated pseudothickening in sensory integration regions^18^ and aligning with previous evidence finding an association between genetically predicted IL-6 with GMV in the middle temporal gyrus.^12^ Furthermore, elevated IL-6/TNF-α and reduced hippocampal volume suggest impaired neurogenesis via microglial activation.^58^ Overall, the psychosis signature indicates in CHR-P an unspecific, potentially stress-related state (CRP), whereas full-blown psychosis has a signal of (potentially persistent) low-grade trait inflammation (IL-6 transsignaling and TNF-α) and is associated with a brain structural shift of GMV patterns in the cortico-thalamo-circuit, which might serve as a neurobiological marker for delineating different states of psychosis.

### Depression Signature

The depression signature separated individuals with ROD from HC individuals via elevated proinflammatory (IL-1β and IL-2) and anti-inflammatory (IL-1RA, IL-2, and IL-4) cytokines as well as S100B and the neurotrophic BDNF.^59^ Acute phases of depression are accompanied by an immune-inflammatory response with an elevation of proinflammatory cytokines, such as IL-1β, and acute phase proteins, which subsequently triggers anti-inflammatory agents, including IL-1RA and IL-4.^60^ This cytokine profile suggests a mixed pro- and anti-inflammatory state, with IL-1RA potentially modulating IL-1β–driven inflammation,^60^ and the pleiotropic IL-2—implicated in both pro- and anti-inflammatory pathways^49,61^ and previously shown to be elevated in individuals with post–COVID-19 depression.^62^ Moreover, the depression signature yielded elevated S100B and BDNF. S100B was previously proposed as a marker of acute depression^63^ and appears to have neurotrophic as well as cytokinelike features,^64^ thereby triggering inflammatory responses, including the production of IL-1β. The observed S100B and BDNF elevations may therefore reflect early neuroinflammatory and compensatory neurotrophic processes in acute depression, consistent with the proposed involvement of BDNF in the therapeutic mechanisms of antidepressants.^65^ The limited permeability of the blood-brain barrier to large peptides such as S100B and BDNF, together with evidence that this barrier may be disrupted in psychosis and depression,^66^ creates uncertainty over whether elevated blood levels reflect systemic inflammation or direct effects on the brain. Since we found these proteins to be associated with brain structural changes, further studies using animal and in vitro models are needed to clarify their mechanistic role.^11^ Overall, the depression signature linked this complex pattern to GMV decreases in the limbic system, in which the amygdala and the hippocampus are key components of emotion processing, memory formation, fear conditioning, and social behavior.^67,68^ These findings support limbic-cortical dysregulation as a core neurobiological feature of depression.^68^ In summary, the depression signature reveals a potential interaction between immune-inflammatory (IL-1β and IL-2) and compensatory immune-regulatory (IL-1RA, IL-2, and IL-4) processes linked with potential neurotrophic adaptation and may connect this to characteristic GMV losses in the limbic system, highlighting the complex, yet distinct immune-neurobiological imprint of early-stage depression.

### Predictors of Blood-Brain Signatures

SVM-C modeling of psychosocial predictors supports prior evidence of a differential effect of childhood trauma (CTQ),^31^ with sexual abuse predicting the psychosis signature and physical abuse and emotional trauma predicting the depression signature. This aligns with prior work linking emotional trauma-related GMV alterations to depression^29^ and suggesting a particularly strong association between sexual abuse and psychosis-related symptoms and outcomes, including hallucinations and transition to psychosis.^69,70^ Furthermore, both psychosis and depression signatures were significantly predicted by premorbid adjustment (PAS) and past and present levels of functioning (GAF and GF), reiterating the link between psychosis, depression, and functional impairment across the lifespan, even before clinical manifestation.^1,2,3^ In addition, the personality traits (NEO-FFI) neuroticism and extraversion predicted both signatures; conscientiousness and agreeableness were unique to depression. Impairments in working memory, reasoning, and attention predicted only the psychosis signature, highlighting its stronger cognitive footprint.^49^

While some blood markers are known to change with pharmacological treatment, meta-analytic evidence indicates that others remain unaffected.^28,71^ The PRONIA cohort, in which individuals with CHR-P, ROP, and ROD were recruited under strict criteria to ensure minimal medication exposure, provides a unique context for such an investigation. The absence of medication effects on our identified signatures is therefore a novel finding, which suggests early illness-related rather than treatment-driven patterns. Overall, these findings challenge fully dimensional models of psychopathology^72^ by revealing distinct early-stage neurobiological profiles that separate psychosis and depression.

### Limitations

A key limitation of our study is the associative nature of the SPLS signatures, precluding any causal or mechanistic inference, while a longitudinal design is required to assess their temporal stability. SPLS cannot model nonlinear associations among variables—kernel-based methods could capture such effects. Furthermore, our sample was confined to participants in acute disease phases, precluding conclusions about the remissive phases of psychosis and depression. Although our ROD sample did not include individuals with psychotic features, limited evidence suggests that conditions at the intersection of psychotic and depressive disorders, such as major depressive disorder with psychotic features, may show distinct inflammatory patterns from nonaffective psychosis, warranting further investigation.^73^ Moreover, since we did not explicitly model transdiagnostic dimensions—such as symptom severity or level of functioning—across groups, some shared biological factors or transdiagnostic groups may have gone undetected. Additionally, our hypothesis-driven selection of blood parameters may have introduced selection bias, and external validation of the models is necessary.

## Conclusions

In summary, our study contributes new evidence to the ongoing debate on whether affective and psychotic disorders are better understood through a categorical or dimensional model and gives unique evidence for the relevance of peripheral measures of inflammation on brain structure at early stages. Our findings suggest distinct neurobiological signatures in depression and psychosis from early disease stages and emphasize the potential for biologically guided diagnosis and tailored interventions at the earliest point of care.

## Notes

Supplement 1. eMethods eResults eTable 1. Strengthening the Reporting of Observational Studies in Epidemiology (STROBE) reporting guideline eTable 2. Characteristics of Recruiting Institutions eTable 3. MR Scanner Systems and Structural MRI Sequence Parameters Used at the Respective PRONIA Sites eTable 4. Distribution of Study Participants Across Sites eTable 5. Group-level Differences between PRONIA Individuals Included and Not Included in the Analysis eTable 6. Group-level Multiple Comparison Tests for Sociodemographic, Clinical and Blood Parameter Differences eTable 7. Group-level Differences between Individuals from Discovery and Replication Sample eTable 8. Neurocognitive Test Battery eTable 9. Weight Vectors of the Blood Parameter Signatures of LV1-LV4 eTable 10. Number Of Features in the Brain and Blood Parameter Weight Vectors Before and After Bootstrapping eTable 11. Comparison of Latent Scores between Discovery and Replication Sample eTable 12. Comparison of Latent Scores between CHR-P Subgroups eTable 15. Atlas Readouts from LV1-LV4 for Large-scale Brain Networks eTable 16. Correlation Coefficient between Latent Scores in Discovery and Replication Sample eTable 18. Number of High- and Low-Scorers per SVM-C Prediction Model eTable 19. Comparison of Psychosocial Predictors between LV3 Low- and High-Scorers eTable 20. Comparison of Neurocognitive Predictors between LV3 Low- and High-Scorers eTable 21. Comparison of Medication Predictors between LV3 Low- and High-Scorers eTable 22. Comparison of Psychosocial Predictors between LV4 Low- and High-Scorers eTable 23. Comparison of Neurocognitive Predictors between LV4 Low- and High-Scorers eTable 24. Comparison of Medication Predictors between LV4 Low- and High-Scorers eFigure 1. Observational Study Design of PRONIA eFigure 2. CONSORT Chart and Overview of Analysis Steps eFigure 3. Histogram of CAT12 Image Quality Rating (IQR) of the Study Sample eFigure 4. Nested Cross-Validation Framework eFigure 5. Age-informed Signature of LV1 eFigure 6. Sex-and IQR-informed Signature of LV2 eFigure 7. Group-level Analysis of the Most Salient Brain and Blood Parameter Features Across All Four LVs eFigure 8. Medication-based Prediction of LV3 and LV4 High- and Low-Scorers eFigure 9. Receiver Operating Characteristic (ROC) Curves for SVM Predictive Models of High- and Low-Scorers on LV3 and LV4 eReferences

Supplement 2. eTable 13. Broad Atlas Readouts from LV1-LV4 for Neuroanatomical Brain Regions eTable 14. Detailed Atlas Readouts from LV1-LV4 for Neuroanatomical Brain Regions eTable 17. SVM-C Performance Metrics

Supplement 3. PRONIA Consortium members

Supplement 4. Data sharing statement
