# Supplementary material for: Multivariate Brain-Blood Signatures in Early-Stage Depression and Psychosis
Source: JAMA Psychiatry. 2025 Dec 17;83(2):172–84. doi: 10.1001/jamapsychiatry.2025.3803 (PMC12712837; doi:10.1001/jamapsychiatry.2025.3803)
Supplement: Supplement 1. — eMethods eResults eTable 1. Strengthening the Reporting of Observational Studies in Epidemiology (STROBE) reporting guideline eTable 2. Characteristics of Recruiting Institutions eTable 3. MR Scanner Systems and Structural MRI Sequence Parameters Used at the Respective PRONIA Sites eTable 4. Distribution of Study Participants Across Sites eTable 5. Group-level Differences between PRONIA Individuals Included and Not Included in the Analysis eTable 6. Group-level Multiple Comparison Tests for Sociodemographic, Clinical and Blood Parameter Differences eTable 7. Group-level Differences between Individuals from Discovery and Replication Sample eTable 8. Neurocognitive Test Battery eTable 9. Weight Vectors of the Blood Parameter Signatures of LV1-LV4 eTable 10. Number Of Features in the Brain and Blood Parameter Weight Vectors Before and After Bootstrapping eTable 11. Comparison of Latent Scores between Discovery and Replication Sample eTable 12. Comparison of Latent Scores between CHR-P Subgroups eTable 15. Atlas Readouts from LV1-LV4 for Large-scale Brain Networks eTable 16. Correlation Coefficient between Latent Scores in Discovery and Replication Sample eTable 18. Number of High- and Low-Scorers per SVM-C Prediction Model eTable 19. Comparison of Psychosocial Predictors between LV3 Low- and High-Scorers eTable 20. Comparison of Neurocognitive Predictors between LV3 Low- and High-Scorers eTable 21. Comparison of Medication Predictors between LV3 Low- and High-Scorers eTable 22. Comparison of Psychosocial Predictors between LV4 Low- and High-Scorers eTable 23. Comparison of Neurocognitive Predictors between LV4 Low- and High-Scorers eTable 24. Comparison of Medication Predictors between LV4 Low- and High-Scorers eFigure 1. Observational Study Design of PRONIA eFigure 2. CONSORT Chart and Overview of Analysis Steps eFigure 3. Histogram of CAT12 Image Quality Rating (IQR) of the Study Sample eFigure 4. Nested Cross-Validation Framework eFigure 5. Age-informed Signature of [file jamapsychiatry-e253803-s001.pdf]

Supplemental Online Content

Popovic D, Weyer C, Dwyer DB, et al; PRONIA Consortium. Multivariate brain-blood signatures in early-stage depression and psychosis. *JAMA Psychiatry*. Published online December 17, 2025. doi:10.1001/jamapsychiatry.2025.3803

eMethods

eResults

eTable 1. Strengthening the Reporting of Observational Studies in Epidemiology (STROBE) reporting guideline

eTable 2. Characteristics of Recruiting Institutions

eTable 3. MR Scanner Systems and Structural MRI Sequence Parameters Used at the Respective PRONIA Sites

eTable 4. Distribution of Study Participants Across Sites

eTable 5. Group-level Differences between PRONIA Individuals Included and Not Included in the Analysis

eTable 6. Group-level Multiple Comparison Tests for Sociodemographic, Clinical and Blood Parameter Differences

eTable 7. Group-level Differences between Individuals from Discovery and Replication Sample

eTable 8. Neurocognitive Test Battery

eTable 9. Weight Vectors of the Blood Parameter Signatures of LV1-LV4

eTable 10. Number Of Features in the Brain and Blood Parameter Weight Vectors Before and After Bootstrapping

eTable 11. Comparison of Latent Scores between Discovery and Replication Sample

eTable 12. Comparison of Latent Scores between CHR-P Subgroups

eTable 15. Atlas Readouts from LV1-LV4 for Large-scale Brain Networks

eTable 16. Correlation Coefficient between Latent Scores in Discovery and Replication Sample

eTable 18. Number of High- and Low-Scorers per SVM-C Prediction Model

eTable 19. Comparison of Psychosocial Predictors between LV3 Low- and High-Scorers

eTable 20. Comparison of Neurocognitive Predictors between LV3 Low- and High-Scorers

eTable 21. Comparison of Medication Predictors between LV3 Low- and High-Scorers

© 2025 Popovic D et al. *JAMA Psychiatry*.

eTable 22. Comparison of Psychosocial Predictors between LV4 Low- and High-Scorers

eTable 23. Comparison of Neurocognitive Predictors between LV4 Low- and High-Scorers

eTable 24. Comparison of Medication Predictors between LV4 Low- and High-Scorers

eFigure 1. Observational Study Design of PRONIA

eFigure 2. CONSORT Chart and Overview of Analysis Steps

eFigure 3. Histogram of CAT12 Image Quality Rating (IQR) of the Study Sample

eFigure 4. Nested Cross-Validation Framework

eFigure 5. Age-informed Signature of LV1

eFigure 6. Sex-and IQR-informed Signature of LV2

eFigure 7. Group-level Analysis of the Most Salient Brain and Blood Parameter Features Across All Four LVs

eFigure 8. Medication-based Prediction of LV3 and LV4 High- and Low-Scorers

eFigure 9. Receiver Operating Characteristic (ROC) Curves for SVM Predictive Models of High- and Low-Scorers on LV3 and LV4

eReferences

This supplemental material has been provided by the authors to give readers additional information about their work.

© 2025 Popovic D et al. *JAMA Psychiatry*.

eMethods 5
PRONIA Study Design 5
Detailed Sample Determination 5
Clinical and Sociodemographic Data 6
MRI Harmonization and Data Acquisition 6
MRI Preprocessing Pipeline 6
MRI Data Quality Assurance 6
Neurocognitive Battery 7
Sparse Partial Least Squares (SPLS) Algorithm 7
SPLS Cross-Validation Framework 10
Support Vector Machine Classification (SVM-C) 10
Peripheral Blood Parameter Assays 11
Visualization and Atlas Mapping of Neuroanatomic Weight Vectors 11
Blood Parameter- and Brain-level Correlations 11
eResults 12
Group-Level Sociodemographic and Clinical Differences at Baseline 12
SPLS Analysis: LV1 and LV2 12
SVM-C Results 12
Correlation Analysis Between Blood Parameter Features and GMV 13
eTables 14
eTable 1. Strengthening the Reporting of Observational Studies in Epidemiology (STROBE) reporting guideline ..... **Error! Bookmark not defined.**
eTable 2. Characteristics of Recruiting Institutions 17
eTable 3. MR Scanner Systems and Structural MRI Sequence Parameters Used at the Respective PRONIA Sites 18
eTable 4. Distribution of Study Participants Across Sites 19
eTable 5. Group-level Differences between PRONIA Individuals Included and Not Included in the Analysis20
eTable 6. Group-level Multiple Comparison Tests for Sociodemographic, Clinical and Blood Parameter Differences 22
eTable 7. Group-level Differences between Individuals from Discovery and Replication Sample 28
eTable 8. Neurocognitive Test Battery 31
eTable 9. Weight Vectors of the Blood Parameter Signatures of LV1-LV4 32
eTable 10. Number Of Features in the Brain and Blood Parameter Weight Vectors Before and After Bootstrapping 32
eTable 11. Comparison of Latent Scores between Discovery and Replication Sample 33
eTable 12. Comparison of Latent Scores between CHR-P Subgroups 34
eTable 13. Broad Atlas Readouts from LV1-LV4 for Neuroanatomical Brain Regions 35
eTable 14. Detailed Atlas Readouts from LV1-LV4 for Neuroanatomical Brain Regions 35
eTable 15. Atlas Readouts from LV1-LV4 for Large-scale Brain Networks 36
eTable 16. Correlation Coefficient between Latent Scores in Discovery and Replication Sample 38
eTable 17. SVM-C Performance Metrics 39
eTable 18. Number of High- and Low-Scorers per SVM-C Prediction Model 39

© 2025 Popovic D et al. *JAMA Psychiatry*.

eTable 19. Comparison of Psychosocial Predictors between LV3 Low- and High-Scorers 40
eTable 20. Comparison of Neurocognitive Predictors between LV3 Low- and High-Scorers 42
eTable 21. Comparison of Medication Predictors between LV3 Low- and High-Scorers............................... 42
eTable 22. Comparison of Psychosocial Predictors between LV4 Low- and High-Scorers 43
eTable 23. Comparison of Neurocognitive Predictors between LV4 Low- and High-Scorers 45
eTable 24. Comparison of Medication Predictors between LV4 Low- and High-Scorers............................... 45
eFigures 46
eFigure 1. Observational Study Design of PRONIA 46
eFigure 2. CONSORT Chart and Overview of Analysis Steps... 47
eFigure 3. Histogram of CAT12 Image Quality Rating (IQR) of the Study Sample 48
eFigure 4. Nested Cross-Validation Framework... 49
eFigure 5. Age-informed Signature of LV1 50
eFigure 6. Sex-and IQR-informed Signature of LV2 52
eFigure 7. Group-level Analysis of the Most Salient Brain and Blood Parameter Features Across All Four LVs 54
eFigure 8. Medication-based Prediction of LV3 and LV4 High- and Low-Scorers 55
eFigure 9. Receiver Operating Characteristic (ROC) Curves for SVM Predictive Models of High- and Low-Scorers on LV3 and LV4 56
eReferences 57

© 2025 Popovic D et al. *JAMA Psychiatry*.

eMethods

PRONIA Study Design

The entire PRONIA cohort consists of a discovery sample (recruitment period February 2014-May 2017) for model generation and a replication sample (recruitment period May 2017-May 2019) for model validation, which were pooled for this analysis to reach a sufficient sample size. Study participants, who had received a structural magnetic resonance (MR) scan and provided at blood sample at baseline, were included in the analysis, resulting in a study sample of 678 participants. These 678 study participants were recruited following the standardized recruitment and ascertainment protocol (eFigure 1) of the PRONIA study (Personalized Prognostic Tools for Early Psychosis Management, https://www.pronia.eu/). The observational study protocol involved follow-up examinations every three months after the index ascertainment and was implemented by the eight PRONIA sites. As described in the main text, only data from the baseline observation were used for the analysis. Upon study enrolment, participants were pseudonymized twice, locally at each site and centrally at the level of the PRONIA portal. The PRONIA portal consists of a multi-user database hosting clinical and neurocognitive information, blood parameter data and defaced MR images obtained from the study participants. The data are organized into digital questionnaires, visits, and cases. The portal provides the case managers with a controlled web-based interface to enter and upload the different data into the respective questionnaires. Furthermore, the PRONIA consortium has implemented a PRONIA@home mobile device interface that allows the study participants to securely log into the portal and fill out the self-rating questionnaires of a given visit. Upon completion of the data entry across all questionnaires of a given visit, the data is checked by an automatic quality control procedure which executes approximately 1600 data integrity and dependency rules. These rules include 1) basic checking of missing data and data ranges, 2) checking of dependency within one questionnaire, 3) dependencies between two questionnaires within one visit, and 4) dependencies between two consecutive visits (such as consistency of dates). Detected errors are fed back to the respective case managers allowing for a manual correction of the respective issues. This process is re-iterated until the quality of the clinical questionnaires in the given visit is sufficient for the entire visit to be locked. A comprehensive battery of ascertainment tools was used within a longitudinal observational study design to generate a multi-modal phenotypic profile of each study participant (eFigure 1). The clinical part of the battery compiled questionnaires that capture sociodemographic, somatic, environmental, diagnostic, psychopathological, functional and quality-of-life related variables in the PRONIA study population, such as the childhood trauma questionnaire (CTQ)[1]. This battery was complemented by multi-domain neurocognitive and neuroimaging examinations as well as blood sampling for later genetic characterization, which were carried out at the baseline and 9-month follow-up timepoints (see initial PRONIA publication by Koutsouleris et al[2] for further information on the PRONIA study).

Detailed Sample Determination

From the PRONIA sample, data from 166 healthy control (HC) individuals, 172 individuals with clinical high-risk states for psychosis (CHR-P), 177 individuals with recent-onset of psychosis (ROP) and 163 patients with recent-onset of depression (ROD), recruited at eight sites in five countries (Munich, Muenster, Basel, Cologne, Birmingham, Turku, Udine, Milan), were obtained for this study (Table 1 in main text). All adult participants provided their written informed consent prior to study inclusion. Minor participants (defined at all sites as those younger than 18 years) provided written informed assent and their guardians written informed consent. The study was registered at the German Clinical Trials Register (DRKS00005042) and approved by the local research ethics committees in each location. General inclusion criteria were age between 15 and 40 years, sufficient language skills for participation as well as capacity to provide informed consent/assent. General exclusion criteria were an IQ below 70, current or past head trauma with loss of consciousness (> 5 minutes), current or past known neurological or somatic disorders potentially affecting the structure or functioning of the brain, current or past alcohol dependence, or polysubstance dependence within the past six months, and any medical indication against MRI. The CHR-P state was defined by either 1) cognitive disturbances (COGDIS) criteria assessed using the Schizophrenia Proneness Instrument (SPI-A/-CY[3,4]) and/or 2) ultra-high-risk (UHR) criteria for psychosis based on the Structured Interview for Prodromal Syndromes (SIPS[5]). CHR-P exclusion criteria were 1) antipsychotic medication for > 30 days (cumulative number of days) at or above minimum dosage of the "1st episode psychosis" range of DGPPN S3 ("Deutsche Gesellschaft für Psychiatrie und Psychotherapie, Psychosomatik und Nervenheilkunde e. V.", German Association for Psychiatry, Psychotherapy and Psychosomatics) guidelines[6] and 2) any intake of antipsychotic medication within the past 3 months before clinical baseline assessments at or above minimum dosage of the "1st episode psychosis" range of DGPPN S3 guidelines[6]. ROP participants had to meet the following criteria: 1) DSM-IV-TR affective or non-affective psychotic episode (lifetime), 2) criteria for DSM-IV-TR (Diagnostic and Statistical Manual of Mental Disorders, Text Revision) affective or non-affective psychotic episode fulfilled within past 3 months and 3) onset of psychosis within past 24 months. ROP exclusion criterion was antipsychotic medication longer than 90 days (cumulative number of days) with a daily dose rate at or above minimum dosage in the "1st episode psychosis" range of the DGPPN S3 guideline.[6] ROD patients were identified by 1) DSM-IV-TR major depressive episode (lifetime), 2) major depressive disorder criteria fulfilled within past three months and 3) duration of first depressive episode no longer than 24 months. Specific ROD exclusion criteria

were: 1) more than 1 major depressive episode, 2) antipsychotic medication for > 30 days (cumulative number of days) at or above minimum dosage of the "1st episode psychosis" range of the DGPPN S3 guidelines and 3) any intake of antipsychotic medication within the past 3 months before psychopathological baseline assessments at or above minimum dosage of the "1st episode psychosis" range of the DGPPN S3 guidelines.[6]

Clinical and Sociodemographic Data

Positive, negative and general symptoms were measured using the Positive and Negative Symptom Scale (PANSS)[7], depression using the Beck Depression Inventory (BDI-II)[8] and level of functioning using the Global Assessment of Functioning Symptoms and Disability/Impairment Scales (GAF:S and GAF:D/I)[9] and the Global Functioning Social and Role Scales (GF:S and GF:R)[10]. The premorbid adjustment scale (PAS) was applied to evaluate the level of functioning prior to disease onset during childhood and adolescence[11]. Childhood trauma was assessed using the Childhood Trauma Questionnaire (CTQ)[1] and personality traits via the NEO Five Factor Inventory (NEO-FFI)[12]. The WHO Quality of Life Short Version (WHOQOL-BREF) was applied to evaluate self-reported quality of life[13]. Furthermore, cognitive performance was assessed along six cognitive domains (social cognition, working memory, speed of processing, verbal learning, reasoning, attention) and a global cognition score, comparable to the Measurement and Treatment Research to Improve Cognition in Schizophrenia (MATRICS) consensus battery (eMethods, eTable 8)[14,15].

MRI Harmonization and Data Acquisition

When setting up the PRONIA study, we decided to generate an MRI database that would represent the MR scanner sequence heterogeneity encountered in clinical real-world. The aim of this strategy was to strengthen the generalizability and clinical applicability of the predictive models developed by our machine learning analyses. Thus, we agreed on a minimal harmonization protocol that required the PRONIA sites to only 1) acquire isotropic or nearly isotropic voxel sizes of preferably 1 mm resolution, 2) set the Field Of View (FOV) parameters accordingly to guarantee the full 3D coverage of the brain including all parts of the cerebellum, and 3) define the relaxation time (TR) and echo time (TE) as well as other imaging parameters in a way that would maximize the contrast between cortical ribbon and the white matter and enhance the signal-to-noise ratio in the images. Error! Reference source not found. lists the parameters defining the structural MR sequences used to examine in the PRONIA discovery sample participants.

MRI Preprocessing Pipeline

The manual of the CAT12 toolbox (https://neuro-jena.github.io/cat12-help/) details the processing steps applied to the structural images. These steps consist of:

1) A 1st denoising step based on Spatially Adaptive Non-Local Means (SANLM) filtering.[16]
2) An Adaptive Maximum A Posteriori (AMAP) segmentation technique, which models local variations of intensity distributions as slowly varying spatial functions and thus achieves a homogeneous segmentation across cortical and subcortical structures.[17]
3) A 2nd denoising step using Markov Random Field approach which incorporates spatial prior information of adjacent voxels into the segmentation estimation generated by AMAP.[17]
4) A Local Adaptive Segmentation (LAS) step, which adjusts the images for white matter (WM) inhomogeneities and varying gray matter (GM) intensities caused by differing iron content in e.g., cortical, and subcortical structures. The LAS step is carried out before the final AMAP segmentation.
5) A Partial Volume Segmentation algorithm that is capable of modeling tissues with intensities between GM and WM, as well as GM and cerebrospinal fluid (CSF) and is applied to the AMAP-generated tissue segments.
6) A high-dimensional DARTEL registration of the image to an MNI-template generated from the MRI data of 555 healthy controls in the IXI database (http://brain-development.org/)

MRI Data Quality Assurance

To assess homogeneity of the acquired MRI scans and assure a high standard of MRI data quality, we employed the homogeneity check option of the CAT12 toolbox. As part of the preprocessing, CAT12 calculates several individual quality measures for each MRI scan: NCR (Noise Contrast Ratio), ICR (Inhomogeneity Contrast Ratio) and RES (RMS resolution). These measures are combined into the weighted average image quality rating (IQR). The IQR measure is scaled from 0.5 to 10.5, where 0.5 is a 'perfect/excellent' score and 10.5 is deemed 'unacceptable/failed'. Values around 1 and 2 represent '(very) good' image quality, whereas values of 5 and higher indicate problematic images.[18] The data quality features were entered into the CAT12 "check homogeneity" module along with modulated (m) normalized (w) GM segments (p1). We then calculated the Mahalanobis distance between the mean correlation and weighted overall image quality. Mean correlation quantifies the homogeneity of all selected MRI data used for statistical analysis and is therefore a measure of image quality after pre-processing. The weighted overall image quality combines measurements of noise and spatial resolution of the images before pre-processing. Hence, calculating the Mahalanobis distance between these two measurements estimates image

© 2025 Popovic D et al. *JAMA Psychiatry*.

quality both before and after pre-processing. Following this approach, we only included cases with an overall image quality rating (IQR) of "good" to "very good" (eFigure 3). This led to the exclusion of one case, which deviated from the rest of the sample by more than two standard deviations. This protocol closely follows the general recommendation as given in the official CAT12-Manual (https://neuro-jena.github.io/cat12-help/). Since MRI data quality is influenced by the individual's age and often correlates with symptomatology[19,20] we assumed a potential dimensional impact of data quality in our sample of mentally ill adolescents and young adults, even after passing the data quality assurance protocol. Since machine learning techniques such as sparse partial least squares (SPLS) can potentially detect and be driven by such subtle yet confounding factors, we included IQR as a feature into the analysis to investigate possible mediating effects between the blood-marker based signatures and MRI data quality.

Neurocognitive Battery

The PRONIA neuropsychological battery was previously described in Koutsouleris et al.[2] The neuropsychological tests used for construction of the main cognitive scores can be found in eTable 8. Prior to the calculation of the main cognitive scores, we harmonized verbal learning tests between the sites because of inconsistencies between the tests used. Verbal learning was calculated for the majority of the PRONIA sample with the Rey Auditory Verbal Learning Test (RAVLT[21]), which was not available in Finnish, therefore the revised version of the Hopkins Verbal Learning Test-Revised (HVLT-R[22]) was included in the neuropsychological battery of the Turk study site. To make the two verbal learning calculations comparable, we translated the HVLT-R scores to RAVLT scores as described in Penzel et al.[23]

We calculated six out of seven cognitive domains from the MATRICS consensus cognitive battery[14,15]: social cognition, working memory, speed of processing, verbal learning, reasoning and attention. We did not include visual learning (as in the original MATRICS) in the cognitive scores' computation because no test in the PRONIA neuropsychological battery could be compared to either the Neuropsychological Assessment Battery, shape learning subtest[14], or the Brief Visuospatial Memory Test-revised[14]. Social cognition deviated from the tests selected in MATRICS and was calculated using the Diagnostic Analysis of Non-Verbal Accuracy (DANVA[24]). Working memory was computed by summing the forward and backward trials of the Auditory Digit Span (ADS-F&B[25]) – a very similar test to the digit sequencing subtest of the BACS included in MATRICS. To calculate a composite score of speed of processing, we relied on three tests also used in MATRICS and averaged two graphomotor tests (i.e., the Trail Making Test, TMT-A[26], and the Digit Symbol Substitution Test, DSS[25]) and one verbal test (i.e., semantic Verbal Fluency, VF[27], correct words in 60 seconds). To calculate verbal learning, we took the sum of the first three RAVLT trials, to enhance similarity with the HVLT-R as in MATRICS, which only has three repetitions. Reasoning was assessed with the Matrix subtest from the Wechsler Adult Intelligence Scale-fourth edition (WAIS IV[25]), raw scores, while attention using the Continuous Performance Test-Identical Pairs (CPT-IP[28]) as in MATRICS. After Z-score transformation of the cognitive domains, we computed a composite score of Global Cognition by calculating the aggregate average across the 6 standardized scores.

Sparse Partial Least Squares (SPLS) Algorithm

The SPLS algorithm used in this analysis follows the original publication of Monteiro et al[29] and has been described in a previous publication of our group.[30] Specifically, we used the open source toolbox (https://github.com/dpopovic30/spls_toolbox_compiled.git) by Popovic et al[31]. Like Partial Least Squares (PLS), SPLS requires two data matrices X and Y as inputs. In our study, X contains neuroimaging information (structural MRI data), while Y contains levels of blood parameters and additional features (age, sex, BMI, diagnostic status, IQR). n is the number of samples; p is the number of voxels and q is the number of blood parameter features. PLS provides insights into the brain's mechanisms by finding relationships between different measures (i.e., views) from the same participants, i.e., between neuroimaging and blood parameter data, in a clinical population. PLS identifies a projection or latent space containing the relevant information in both views by finding pairs of weight vectors (generally called u and v) which maximize the covariance between the projections of the two views[32]:

1) $$maximize_{\|u\|_2=\|v\|_2=1}\, Cov(Xu, Yv) = maximize_{\|u\|_2=\|v\|_2=1}\, u^T X^T Y v$$

The weight vector pair is also called a latent variable (LV) as it explains one specific associative effect between the two different views. More specifically, the weight vectors place weights on each feature in the blood parameter and the neuroimaging dataset, thus visualizing which features are associated with each other as well as the direction and the strength of this multivariate association. Hence, by studying this latent space, one can learn about the underlying relationship between blood parameter information and brain measures [29].

In contrast to regular PLS, SPLS enforces sparsity on the weight vectors u and v through hyperparameters c_u and c_v. c_u and c_v are the regularization hyperparameters that control the l_1-norm constraints of u and v, respectively. The l_1-norm constraints impose sparsity, which means that the lower the values of c_u and c_v are, the higher the sparsity in

© 2025 Popovic D et al. *JAMA Psychiatry*.

the respective view is [33]. This leads to the following optimization problem:

2) $maximize_{u,v}\ u^T X^T Y v\ subject\ to\ \|u\|_2^2 \leq 1, \|v\|_2^2 \leq 1, \|u\|_1 \leq c_u, \|v\|_1 \leq c_v$

Yet, this type of constraint can only select up to n features if $p > n$. Furthermore, it will remove features which might be relevant for the model but are correlated with other features which are already included. Zou and Hastie addressed this issue by adding the l_2-norm constraints[34]. For both l_1-norm and l_2-norm constraints to be active, the values of the hyperparameters must be between 1 and the square root of the number of features in the respective matrices. Therefore, the hyperparameter space is updated:

3) $1 \leq c_u \leq \sqrt{p}, 1 \leq c_v \leq \sqrt{q}$

Using the hyperparameter space of equation 3) and solving the optimization problem of equation 2) according to Witten et al. [33,35] leads to the following SPLS algorithm steps as described in Monteiro et al. [29]:

1. Let $C \leftarrow X^T Y$
2. Initialize v to have $\|v\|_2 = 1$
3. Repeat until convergence:
   a) Update u:
      i. $u \leftarrow Cv$
      ii. $u \leftarrow \frac{S(u, \Delta_u)}{\|S(u, \Delta_u)\|_2}$, where $\Delta_u = 0$ if this results in $\|u\|_1 \leq c_u$, otherwise Δ_u is set to be a positive constant such that $\|u\|_1 = c_u$
   b) Update v:
      i. $v \leftarrow C^T u$
      ii. $v \leftarrow \frac{S(v, \Delta_v)}{\|S(v, \Delta_v)\|_2}$, where $\Delta_v = 0$ if this results in $\|v\|_1 \leq c_v$, otherwise Δ_v is set to be a positive constant such that $\|v\|_1 = c_v$
4. If convergence is not reached after the iteration limit (default: 1000), return non-sparse weight vectors u and v

After a weight vector pair (h) is found by SPLS, its effect needs to be removed from the data, to look for the next possible weight vector pair ($h + 1$). This process is called matrix deflation. In this setup, projection deflation is used as it has been shown to outperform the classic Hoteling's deflation, which is also used in Principal Component Analysis[29,36,37]. For matrices X and Y, the deflation process from iteration h to iteration $h + 1$ is therefore computed as follows:

$$X_{h+1} \leftarrow X_h - (X_h u_h) u_h^T$$

$$Y_{h+1} \leftarrow Y_h - (Y_h v_h) v_h^T$$

The algorithm then uses the deflated matrices and looks for the next associative effect, i.e., the next LV. This way, SPLS iteratively provides LVs consisting of sparse weight vector pairs (u, v), uncovering several layers of associative effects within the dataset.

The second step of the SPLS algorithm involves the creation of latent scores. For every LV, weight vectors u and v are projected onto the matrixes X and Y, thus generating latent scores ε and ω.

$$\varepsilon_h = X u_h$$

$$\omega_h = Y v_h$$

These latent scores are finite numerical values, which represent the loading of each individual on these weight vectors, e.g., how high their GM probability in certain voxels is. Therefore, every individual can be represented within each LV space with its latent blood parameter and brain scores. These specific scores can then be used for post-hoc analyses to investigate the meaning and relevance of these individual loadings.

The models were generated and tested in a nested cross-validation (NCV) framework with 5 outer (X2, Y2) and 5 inner folds (X1, Y1) (eFigure 4). Individuals were randomly assigned to the fold structure, while a stratification according to study group was ensured. All inner and outer folds contained an equal distribution of the 4 study groups (HC, ROD, CHR-P, ROP) to avoid training on diagnosis-related effects or indirectly on site-related effects.

© 2025 Popovic D et al. *JAMA Psychiatry*.

Within the inner folds, a 20x20 point grid search of both hyperparameters was conducted covering the entire hyperparameter space, in which both l_1- and l_2-norm constraints are fulfilled: $1 \leq c_u \leq \sqrt{p}$, $1 \leq c_v \leq \sqrt{q}$ (with p features in matrix X and q features in matrix Y). Lower c_u and c_v values lead to a sparser solution, whereas higher c_u and c_v values amount to a denser solution. At the upper limit, the maximum values of hyperparameters are: $c_u = \sqrt{p}$, $c_v = \sqrt{q}$. An SPLS analysis with c_u and c_v reaching these maximum values is equal to a regular PLS analysis, where every feature receives a weight and no feature is removed, i.e., no zero weights are given. Hence, our hyperparameter grid search includes the computation of one regular non-sparse PLS model (with c_u and c_v at the maximum limits) and an array of sparse PLS versions as lower c_u and c_v values are tested. Therefore, in this framework, the non-sparse regular PLS solution competes against the sparse PLS solution in the hyperparameter optimization process. The weight vector pairs were generated using the training folds in the inner loop ($X1_{train}$, $Y1_{train}$):

$$(\boldsymbol{u}, \boldsymbol{v}) = \boldsymbol{spls}(\boldsymbol{X1}_{train}, \boldsymbol{Y1}_{train}, \boldsymbol{c_u}, \boldsymbol{c_v})$$

The model fit of the weight vector pair was then assessed by projecting them onto the testing folds ($\boldsymbol{X1}_{test}$, $\boldsymbol{Y1}_{test}$) in the inner loop and computing Spearman's correlation coefficient between the projections of the weight vectors $\boldsymbol{u}$ and $\boldsymbol{v}$ onto their respective data matrices $\boldsymbol{X1}_{test}$ and $\boldsymbol{Y1}_{test}$:

$$\boldsymbol{\rho} = |\boldsymbol{Corr}(\boldsymbol{X1}_{test}\boldsymbol{u}, \boldsymbol{Y1}_{test}\boldsymbol{v})|$$

This approach delivers a simple and transparent measure of how well the weight vectors align the matrices to each other, i.e., how well they can maximize the covariance. The median correlation coefficient was computed for each hyperparameter combination in the inner loop. Afterwards, the best hyperparameter combinations ($\boldsymbol{c}_{u-top}$, $\boldsymbol{c}_{v-top}$) with the highest median correlation coefficients ($\boldsymbol{\rho}_{top}$) were retrained on the entirety of all 10 folds of the inner loop to increase the sample size for training once more:

$$(\boldsymbol{u}_{top}, \boldsymbol{v}_{top}) = \boldsymbol{spls}(\boldsymbol{X2}_{train}, \boldsymbol{Y2}_{train}, \boldsymbol{c}_{u-top}, \boldsymbol{c}_{v-top})$$

The generalizability of the weight vector pairs ($\mathbf{u}_{top}$, $\mathbf{v}_{top}$) was tested by assessing the fit of their projections onto the previously held-out fold in the outer loop and thus computing the corresponding correlation coefficients ($\boldsymbol{\rho}_{max}$).

$$\boldsymbol{\rho}_{max} = |\boldsymbol{Corr}(\boldsymbol{X2}_{test}\boldsymbol{u}_{opt}, \boldsymbol{Y2}_{test}\boldsymbol{v}_{opt})|$$

Significance testing of this weight vector pair was achieved by permutation testing against $\boldsymbol{B}$ permutations. Within the fold structure of the outer loop, $\boldsymbol{B}$ permutated datasets were created by randomly reshuffling the order of participants in one matrix ($\boldsymbol{Yb2}$) thus destroying relationship between the two matrices. The final model with the optimized hyperparameters ($\boldsymbol{c}_{u-opt}$, $\boldsymbol{c}_{v-opt}$) was then retrained and tested in each of the $\boldsymbol{B}$ permuted datasets, thus generating weight vectors $\boldsymbol{u_b}, \boldsymbol{v_b}$:

$$(\boldsymbol{u_b}, \boldsymbol{v_b}) = \boldsymbol{spls}(\boldsymbol{X2}_{train}, \boldsymbol{Yb2}_{train}, \boldsymbol{c}_{u-opt}, \boldsymbol{c}_{v-opt})$$

$$\boldsymbol{\rho_b} = |\boldsymbol{Corr}(\boldsymbol{X2}_{test}\boldsymbol{u_b}, \boldsymbol{Y2}_{test}\boldsymbol{v_b})|$$

Significance testing of the LV was done by assessing how often the model based on the permuted dataset performed better or equal to the model trained on the original dataset:

$$\boldsymbol{p} = \frac{\boldsymbol{1} + \sum_{b=1}^{B} \boldsymbol{1}_{\rho_b \geq \rho_{max}}}{\boldsymbol{B} + \boldsymbol{1}}$$

As our framework consisted of 5 outer folds, this approach led to 5 different models (i.e., 5 weight vector pairs u and v) for each LV iteration. Of these 5 different models, we selected the one model with the best performance as measured by means of permutation testing, i.e., the model that exhibited the lowest *P* value. If this optimal model passed significance testing against the FDR-corrected *P* value for multiple testing (5 models = 5 tests), the LV was deemed significant and the next LV was computed. This concept is known as the omnibus hypothesis, which was also applied in the original method paper of the SPLS algorithm.[29] The SPLS algorithm is an iterative process, in which based on hyperparameters c_u and c_v, the weight vectors u and v are computed in dependence of each other. First, u and v are initialized as non-sparse weight vectors based on regular singular value decomposition. Then an iterative process is set in motion, where first an enforcement of sparsity is attempted on weight vector u in dependence of weight vector v. Then sparsity is enforced on v, based on the previously computed weight vector u. This iterative process is repeated, where u and v are sequentially updated based on each other's previous modification until convergence between the vectors is reached. Hence, every hyperparameter setup c_u and c_v leads to a unique process of finding converging weight vectors u and v that were generated in a dialectic manner. Thus, the multivariate information is contained in this highly specific combination of weight vectors u and v, with both vectors containing mathematical information of the other. This, in turn, makes weight vectors u and v from

© 2025 Popovic D et al. *JAMA Psychiatry*.

different models, such as in our 5x5 fold NCV, not suitable for usual merging techniques (weighted mean/mean/median merging or majority voting) as every vector u is dependent on the corresponding vector v. Therefore, we used the omnibus hypothesis to determine our final LV model out of the 5 computed within the NCV structure of each LV iteration. Using our 5x5-fold outer and inner cross-validation loops can lead to high variance in the results. After training on the inner loops and then testing on the outer loops, 5 models with 5 *P* values are obtained. A criterion is then needed to determine whether any statistically significant effects were indeed found. For this, we used the omnibus hypothesis, where a statistical test is performed *j*-times to test a null-hypothesis H_j. Following the omnibus approach, the combined hypothesis H_R over all tests j is: "All the hypothesis H_j are true". This hypothesis will be rejected if any of the H_j hypothesis is rejected.[38] In our specific case, the omnibus hypothesis states that if any of the 5 *P* values (obtained in the 5 outer folds) is statistically significant (corrected for multiple testing *j*-times), then the omnibus hypothesis will be rejected, and the detected effect will be deemed significant. Therefore, the omnibus hypothesis will be rejected if any of the 5 splits generates a *P* value below .05 (adjusted for multiple testing). Of all significant splits, the model with the lowest *P* value will be determined as the final LV model.[29] The computation ends as soon as none of the 5 splits of the LV iteration did not pass the test for significance, which renders the entire LV not significant. Since deflating the data matrices of non-significant effects would be not justified, the analysis pipeline stops after the first non-significant LV was detected.

SPLS Cross-Validation Framework

We embedded the SPLS algorithm in a nested cross-validation (CV) framework with study group stratification and five outer (CV_2) and five inner folds (CV_1) (eFigure 4)[39]. GMV data were adjusted for site effects by applying a partial correlation correction based on effects identified in the HC subgroup of the training fold and then applied to CV_1 test and CV_2 validation data[40]. The significance of each LV was assessed by applying the final model to a total of 5000 permuted versions of the original data set and comparing the latent score correlation coefficients. To evaluate the stability of the feature weights, we calculated the bootstrap ratio (BSR) for each feature in 500 bootstrap samples[41] **(**eTable 10). The effect of each significant LV was removed from the data matrices using projection deflation[29] before computing subsequent LVs, until the detection of the first nonsignificant LV.

Support Vector Machine Classification (SVM-C)

To ensure the generalizability of our model and prevent information leakage, we employed a NCV framework (eFigure 4)[2,42]. This prevents the leakage of information between the individuals used for model training and the ones used for testing[43]. Initially, we divided the data into training and test folds on the outer (CV2) cycle. Next, we further divided the resulting training folds into 10 inner (CV1) training and testing folds, resulting in a 10-by-10-fold NCV[42,44,45]. Model training, including hyperparameter optimization, was performed within the CV1 cycle, while model testing was exclusively conducted within the CV2 cycle. The CV1 cycle test data were utilized to select hyperparameter combinations that exhibited strong generalization capabilities for the models. Finally, the CV2 test data were used to assess the models' generalizability to new, unseen data. The NCV approach was extended to incorporate repeated NCV at both the CV2 and CV1 levels[39]. This was achieved by randomly permuting participants within their respective groups, resulting in 10 permutations. The CV cycle was repeated for each of these permutations. Our machine learning pipeline, NeuroMiner (v1.1; available at https://github.com/neurominer-git/NeuroMiner_1.1), encompassed the following steps: Each variable was scaled to a range of 0-1, features with zero variance were removed, missing values were imputed with the median of the 7 nearest neighbors. The classifier proceeded with a stepwise forward variable selection procedure[46], employing a linear support vector machine (SVM) [47] (i.e., LIBSVM 3.12 with instance weighting support) to identify the most relevant variables for prediction. This process aimed to identify a parsimonious combination of features that optimized the average classification performance across the CV1 training and testing data. Using the scaling obtained from the CV1 cycle the most discriminative variables were preprocessed and the trained model was applied to the corresponding CV2 fold. The outcome class of each individual (high- vs. low-scorers) was determined by majority voting across all ensemble models. The SVM optimized a hyperplane in the linear kernel space that maximized the separability between subjects most similar to low-scorers and those most similar to high-scorers (support vectors). The algorithm predicted the individuals' classification of the inner CV1 cycle based on this trained hyperplane by projecting their data into the learned kernel space and assessing their geometric distance to the decision boundary. This process yielded a decision value and a predicted classification label for each subject. This was then repeated for each combination of the SVM parameter C (misclassification cost) across a grid defined by the ranges C = [0.0156 - 16]. Given our repeated NCV framework, an ensemble of 100 models (n = 10 repetitions x k = 10 folds) for each CV2 partition was generated, which we refer to as the 'CV1 ensemble'. Additionally, by leveraging the 10 repetitions of the CV2 cycle, we could establish a final prediction of out-of-training class membership for a specific individual by amalgamating all CV1 ensembles into a larger CV2 ensemble. Notably, the given individual did not contribute to the model training and optimization at the CV1 level. This ensemble generation procedure has been previously described in our prior research[48] and represents a key aspect of the model generation and validation process implemented in NeuroMiner.

© 2025 Popovic D et al. *JAMA Psychiatry*.

Peripheral Blood Parameter Assays

The acquisition of peripheral blood parameters follows the standard procedure used through the PRONIA study, as previously described by Lalousis et al[49]: In accordance with a strict protocol, a 9 ml blood sample was collected from participants who gave their consent. The serum was collected in S-monovette serum tubes to isolate the serum. Whenever possible, the blood samples were obtained in the morning after a 12-hour fasting period. The samples were inverted 5-6 times and left at room temperature for 30 minutes to allow coagulation. After centrifugation at 2750g for 10 minutes, the serum was transferred into 8 cryovials, with each vial containing 300ul aliquots. The vials were then stored at -80°C until further analysis. At the same time as sampling, two 9 ml whole blood samples were collected from fasting participants using EDTA tubes. These blood samples were also stored at -80°C until genomic analysis. In March 2019, the samples were sent to the University of Birmingham Barnes Laboratory. The samples were not subjected to any freeze-thaw cycles before the analysis. Out of the samples included in the analysis, 83% were obtained before 12 noon, and 76% were collected from fasting individuals. When the blood was drawn, 95% of the participants were in a sitting position. On average, the serum aliquots were stored for 48.78 months until analysis (with a median storage duration of 45 months). All samples were assayed blind to subject information. An aliquot from each sample was centrifuged for 15 minutes at 15k x g and 4°C before analysis using the Luminex platform (Bio-Plex 200 system with Bio-Plex Manager software) using a commercial multiplex kit from Bio-Techne for IFN-γ, IL-1β, IL-1RA, IL-2, IL-4, IL-6, S100B, and TNF-α. Additionally, commercial singleplex kits from Bio-Techne were used for CRP, BDNF, and TGF-β1 following the manufacturer's instructions.

Visualization and Atlas Mapping of Neuroanatomic Weight Vectors

The neuroanatomic weight vectors of the LV were visualized as 3D MRI images using the SPM12 (Wellcome Department of Cognitive Neurology, London, UK[50]) and the Connectome Workbench v1.4.2 (https://humanconnectome.org/software/connectome-workbench). Readouts of specific anatomical atlas regions (eTable 13, eTable 14, eTable 15) were attained using the Brainnetome atlas[51,52] and the cerebellar atlas by Diedrichsen[53,54].

Blood Parameter- and Brain-level Correlations

To enhance interpretability of the blood parameter-brain signatures and bolster confidence that the brain-blood parameters signatures correspond to real-world characteristics, we further investigated the relationship between the blood parameter- and brain-level features of each LV by correlating the most strongly weighted (i.e., the most salient) brain region and the most strongly weighted blood parameter feature for each LV (Figure 1, Figure 2, eFigure 5eFigure 6, eFigure 7, eTable 9, eTable 13, eTable 14, eTable 15).

© 2025 Popovic D et al. *JAMA Psychiatry*.

eResults

Group-Level Sociodemographic and Clinical Differences at Baseline

Of the 1800 PRONIA participants fulfilling inclusion criteria, 678 (41.0%) had sufficient data to be included in the present analyses (eTable 5, eFigure 2). Of these, 346 (51.0%) were female, and the median age [IQR] age was 24.00 [20.90-28.85]. Significant group-level differences (Table 1 in main text, eTable 6) between the diagnostic subgroups (excluding HC) were found for age, BDI-II, PANSS total, positive and general subscale scores, GAF:S, GAF:D/I, GF:S, and GF:R within the past month, the NEO-FFI personality domains neuroticism, extraversion, agreeableness and conscientiousness, the QoL domains physical and psychosocial as well as the neurocognitive domains working memory, processing speed, verbal learning, reasoning and the global score. Antipsychotic and antidepressant treatment also differed significantly between subgroups.

SPLS Analysis: LV1 and LV2

LV1 contained a blood parameter pattern, which was dominated by age (eFigure 5A). The brain pattern consisted of negatively weighted GM volume (GMV) across predominantly cortical brain areas, including the frontal and temporal gyri as well as the insular and cingulate gyrus (eFigure 5B). They align with the general pattern of GMV reduction throughout the brain that is a natural part of the aging process, primarily observed in the frontal and temporal regions[55,56]. Interestingly, the rate and extent of GMV loss was shown to vary across different brain regions[57]. Several mechanisms have been proposed to contribute to this age-related GMV loss including a reduction in neuronal body size, a degeneration of the neuropil and the dendritic network as well as the resulting loss of synapses[57]. On a network level, the associated brain regions mapped onto the default mode network, which aligns with previous studies reporting a reduction in functional connectivity within components of the default mode network, supporting the idea that late-developing brain regions are more susceptible to the negative effects of aging[58]. A decreased connectivity of the default mode network has also been associated with age-related structural changes[59]. Furthermore, age was associated with ROP status and, inversely, with CHR-P status, which is most likely due to the significantly higher age of the ROP individuals compared to the CHR-P individuals in our sample (Table 1 in main text, eTable 6). In addition to IFN-γ, IL-6, TNF-α and CRP, which all received relatively small positive weights, BMI displayed a positive association with age. This aligns with previous, though so far limited evidence for an association between increasing age and BMI during early to middle adulthood[60].

LV2 yielded a blood parameter pattern (eFigure 6A), which was dominated by sex (0.66) and MR image quality (0.34). The corresponding brain pattern is spread rather sparsely across cortical areas (eFigure 6B-C), covering predominantly the default, the salience and the ventral attention network, and with the thalamus receiving the highest negative weighting (eFigure 6D). This aligns with previous findings of greater thalamic volume in women[61], but does not or only partially replicate other large-scale meta-analyses reporting either no sex differences in thalamic volume or higher volume in men compared to women[62,63]. These contradictory findings can – most likely – be attributed to differences in methodological design[64]. However, there is a growing body of evidence for substantial differences in GMV between sexes, but the underlying mechanisms remain uncertain and an active area of research[64–66]. Several factors have been proposed to contribute to these differences, including hormonal differences (e.g., testosterone), environmental (e.g., socialization) or genetic factors (e.g., sex chromosomes)[64,66]. Female and male individuals also displayed substantial differences with regard to the affected brain networks (eFigure 6D), which may be attributable to previously reported differences in behavior and cognition (e.g., social cognition, emotion regulation) between males and females[66,67]. These differences may, in turn, be associated with the organization of functional networks and can, accordingly, be linked to sex differences in GMV within these networks[66,68]. Our finding of an association between image quality and GMV is consistent with previous studies highlighting the influence of in-scanner motion during MRI acquisition on structural MRI measurements, including GMV, particularly in clinical populations but also in healthy control individuals[19,69–72]. This underscores the importance of including a measure of MR image quality as a feature in analyses investigating structural brain alterations, as not controlling for MR image quality may systematically bias the results of these studies[19,69–71].

SVM-C Results

While the psychosis (LV3) and depression (LV4) signatures were both significantly predicted by past and present levels of functioning (GAF, GF), differences emerged in other domains (Figure 3). Scholastic performance (PAS), for instance, was uniquely predictive of the depression signature, whereas peer relationships during childhood were predictive of the psychosis staging signature and only became a significant predictor for the depression signature during late adolescence. This suggests that both psychosis and depression, while partially overlapping, are characterized by different premorbid trajectories of psychosocial functioning.

Different modalities of perceived QoL (WHO-QOL) predicted LV3 and LV4 high- and low-scorers (Figure 3). The psychosis staging signature (LV3) was linked to physical and environmental QoL domains, covering physical

© 2025 Popovic D et al. *JAMA Psychiatry*.

aspects such as pain, energy, sleep, and mobility, as well as environmental factors such as safety and security, financial situation, leisure, and health care. The depression signature (LV4) on the other hand was linked to the psychological QoL domain, comprising emotional states, cognition, and self-esteem in depression.

Correlation Analysis Between Blood Parameter Features and GMV

To enhance interpretability of the blood parameter-brain signatures and bolster confidence that the blood parameter-brain signatures correspond to real-world characteristics, we further investigated the relationship between the blood parameter- and brain-level features of each LV (eFigure 7). Within each LV, we identified the most strongly weighted (i.e., the most salient) brain region (Figure 1-2, eFigure 5B-D, eFigure 6B-D, eTable 13, eTable 14), and the most strongly weighted blood parameter feature (eFigure 5A, eFigure 6A, eTable 9). From all 678 individuals, we then extracted their values in the most salient blood parameter feature as well as their GMV in the most salient brain region and performed a non-parametric correlation analysis between these two domains. This approach yielded significant associations across all 4 LVs. As an example, IL-2 and the right middle temporal gyrus (MTG) were the most strongly weighted blood parameter and brain features in LV4. They received inverse weightings, with IL-2 being strongly positively and the MTG strongly negatively weighted. Following the above-mentioned approach, we found that the individuals' levels of IL-2 were inversely correlated with their GMV in the MTG (eFigure 7). Thus, we are confident that our blood parameter-brain signatures do correspond to observable blood parameter and neuroanatomic characteristics. Hence, the diverse set of feature weights within an LV represents a complex network of covariance, which can be interrogated and confirmed by in-depth investigation of the underlying real-world features (i.e., levels of blood parameters, GMV).

© 2025 Popovic D et al. *JAMA Psychiatry*.

eTables

eTable 1. Strengthening the Reporting of Observational Studies in Epidemiology (STROBE) reporting guideline

	Item No	Recommendation	Page No
**Title and abstract**	1	(*a*) Indicate the study's design with a commonly used term in the title or the abstract	1
		(*b*) Provide in the abstract an informative and balanced summary of what was done and what was found	1
**Introduction**			
Background/rationale	2	Explain the scientific background and rationale for the investigation being reported	6-7
Objectives	3	State specific objectives, including any prespecified hypotheses	7-8
**Methods**			
Study design	4	Present key elements of study design early in the paper	8
Setting	5	Describe the setting, locations, and relevant dates, including periods of recruitment, exposure, follow-up, and data collection	8-9
Participants	6	(*a*) *Cohort study*—Give the eligibility criteria, and the sources and methods of selection of participants. Describe methods of follow-up	8, eMethods
		Case-control study—Give the eligibility criteria, and the sources and methods of case ascertainment and control selection. Give the rationale for the choice of cases and controls	
		Cross-sectional study—Give the eligibility criteria, and the sources and methods of selection of participants	
		(*b*) *Cohort study*—For matched studies, give matching criteria and number of exposed and unexposed	
		Case-control study—For matched studies, give matching criteria and the number of controls per case	
Variables	7	Clearly define all outcomes, exposures, predictors, potential confounders, and effect modifiers. Give diagnostic criteria, if applicable	9-10
Data sources/ measurement	8*	For each variable of interest, give sources of data and details of methods of assessment (measurement). Describe comparability of assessment methods if there is more than one group	9, eMethods
Bias	9	Describe any efforts to address potential sources of bias	

© 2025 Popovic D et al. *JAMA Psychiatry*.

Study size	10	Explain how the study size was arrived at	8, eMethods, eFigure 1
Quantitative variables	11	Explain how quantitative variables were handled in the analyses. If applicable, describe which groupings were chosen and why	
Statistical methods	12	(*a*) Describe all statistical methods, including those used to control for confounding	9-11, eMethods
		(*b*) Describe any methods used to examine subgroups and interactions	eMethods
		(*c*) Explain how missing data were addressed	eMethods
		(*d*) *Cohort study*—If applicable, explain how loss to follow-up was addressed	
		Case-control study—If applicable, explain how matching of cases and controls was addressed	
		Cross-sectional study—If applicable, describe analytical methods taking account of sampling strategy	
		(*e*) Describe any sensitivity analyses	
**Results**			
Participants	13*	(a) Report numbers of individuals at each stage of study—eg numbers potentially eligible, examined for eligibility, confirmed eligible, included in the study, completing follow-up, and analysed	eFigure 1
		(b) Give reasons for non-participation at each stage	eFigure 1
		(c) Consider use of a flow diagram	eFigure 1
Descriptive data	14*	(a) Give characteristics of study participants (eg demographic, clinical, social) and information on exposures and potential confounders	Table 1
		(b) Indicate number of participants with missing data for each variable of interest	
		(c) *Cohort study*—Summarise follow-up time (eg, average and total amount)	
Outcome data	15*	*Cohort study*—Report numbers of outcome events or summary measures over time	11-13
		Case-control study—Report numbers in each exposure category, or summary measures of exposure	
		Cross-sectional study—Report numbers of outcome events or summary measures	

© 2025 Popovic D et al. *JAMA Psychiatry*.

Main results	16	(*a*) Give unadjusted estimates and, if applicable, confounder-adjusted estimates and their precision (eg, 95% confidence interval). Make clear which confounders were adjusted for and why they were included	11-13
		(*b*) Report category boundaries when continuous variables were categorized	
		(*c*) If relevant, consider translating estimates of relative risk into absolute risk for a meaningful time period	
Other analyses	17	Report other analyses done—eg analyses of subgroups and interactions, and sensitivity analyses	
**Discussion**			
Key results	18	Summarise key results with reference to study objectives	13-16
Limitations	19	Discuss limitations of the study, taking into account sources of potential bias or imprecision. Discuss both direction and magnitude of any potential bias	16
Interpretation	20	Give a cautious overall interpretation of results considering objectives, limitations, multiplicity of analyses, results from similar studies, and other relevant evidence	16
Generalisability	21	Discuss the generalisability (external validity) of the study results	
**Other information**			
Funding	22	Give the source of funding and the role of the funders for the present study and, if applicable, for the original study on which the present article is based	tbd

*Give information separately for cases and controls in case-control studies and, if applicable, for exposed and unexposed groups in cohort and cross-sectional studies.

© 2025 Popovic D et al. *JAMA Psychiatry*.

eTable 2. Characteristics of Recruiting Institutions

Site	Institution Name	Country	Type of Service	Catchment Population	Screening population / year
Munich	Department of Psychiatry and Psychotherapy, Ludwig-Maximilian-University Munich	DE	Academic outpatient services including specialized service for early recognition of psychosis; tertiary care academic hospital	1,200,000	700
Cologne	Department of Psychiatry and Psychotherapy, University of Cologne	DE	Academic outpatient services including specialized service for early recognition of psychosis; tertiary care academic hospital	1,000,000	600
Muenster	Department of Mental Health, University of Muenster, Muenster, Germany	DE	Academic in- and outpatient services including service for early recognition of psychosis; tertiary care academic hospital	300,000	400
Basel	Department of Psychiatry and Psychotherapy, University of Basel	CH	Academic inpatient and outpatient services including specialized service for early recognition and intervention of psychosis; tertiary care academic hospital	500,000	200
Milan Niguarda	Department of Pathophysiology and Transplantation, University of Milan. Four recruitment hospitals: Niguarda, Policlinico, San Paolo, Villa San Benedetto Menni in Albese con Cassano	IT	Psychiatric outpatient services including specialized services for early recognition of psychosis and persons at high risk; Academic hospital, providing psychiatric inpatient services, psychiatric outpatient services and local services	600,000	1,000
Udine	Department of Psychiatry, University of Udine	IT	Psychiatric outpatient services, academic hospital, and local services. Tertiary care neuropsychiatric service	600,000	500
Birmingham	The University of Birmingham	UK	Academic specialized Early Intervention Service for Psychosis covering Birmingham and Solihull. Community and Inpatient	1,200,000	800
Turku	Department of Psychiatry, University of Turku	FI	Psychiatric outpatient and hospital services responsible for treatment of psychiatric patients in their catchment areas in the South-Western Finland	284,000	2,300

Previously published in Popovic et al.[30] and reprinted with permission.

© 2025 Popovic D et al. *JAMA Psychiatry*.

eTable 3. MR Scanner Systems and Structural MRI Sequence Parameters Used at the Respective PRONIA Sites

Site	Model	Field Strength	Coil Channels	Flip Angle	TR [ms]	TE [ms]	Voxel Size [mm]	FOV	Slice Number
Munich	Philips Ingenia	3T	32	8	9.5	5.5	0.97 x 0.97 x 1.0	250 x 250	190
Cologne	Philips Achieva	3T	8	8	9.5	5.5	0.97 x 0.97 x 1.0	250 x 250	190
Muenster	Siemens Magnetom PRISMA-FIT	3T	20	8	2130	2,28	1x1x1	256	192
Basel	SIEMENS Verio	3T	12	8	2000	3.4	1.0 x 1.0 x 1.0	256 x 256	176
Milan	Philips Achieva Intera	1.5T	8	12	Shortest (8.1)	Shortest (3.7)	0.93 x 0.93 x 1.0	240 x 240	170
Udine	Philips Achieva	3T	8	12	Shortest (8.1)	Shortest (3.7)	0.93 x 0.93 x 1.0	240 x 240	170
Birmingham	Philips Achieva	3T	32	8	8.4	3.8	1.0 x 1.0 x 1.0	288 x 288	175
Turku	Philips Ingenuity	3T	32	7	8.1	3.7	1.0 x 1.0 x 1.0	256 x 256	176

Previously published in Popovic et al. [30] and reprinted with permission. *Abbreviations:* FOV, Field of View; TR, Relaxation Time; TE, Echo Time.

© 2025 Popovic D et al. *JAMA Psychiatry*.

eTable 4. Distribution of Study Participants Across Sites

	All	CHR-P	HC	ROD	ROP
N	678	172	166	163	177
Site					
Munich	270	72	52	72	74
Milan	50	16	9	8	17
Basel	2	NA	1	NA	1
Cologne	174	32	57	49	36
Muenster	19	9	NA	4	6
Birmingham	9	4	1	3	1
Turku	99	25	23	13	38
Udine	55	14	23	14	4

Abbreviations: CHR-P, Clinical High-Risk for Psychosis; HC, Healthy Control; ROD, Recent-Onset Depression; ROP, Recent-Onset Psychosis.

© 2025 Popovic D et al. *JAMA Psychiatry*.

eTable 5. Group-level Differences between PRONIA Individuals Included and Not Included in the Analysis

	All	Excluded	Included	Test Statistic	η^2/V	P-value
N	1800	1122	678			
Sociodemographic Data						
Age	24.11 [20.65-28.76]	24.24 [20.44-28.74]	24.00 [20.90-28.85]	-0.31	< .01	1.00
Sex				0.03	< .01	1.00
Male sex, No. (%)	873 (48.5)	541 (48.22)	332 (48.97)			
Female sex, No. (%)	920 (51.11)	574 (51.16)	346 (51.03)			
BMI	22.58 [20.57-25.15]	22.59 [20.64-25.25]	22.50 [20.43-25.07]	0.76	< .01	1.00
Handedness (Right), No. (%)	1219 (67.72)	675 (60.16)	544 (80.24)	77.92	0.21	**4.08E-17**
Education Years	14 [12-17]	14 [12-16.5]	14 [12.288-17]	-1.77	< .01	1.00
Tobacco Use, No. (%)	562 (31.22)	305 (27.18)	257 (37.91)	3.42	0.05	1.00
Symptomatology						
BDI-II	14 [3-27]	11 [2-26]	17 [5-28]	-3.52	< .01	**.016**
PANSS						
Total	54 [44-68.5]	54 [43-68]	55 [45-69]	-0.95	< .01	1.00
Positive	11 [8-17]	11 [8-18]	11 [8-16]	0.2	< .01	1.00
Negative	13 [9-18]	12 [8-17]	13 [9-18]	-2.27	< .01	.953
General	29 [24-36]	29 [24-36]	29 [24-36]	-0.71	< .01	1.00
Level of Functioning						
GAF:S (Past Month)	57 [45-85]	61 [45-85]	55 [45-75]	4.27	0.01	**.001**
GAF:D/I (Past Month)	60 [45-81]	61 [45-85]	55 [41.5-80]	5.15	0.01	**4.74E-4**
GF:S (Current)	7 [6-8]	7 [6-8]	7 [6-8]	4.37	0.01	**4.74E-4**
GF:R (Current)	7 [5-8]	7 [5-8]	6 [5-8]	4.57	0.01	**1.85E-4**
NEO-FFI						
Neuroticism	25 [17-33]	24 [16-33]	26 [18-34]	-2.54	< .01	.416
Extraversion	26 [19-32]	27 [20-32]	24 [19-31]	3.77	< .01	**.006**
Openness	29 [24-33]	29 [24-33]	29 [25-33]	-0.1	< .01	1.00
Agreeableness	31 [26-35]	31 [27-35]	31 [26-36]	-0.45	< .01	1.00

© 2025 Popovic D et al. *JAMA Psychiatry*.

Conscientiousness	30 [24-35]	30.5 [24-36]	29 [23.5-34]	3.31	< .01	**.035**
WHOQOL-BREF						
Physical	26 [21-30]	27 [22-31]	25 [21-30]	3.79	< .01	**.006**
Psychological	19 [15-24]	21 [15-25]	18 [14-23]	4.44	0.01	**3.46E-4**
Social Relationships	10 [8-12]	11 [9-12]	10 [8-12]	2.51	< .01	.455
Environment	30 [27-34]	31 [27-34]	30 [27-34]	2.36	< .01	.703
CTQ						
Emotional Abuse	7 [5-10]	7 [5-10]	8 [6-11]	-3.86	< .01	**.004**
Physical Abuse	5 [5-6]	5 [5-6]	5 [5-6]	-1.86	< .01	1.00
Sexual Abuse	5 [5-5]	5 [5-5]	5 [5-5]	-1.89	< .01	1.00
Emotional Neglect	9 [6-13]	9 [6-13]	10 [7-14]	-2.17	< .01	1.00
Physical Neglect	6 [5-8]	6 [5-8]	6 [5-8]	-1.67	< .01	1.00
MATRICS battery						
Social Cognition	-0.01 [-0.42-0.82]	-0.01 [-0.42-0.82]	-0.01 [-0.42-0.82]	-0.23	< .01	1.00
Working Memory	-0.13 [-0.63-0.62]	-0.13 [-0.63-0.62]	-0.13 [-0.63-0.62]	1.53	< .01	1.00
Speed of Processing	0.04 [-0.34-0.38]	0.04 [-0.35-0.42]	0.030 [-0.33-0.34]	0.54	< .01	1.00
Verbal Learning	0.16 [-0.61-0.71]	0.16 [-0.61-0.80]	0.16 [-0.50-0.71]	-0.56	< .01	1.00
Reasoning	0.19 [-0.52-0.66]	0.19 [-0.29-0.66]	0.19 [-0.52-0.67]	1.77	< .01	1.00
Attention	0.18 [-1.05-1.32]	0.18 [-1.04-1.33]	0.17 [-1.05-1.304]	0.24	< .01	1.00
Global Score	0.37 [-2.35-2.92]	0.38 [-2.40-3.05]	0.37 [-2.31-2.831]	0.35	< .01	1.00
Additional						
Imaqe Quality Rating	1.88 [1.83-1.97]	1.89 [1.86-1.96]	1.88 [1.82-1.97]	2.96	< .01	
Site				484.78	0.52	**3.00E-96**

Values represent the median [IQR] unless otherwise specified. Group-level differences between individuals included in the analysis (N = 678) and individuals not excluded due to non-availability of data (N = 1122) were assessed using the Wilcoxon rank-sum test for continuous variables and the Chi-squared test for binary variables. Effect sizes were estimated using η^2 for continuous variables and Cramér's V for binary variables. P-values were adjusted for multiple testing using Bonferroni correction, the entire table representing a family of tests. Significant P-values are highlighted in bold. Discrepancies in reported numbers may arise from incomplete or incorrectly entered data. Abbreviations: BDI-II, Beck Depression Inventory II; BMI, Body Mass Index; CHR-P, Clinical High Risk for Psychosis; CTQ, Childhood Trauma Questionnaire; GAF, Global Assessment of Functioning; GF, Global Functioning; NEO-FFI, NEO Five-Factor Inventory; PANSS, Positive and Negative Syndrome Scale; ROP, Recent-Onset Psychosis; ROD; WHOQOL-BREF, World Health Organization Quality of Life – BREF.

© 2025 Popovic D et al. *JAMA Psychiatry*.

eTable 6. Group-level Multiple Comparison Tests for Sociodemographic, Clinical and Blood Parameter Differences

	Groups	Lower Boundary	Estimate	Upper Boundary	P-value
Sociodemographic Data					
Age	ROP x CHR-P	22.69	60.51	98.34	**.060**
	ROP x ROD	-17.08	21.26	59.61	1.00
	CHR-P x ROD	-77.86	-39.25	-0.64	1.00
Education, y	ROP x CHR-P	-34.33	3.02	40.37	1.00
	ROP x ROD	-71.91	-33.97	3.97	1.00
	CHR-P x ROD	-75.19	-36.99	1.22	1.00
BMI	ROP x CHR-P	-26.57	10.82	48.21	1.00
	ROP x ROD	-44.48	-6.5	31.47	1.00
	CHR-P x ROD	-55.51	-17.32	20.87	1.00
Symptomatology					
BDI-II	ROP x CHR-P	-112.5	-76.55	-40.6	**1.66E-4**
	ROP x ROD	-93.29	-56.8	-20.31	.091
	CHR-P x ROD	-16.69	19.75	56.18	1.00
PANSS, Total	ROP x CHR-P	66.26	103.46	140.67	**1.42E-8**
	ROP x ROD	121.69	159.31	196.94	**0**
	CHR-P x ROD	17.84	55.85	93.86	.203
PANSS, Positive	ROP x CHR-P	109.22	146.27	183.31	**0**
	ROP x ROD	232.28	269.8	307.32	**0**
	CHR-P x ROD	85.69	123.53	161.38	**2.90E-12**
PANSS, Negative	ROP x CHR-P	5.58	42.76	79.94	1.00
	ROP x ROD	17.55	55.32	93.09	.212
	CHR-P x ROD	-25.43	12.56	50.54	1.00
PANSS. General	ROP x CHR-P	27.42	64.6	101.79	**.015**
	ROP x ROD	56.68	94.28	131.88	**9.65E-7**
	CHR-P x ROD	-8.31	29.68	67.66	1.00
Level of Functioning					

© 2025 Popovic D et al. *JAMA Psychiatry*.

GAF:S, Past month	ROP x CHR-P	-140.92	-103.35	-65.79	**2.27E-8**
	ROP x ROD	-173.58	-135.37	-97.17	**0**
	CHR-P x ROD	-70.38	-32.02	6.34	1.00
GAF:D/I, Past month	ROP x CHR-P	-121.54	-83.98	-46.42	**4.21E-5**
	ROP x ROD	-157.44	-119.24	-81.03	**4.12E-11**
	CHR-P x ROD	-73.62	-35.26	3.1	1.00
GF:S, Current	ROP x CHR-P	-97.7	-61.09	-24.48	**.030**
	ROP x ROD	-98.63	-61.45	-24.28	**.036**
	CHR-P x ROD	-37.7	-0.36	36.97	1.00
GF:R, Current	ROP x CHR-P	-90.78	-53.82	-16.86	.228
	ROP x ROD	-109.31	-71.77	-34.24	**.002**
	CHR-P x ROD	-55.65	-17.95	19.74	1.00
NEO-FFI					
Neuroticism	ROP x CHR-P	-108.32	-72.98	-37.63	**3.70E-4**
	ROP x ROD	-96.06	-60	-23.94	**.032**
	CHR-P x ROD	-22.68	12.98	48.63	1.00
Extraversion	ROP x CHR-P	36.99	72.36	107.7	**4.64E-4**
	ROP x ROD	-3	32.83	68.66	1.00
	CHR-P x ROD	-74.76	-39.52	-4.29	1.00
Openness	ROP x CHR-P	-28.57	7.00	42.57	1.00
	ROP x ROD	2.32	38.41	74.49	1.00
	CHR-P x ROD	-3.84	31.41	66.66	1.00
Agreeableness	ROP x CHR-P	-12.51	23.17	58.86	1.00
	ROP x ROD	-72.77	-36.56	-0.35	1.00
	CHR-P x ROD	-95.54	-59.73	-23.92	**.031**
Conscientiousness	ROP x CHR-P	19.64	55.41	91.18	.097
	ROP x ROD	-20.44	15.75	51.93	1.00
	CHR-P x ROD	-75.56	-39.66	-3.77	1.00
WHOQOL-BREF					
Physical	ROP x CHR-P	28.04	63.81	99.57	**.009**
	ROP x ROD	22.04	58.27	94.51	.055

© 2025 Popovic D et al. *JAMA Psychiatry*.

	CHR-P x ROD	-41.48	-5.53	30.42	1.00
Psychosocial	ROP x CHR-P	53.11	88.83	124.6	**1.30E-6**
	ROP x ROD	41.53	77.78	114	**1.36E-4**
	CHR-P x ROD	-47.13	-11.05	25.04	1.00
Social relationship	ROP x CHR-P	-16.56	19.01	54.59	1.00
	ROP x ROD	-20.73	15.2	51.13	1.00
	CHR-P x ROD	-39.57	-3.81	31.94	1.00
Environment	ROP x CHR-P	-20.22	15.23	50.69	1.00
	ROP x ROD	-34.5	1.38	37.26	1.00
	CHR-P x ROD	-49.74	-13.86	22.03	1.00
CTQ					
Emotional Abuse	ROP x CHR-P	-57.47	-22.41	12.64	1.00
	ROP x ROD	-18.29	17.65	53.6	1.00
	CHR-P x ROD	4.63	40.07	75.5	1.00
Physical Abuse	ROP x CHR-P	-33.45	-2.51	28.42	1.00
	ROP x ROD	-23.83	7.84	39.51	1.00
	CHR-P x ROD	-21.02	10.35	41.73	1.00
Sexual Abuse	ROP x CHR-P	-23.72	1.84	27.4	1.00
	ROP x ROD	-10.28	15.76	41.79	1.00
	CHR-P x ROD	-11.78	13.92	39.62	1.00
Emotional Neglect	ROP x CHR-P	-58.06	-23.02	12.01	1.00
	ROP x ROD	-40.44	-4.56	31.33	1.00
	CHR-P x ROD	-17.14	18.46	54.07	1.00
Physical Neglect	ROP x CHR-P	-30.69	3.96	38.61	1.00
	ROP x ROD	-13.59	21.76	57.12	1.00
	CHR-P x ROD	-17.27	17.81	52.88	1.00
Blood Parameter					
IFN-γ, pg/mL	ROP x CHR-P	-26.92	10.64	48.2	1.00
	ROP x ROD	-40.41	-2.33	35.76	1.00
	CHR-P x ROD	-51.31	-12.96	25.38	1.00

© 2025 Popovic D et al. *JAMA Psychiatry*.

IL-1RA, pg/mL	ROP x CHR-P	-47.67	-9.84	27.98	1.00
	ROP x ROD	-70.09	-31.74	6.6	1.00
	CHR-P x ROD	-60.52	-21.9	16.71	1.00
IL-4, pg/mL	ROP x CHR-P	-57.51	-19.86	17.78	1.00
	ROP x ROD	-46.55	-8.39	29.77	1.00
	CHR-P x ROD	-26.95	11.47	49.9	1.00
S100B, pg/mL	ROP x CHR-P	-33.29	4.5	42.29	1.00
	ROP x ROD	-32.39	5.93	44.25	1.00
	CHR-P x ROD	-37.15	1.43	40.02	1.00
IL-1β, pg/mL	ROP x CHR-P	-40.9	-3.23	34.43	1.00
	ROP x ROD	-17.28	20.91	59.1	1.00
	CHR-P x ROD	-14.31	24.15	62.6	1.00
IL-2, pg/mL	ROP x CHR-P	-30.4	7.31	45.02	1.00
	ROP x ROD	-22.63	15.6	53.84	1.00
	CHR-P x ROD	-30.2	8.29	46.79	1.00
IL-6, pg/mL	ROP x CHR-P	-43.58	-5.79	32.01	1.00
	ROP x ROD	-35.5	2.82	41.14	1.00
	CHR-P x ROD	-29.98	8.61	47.2	1.00
TNF-α, pg/mL	ROP x CHR-P	-30.63	7.19	45	1.00
	ROP x ROD	-36.07	2.27	40.61	1.00
	CHR-P x ROD	-43.52	-4.91	33.69	1.00
CRP, mg/L	ROP x CHR-P	-46.34	-8.52	29.31	1.00
	ROP x ROD	-43.25	-4.9	33.45	1.00
	CHR-P x ROD	-35	3.62	42.23	1.00
TGF-β, ng/mL	ROP x CHR-P	-34.21	3.61	41.43	1.00
	ROP x ROD	-60.16	-21.81	16.53	1.00
	CHR-P x ROD	-64.04	-25.43	13.19	1.00
BDNF, ng/mL	ROP x CHR-P	-40.88	-3.06	34.76	1.00
	ROP x ROD	-50.35	-12	26.35	1.00
	CHR-P x ROD	-47.55	-8.94	29.68	1.00

© 2025 Popovic D et al. *JAMA Psychiatry*.

Length of Storage, d	ROP x CHR-P	-36.2	0.92	38.04	1.00
	ROP x ROD	-57.51	-19.91	17.69	1.00
	CHR-P x ROD	-58.76	-20.83	17.09	1.00
MATRICS					
Social Cognition	ROP x CHR-P	-75.53	-38.87	-2.22	1.00
	ROP x ROD	-92.18	-54.94	-17.7	.192
	CHR-P x ROD	-53.46	-16.06	21.34	1.00
Working Memory	ROP x CHR-P	-102.25	-65.42	-28.6	**.010**
	ROP x ROD	-91.83	-54.35	-16.87	.241
	CHR-P x ROD	-26.57	11.07	48.71	1.00
Speed of Processing	ROP x CHR-P	-104.53	-67.96	-31.39	**.004**
	ROP x ROD	-147.28	-110.23	-73.18	**5.45E-10**
	CHR-P x ROD	-79.43	-42.27	-5.11	1.00
Verbal Learning	ROP x CHR-P	-90	-53.05	-16.11	.272
	ROP x ROD	-102.72	-65.25	-27.77	**.014**
	CHR-P x ROD	-49.78	-12.19	25.39	1
Reasoning	ROP x CHR-P	-92.81	-56.46	-20.11	.094
	ROP x ROD	-96.21	-59.58	-22.94	**.047**
	CHR-P x ROD	-39.64	-3.11	33.41	1.00
Attention	ROP x CHR-P	-74.62	-37.66	-0.7	1.00
	ROP x ROD	-92.45	-54.96	-17.47	.209
	CHR-P x ROD	-54.9	-17.3	20.3	1.00
Global Score	ROP x CHR-P	-97.13	-61.91	-26.69	**.012**
	ROP x ROD	-122.9	-87.19	-51.48	**2.50E-6**
	CHR-P x ROD	-60.99	-25.28	10.43	1
Medication					
Chlorpromazine-Eq	ROP x CHR-P	99.4	131.08	162.80	**0**
	ROP x ROD	127.04	158.21	189.40	**0**
	CHR-P x ROD	-5.11	27.13	59.36	1.00
Olanzapine-Eq	ROP x CHR-P	98.57	130.25	161.90	**0**
	ROP x ROD	126.38	157.54	188.70	**0**

© 2025 Popovic D et al. *JAMA Psychiatry*.

	CHR-P x ROD	-4.94	27.29	59.53	1.00
SSRI-Eq	ROP x CHR-P	-104.47	-72.15	-39.83	**4.41E-5**
	ROP x ROD	-109.27	-77.47	-45.66	**2.71E-6**
	CHR-P x ROD	-37.98	-5.32	27.35	1.00
Diazepam-Eq	ROP x CHR-P	8.91	37.51	66.11	.782
	ROP x ROD	3.04	31.18	59.32	1.00
	CHR-P x ROD	-35.34	-6.33	22.68	1.00
Additional Information					
Image Quality Rating	ROP x CHR-P	-29.8	8.01	45.81	1.00
	ROP x ROD	-29.68	8.6	46.87	1.00
	CHR-P x ROD	-38	0.59	39.18	1.00

Group-level differences between CHR-P, ROP and ROD individuals were assessed using Dunn's multiple comparison test. P-values were adjusted for multiple testing using Bonferroni correction, the entire table representing a family of tests. Significant P-values are highlighted in bold. Abbreviations: BDI-II, Beck Depression Inventory II; BDNF, Brain-Derived Neurotrophic Factor; BMI, Body Mass Index; CHR-P, Clinical High-Risk for Psychosis; CRP, C-Reactive Protein; CTQ, Childhood Trauma Questionnaire; GAF, Global Assessment of Functioning; GF, Global Functioning; IFN, Interferon; IL, Interleukin; NEO-FFI, NEO Five-Factor Inventory; PANSS, Positive and Negative Syndrome Scale; ROP, Recent-Onset Psychosis; ROD; s100B; SSRI, Selective Serotonin Reuptake Inhibitor; TGF, Transforming Growth Factor; TNF, Tumor Necrosis Factor; WHOQOL-BREF, WHO Quality of Life Short Version.

© 2025 Popovic D et al. *JAMA Psychiatry*.

eTable 7. Group-level Differences between Individuals from Discovery and Replication Sample

	All	Discovery	Replication	χ^2	η^2	P-value
N	678	453	225			
Sociodemographic Data						
Age, y	24 [20.9-28.85]	24.61 [21.19-29.34]	22.98 [19.97-27.46]	3.04	0.01	.128
Sex				0.39	0.02	1.00
Male sex, No. (%)	332 (48.97)	218 (48.12)	114 (50.67)			
Female sex, No. (%)	346 (51.03)	235 (51.88)	111 (49.33)			
BMI	22.53 [20.43-25.06]	22.57 [20.47-25.12]	22.49 [20.35-25.01]	0.31	< .01	1.00
Handedness (Right), No. (%)						
Education, y	15.88 (33.92)	14.92 (3.23)	17.79 (58.62)	4.38	0.03	**6.53E-04**
Tobacco Use, No. (%)	257 (37.91)	159 (35.1)	98 (43.56)	4.61	0.08	1.00
Symptomatology						
BDI-II	18 [5-30]	12 [3-28]	26 [15-35]	-7.90	0.09	**1.45E-13**
PANSS						
Total	55 [45-69]	51 [43-66]	60.5 [48-70]	-4.16	0.03	**.002**
Positive	11 [8-16]	11 [8-15.5]	12 [8.5-18]	-2.23	< .01	1.00
Negative	13 [9-18]	12 [9-17]	14 [10-21]	-3.12	0.01	.097
General	29 [24-36]	28 [23-34]	32 [25-38]	-3.62	0.02	**.016**
Blood parameters						
IFN-γ, pg/mL	1.59 [1.14-3.33]	1.59 [1.14-3.33]	2.68 [1.25-2.68]	-2.96	0.01	.166
IL-1RA, pg/mL	480.51 [366.94-657.19]	485.03 [356.75-662.88]	476.8 [383.86-654.68]	-0.55	< .01	1.00
IL-4, pg/mL	8.01 [3.1-11.73]	8.04 [3.1-8.36]	8.01 [3.83-13.59]	1.47	< .01	1.00
S100B, pg/mL	37.51 [12.1-78.9]	25.97 [10.28-54.84]	74.81 [49.4-86.92]	-10.79	0.17	**2.14E-25**
IL-1β, pg/mL	0.57 [0.48-1.46]	0.57 [0.48-0.73]	1.46 [0.2-1.93]	-5.35	0.04	**4.69E-06**
IL-2, pg/mL	0.72 [0.3-1.43]	0.43 [0.3-0.97]	1.33 [0.72-2.03]	-8.72	0.11	**1.50E-16**
IL-6, pg/mL	0.53 [0.22-1.03]	0.53 [0.25-0.97]	0.65 [0.14-1.25]	-1.04	< .01	1.00
TNF-α, pg/mL	1.52 [0.71-2.43]	1.34 [0.46-2.24]	2.03 [1.04-2.76]	-5.16	0.04	**1.35E-05**
CRP, mg/L	0.58 [0.22-1.46]	0.59 [0.20-1.50]	0.56 [0.24-1.33]	0.33	< .01	1.00
TGF-β, ng/mL	149.62 [122.00-226.40]	156.45 [121.23-621.61]	148.00 [123.05-170.39]	3.97	0.02	**.004**

© 2025 Popovic D et al. *JAMA Psychiatry*.

BDNF, ng/mL	22.73 [18.06-2727]	22.17 [17.12-27.84]	23.20 [19.06-26.37]	-1.14	< .01	1.00
Length of Storage, d	1385 [1062-1632]	1558 [1369-1705]	854 [706.25-1054.75]	19.88	0.58	**3.51E-86**
Level of Functioning						
GAF:S, past month	55 [45-75]	60 [50-85]	47 [39.5-55]	10.16	0.15	**1.55E-22**
GAF:D/I, past month	55 [41.75-80]	60 [49-83]	45 [40-55]	9.79	0.14	**6.44E-21**
GF:S, current	7 [6-8]	7 [6-8]	6 [5-7]	8.41	0.10	**2.19E-15**
GF:R, current	6.5 [5-8]	7 [5-8]	5 [4-7]	9.3	0.13	**7.62E-19**
NEO-FFI						
Neuroticism	38 [29.75-46]	35 [28-44]	42 [37-48]	-6.54	0.06	**3.41E-09**
Extraversion	36 [31-43]	38 [31-44]	33 [28-39]	5.42	0.04	**3.25E-06**
Openness	41 [37-45]	41 [36-45]	41 [37-45]	-0.15	< .01	1.00
Agreeableness	43 [38-48]	44 [39-48]	41 [37-46]	4.36	0.03	**7.08E-04**
Conscientiousness	41 [36-46]	42 [36.75-48]	39 [33-42.25]	5.26	0.04	**7.63E-06**
WHOQOL-BREF						
Physical	25 [21-30]	26 [22-31]	23 [19-26]	6.97	0.07	**1.70E-10**
Psychosocial	18 [14-23]	20 [15-24]	15 [13-19]	7.59	0.09	**1.68E-12**
Social Relationships	10 [8-12]	11 [9-12]	9 [7-11]	4.92	0.04	**4.64E-05**
Environment	30 [27-34]	31 [27-34]	29 [26-33]	3.68	0.02	**.013**
CTQ						
Emotional Abuse	8 [6-11]	7 [5-10]	9 [6-13]	-3.93	0.02	**.005**
Physical Abuse	5 [5-6]	5 [5-6]	5 [5-7]	-2.52	< .01	.632
Sexual Abuse	5 [5-5]	5 [5-5]	5 [5-5]	-1.11	< .01	1.00
Emotional Neglect	10 [7-14]	10 [6-13]	10 [8-14]	-2.08	< .01	1.00
Physical Neglect	6 [5-8]	6 [5-8]	6 [5-8]	-1.56	< .01	1.00
MATRICS battery						
Social Cognition	-0.01 [-0.42-0.80]	-0.01 [-0.42-0.8]	-0.01 [-0.42-0.8]	0.75	< .01	1.00
Working Memory	-0.09 [-0.6-0.66]	0.16 [-0.6-0.66]	-0.35 [-0.85-0.41]	3.85	0.02	**.006**
Speed of Processing	0.05 [-0.31-0.37]	0.11 [-0.21-0.44]	-0.06 [-0.46-0.24]	4.93	0.04	**4.48E-05**
Verbal Learning	0.14 [-0.55-0.71]	0.25 [-0.44-0.71]	0.02 [-0.67-0.48]	2.85	0.01	.238
Reasoning	0.25 [-0.51-0.76]	0.25 [-0.51-0.76]	-0 [-0.51-0.51]	2.2	< .01	1.00
Attention	0.2 [-0.99-1.30]	0.38 [-0.73-1.42]	-0.2 [-1.44-0.85]	4.22	0.03	**.001**

© 2025 Popovic D et al. *JAMA Psychiatry*.

Global Score	0.54 [-2.24-2.96]	1.05 [-1.8-3.44]	-0.65 [-3.36-1.94]	4.74	0.03	**1.15E-04**
Medication						
Chlorpromazine-Eq (mg)	0 [0-1170]	0 [0-288]	318.75 [0-4380]	-5.97	0.05	**1.30E-07**
Olanzapine-Eq (mg)	0 [0-42.81]	0 [0-10.42]	11.63 [0-149.2]	-5.95	0.05	**1.48E-07**
SSRI-Eq (mg)	0 [0-730.96]	0 [0-407.95]	92 [0-2192.53]	-4.43	0.03	**5.12E-04**
Diazepam-Eq (mg)	0 [0-0]	0 [0-0]	0 [0-70]	-3.29	0.02	.053
Additional Information						
Image Quality Rating	1.88 [1.82-1.97]	1.88 [1.82-1.99]	1.87 [1.82-1.94]	0.98	< .01	1.00
Site				76.49	< .01	**3.86E-12**

Values represent the median [IQR] unless otherwise specified. Group-level differences between individuals from the discovery (N = 453) and the replication sample (N = 225) included in the analysis were assessed using the Wilcoxon rank-sum test for continuous variables and the Chi-squared test for binary variables. Effect sizes were estimated using η^2 for continuous variables and Cramér's V for binary variables. P-values were adjusted for multiple testing using Bonferroni correction, the entire table representing a family of tests. Significant P-values are highlighted in bold. Discrepancies in reported numbers may arise from incomplete or incorrectly entered data. Abbreviations: BDI-II, Beck Depression Inventory II; BMI, Body Mass Index; CHR-P, Clinical High-Risk for Psychosis; CTQ, Childhood Trauma Questionnaire; GAF, Global Assessment of Functioning; GF, Global Functioning; NEO-FFI, NEO Five-Factor Inventory; PANSS, Positive and Negative Syndrome Scale; ROP, Recent-Onset Psychosis; ROD; WHOQOL-BREF, World Health Organization Quality of Life – BREF.

© 2025 Popovic D et al. *JAMA Psychiatry*.

eTable 8. Neurocognitive Test Battery

Cognitive test	Description	Measure of interest
	**Social Cognition**	
Diagnostic Analysis of Nonverbal Accuracy-2[24]	Participants are presented with 24 faces on a tablet showing 4 different emotions; happy, neutral, angry, sad and must decide which emotion is represented.	Number of correct responses
	**Speed of Processing**	
1. Trail Making Test (TMT): Part A[26]<br>2. Verbal Fluency: semantic[27]<br>3. Digit symbol coding task[25]	1. Participants must combine numbers in ascendent order (paper-pencil)<br>2. Participants had 1 minute to produce as many words as possible from the semantic category *animals*.<br>3. Participants were presented with 9 symbols each corresponding to a number from 1-9 on the top of a sheet of paper. Then, they had to write the corresponding number under as many symbols as possible in 1 minute on the same sheet of paper. (paper-pencil)	1. Time of execution<br>2. Correct words<br>3. Number of correctly matched symbols
	**Working Memory**	
Wechsler Memory Scale, 3rd ed., spatial span subtest[25]	Participants must repeat sequences of numbers with increasing difficulties (one number added in each sequence) first forward then backward.	Sum of number of correct trials
	**Verbal Learning**	
Rey Auditory Verbal Learning Test (RAVLT[21]) and (for Turku site) harmonized HVLT-R[22]	Participants must immediately recall as many words as possible from a list of 12 words that is audio-played to them.	Sum of correctly recalled words
	**Reasoning**	
Wechsler Adult Intelligence Scale, 4th ed., Matrix Reasoning[25]	Participants are presented with a matrix showing a sequence of abstract pictures. The participants must decide which picture of a number of possible options would complete the sequence best.	Sum of correct responses
	**Attention**	
Continuous Performance Task – Identical Pairs (CPT-IP)[28]	Participants were presented with 300 four-digit numbers on a tablet-screen with a rate of one per second and had to click as fast as possible on a computer-mouse in case of identical repeating numbers.	Difference between standardized z-scores of correct and false alarms
	**Global Cognition**	
Composite across all cognitive measures included above (average z-score)	The scores from all included domains were standardized to z-scores.	Sum of all standardized z-scores

Table adapted from Haas et al[15] and previously published in Penzel et al[23] and reprinted with permission.

© 2025 Popovic D et al. *JAMA Psychiatry*.

eTable 9. Weight Vectors of the Blood Parameter Signatures of LV1-LV4

	LV1	LV2	LV3	LV4
CHR-P	-0.106	0	-0.458	0
HC	0	0	0	-0.262
ROD	0	0	0	0.255
ROP	0.118	0	0.449	0
Age	0.969	0	0.350	0
Male	0	0.665	0	0
Female	0	-0.665	0	0
BMI	0.176	0	0.371	0
IFN-γ	0.012	0	0	0
IL-1RA	0	0	0	0.108
IL-4	0	0	0	0.381
S100B	0	0	0	0.264
IL-1β	0	0	0	0.439
IL-2	0	0	0	0.564
IL-6	0.018	0	0.148	0
TNF-α	0.060	0	0.428	0
CRP	0.021	0	-0.352	0
TGF-β	0	0.044	0	0
BDNF	0	0	0	0.360
IQR	0	0.339	0	0

Depicted are the dimensionless weights of each variable included in the phenotypic matrix for LV1-LV4. Abbreviations: BDNF, Brain-Derived Neurotrophic Factor; BMI, Body Mass Index; CHR-P, Clinical High-Risk for Psychosis; CRP, C-Reactive Protein; HC, Healthy Control Individuals; IFN, Interferon; IL, Interleukin; IL-1RA, Interleukin-1 Receptor Antagonist Protein; IQR, Image Quality Rating; ROD, Recent-Onset Depression; ROP, Recent-Onset Psychosis; S100B, S100 Calcium-Binding Protein B; TGF, Transforming Growth Factor; TNF, Tumor Necrosis Factor.

eTable 10. Number Of Features in the Brain and Blood Parameter Weight Vectors Before and After Bootstrapping

	Before Bootstrapping	After Bootstrapping
LV1	0	0
Brain	2081	2014
Blood parameter	8	8
LV2	0	0
Brain	3782	3517
Blood parameter	4	4
LV3	0	0
Brain	9538	9174
Blood parameter	7	7
LV4	0	0
Brain	8380	8128
Blood parameter	8	8

To evaluate the stability of feature weights within a significant LV, the bootstrap ratio for each feature across 500 bootstrap samples was calculated[41]. Features with a bootstrap ratio > |2| remained in the LV.

eTable 11. Comparison of Latent Scores between Discovery and Replication Sample

	All	Discovery	Replication	z	η^2	P-value
N	678	453	225			
LV1						
Blood parameter	-0.18 [-0.82-0.65]	-0.07 [-0.74-0.73]	-0.33 [-0.93-0.41]	2.4	< .01	.130
Brain	0.3 [-15.02-14.23]	-1.07 [-16.1-12.97]	2.15 [-13.35-16.26]	-1.27	< .01	1.00
LV2						
Blood parameter	-0.41 [-1.43-1.26]	-0.7 [-1.43-1.3]	1.04 [-1.43-1.2]	0.84	< .01	1.00
Brain	-1.15 [-12.83-11.02]	-0.64 [-12.82-10.31]	-2.76 [-13.03-11.92]	0.63	< .01	1.00
LV3						
Blood parameter	0.66 [0.08-0.89]	0.65 [0.06-0.9]	0.67 [0.17-0.88]	-0.33	< .01	1.00
Brain	0.11 [-9.37-9.29]	0.81 [-9.32-9.96]	-0.67 [-9.38-7.42]	1.17	< .01	1.00
LV4						
Blood parameter	-0.2 [-0.83-0.51]	-0.52 [-1.04-0.32]	0.28 (0.86)	-7.89	0.09	**2.49E-14**
Brain	0.3 [-25.17-24.79]	-4.06 [-29.91-20.78]	8.32 (36.7)	-4.27	0.03	**1.56E-4**

Values represent the median [IQR]. Group-level differences were assessed using the Wilcoxon rank-sum test. Effect size was estimated using η^2. P-values were adjusted for multiple testing using Bonferroni correction, the entire table representing a family of tests. Significant P-values are highlighted in bold.

eTable 12. Comparison of Latent Scores between CHR-P Subgroups

	All	COGDIS	COGDIS + UHR	UHR	χ^2	η^2	P-value
N	170	40	56	74			
LV1							
Blood parameter	-0.78 [-1.16--0.09]	-0.45 [-1.02-0.25]	-0.86 [-1.24--0.1]	-0.82 [-1.16--0.24]	3.23	< .01	1.00
Brain	-5.72 [-19.27-9.57]	-4.22 [-17.17-17.75]	-5.22 [-19.63-9.88]	-8.31 [-19.27-4.28]	1.34	< .01	1.00
LV2							
Blood parameter	-0.84 [-1.44-1.21]	-1.24 [-1.42-1.24]	0.15 [-1.44-1.17]	-0.65 [-1.49-1.35]	0.45	< .01	1.00
Brain	-3.01 [-13.93-9.96]	-4.37 [-19.94-4.64]	-3.84 [-17.42-9.97]	-1.42 [-10.79-12.67]	4.38	0.01	.897
LV3							
Blood parameter	0.65 [0.12-0.87]	0.63 [0.13-0.86]	0.59 [0.25-0.9]	0.67 [0.01-0.86]	0.30	< .01	1.00
Brain	1.73 [-8.41-11.33]	-1.35 [-12.32-7.73]	1.81 [-6.29-10.28]	4.4 [-6.9-12.05]	2.84	< .01	1.00
LV4							
Blood parameter	-0.26 [-0.64-0.41]	-0.27 [-0.71-0.48]	-0.28 [-0.53-0.38]	-0.2 [-0.66-0.35]	0.15	< .01	1.00
Brain	-3.85 [-32.65-21.17]	-3.56 [-31.59-20.01]	0.3 [-23.31-27.11]	-10 [-38.88-21.17]	2.14	< .01	1.00

Values represent the median [IQR]. The CHR-P state was defined by either 1) cognitive disturbances (COGDIS) criteria assessed using the Schizophrenia Proneness Instrument[3,4] and/or 2) ultra-high-risk (UHR) criteria for psychosis based on the Structured Interview for Prodromal Syndromes[5]. Group-level differences were assessed using the Kruskal-Wallis test. Effect size was estimated using η2. P-values were adjusted for multiple testing using Bonferroni correction, the entire table representing a family of tests. Abbreviations: COGDIS, Cognitive Disturbances; UHR, Ultra High-Risk.

eTable 13. Broad Atlas Readouts from LV1-LV4 for Neuroanatomical Brain Regions

See additional Excel file.

eTable 14. Detailed Atlas Readouts from LV1-LV4 for Neuroanatomical Brain Regions

See additional Excel file.

eTable 15. Atlas Readouts from LV1-LV4 for Large-scale Brain Networks

Weighted voxels	Total voxels	Weighted voxels ratio	Network	Median weights	Mean weights
		**LV1**			
		Positive weights			
0	151	5761	2.62%	Visual	-0.004
0	434	9709	4.47%	Somatomotor	-0.006
0	315	7965	3.95%	Dorsal Attention	-0.006
0	674	7495	8.99%	Ventral Attention	-0.005
0	663	6008	11.04%	Salience	-0.007
0	159	8118	1.96%	Limbic	-0.004
0	1008	16271	6.20%	Control	-0.006
0	2088	20792	10.04%	Default	-0.006
		Negative weights			
8	1616	0.5%	Visual	-0.018	-0.016
46	2735	1.7%	Somatomotor	-0.017	-0.018
43	2241	1.9%	Dorsal Attention	-0.007	-0.010
87	2170	4.0%	Ventral Attention	-0.013	-0.016
113	1694	6.7%	Salience	-0.015	-0.018
0	2266	0.0%	Limbic	0.000	0.000
131	4521	2.9%	Control	-0.014	-0.017
476	5884	8.1%	Default	-0.014	-0.017
		**LV2**			
		Positive weights			
43	1616	2.7%	Visual	0.009	0.010
19	2735	0.7%	Somatomotor	0.007	0.007
44	2241	2.0%	Dorsal Attention	0.007	0.011
33	2170	1.5%	Ventral Attention	0.006	0.009
4	1694	0.2%	Salience	0.003	0.003
182	2266	8.0%	Limbic	0.006	0.007
37	4521	0.8%	Control	0.012	0.014
157	5884	2.7%	Default	0.016	0.017
		Negative weights			
41	1616	2.5%	Visual	-0.008	-0.008
160	2735	5.9%	Somatomotor	-0.009	-0.012
109	2241	4.9%	Dorsal Attention	-0.011	-0.013
26	2170	1.2%	Ventral Attention	-0.006	-0.006
35	1694	2.1%	Salience	-0.005	-0.005
23	2266	1.0%	Limbic	-0.008	-0.013

307	4521	6.8%	Control	-0.010	-0.012
268	5884	4.6%	Default	-0.006	-0.008
			**LV3**		
			Positive weights		
297	1616	18.4%	Visual	0.008	0.010
310	2735	11.3%	Somatomotor	0.005	0.006
325	2241	14.5%	Dorsal Attention	0.008	0.009
84	2170	3.9%	Ventral Attention	0.004	0.005
93	1694	5.5%	Salience	0.004	0.005
92	2266	4.1%	Limbic	0.004	0.005
435	4521	9.6%	Control	0.006	0.007
623	5884	10.6%	Default	0.006	0.007
			Negative weights		
41	1616	2.5%	Visual	-0.004	-0.005
271	2735	9.9%	Somatomotor	-0.007	-0.010
191	2241	8.5%	Dorsal Attention	-0.006	-0.007
180	2170	8.3%	Ventral Attention	-0.005	-0.007
95	1694	5.6%	Salience	-0.003	-0.005
115	2266	5.1%	Limbic	-0.004	-0.007
560	4521	12.4%	Control	-0.007	-0.008
446	5884	7.6%	Default	-0.005	-0.006
			**LV4**		
			Positive weights		
37	1616	2.3%	Visual	0.006	0.006
2	2735	0.1%	Somatomotor	0.005	0.005
0	2735	0.0%	Dorsal Attention	0.000	0.000
14	2170	0.6%	Ventral Attention	0.008	0.008
21	1694	1.2%	Salience	0.006	0.008
0	2266	0.0%	Limbic	0.000	0.000
10	4521	0.2%	Control	0.007	0.006
9	5884	0.2%	Default	0.002	0.005
			Negative weights		
59	1616	3.7%	Visual	-0.005	-0.006
195	2735	7.1%	Somatomotor	-0.004	-0.005
194	2241	8.7%	Dorsal Attention	-0.005	-0.006
221	2170	10.2%	Ventral Attention	-0.005	-0.007
186	1694	11.0%	Salience	-0.005	-0.005
1142	2266	50.4%	Limbic	-0.010	-0.011
547	4521	12.1%	Control	-0.005	-0.006

1343	5884	22.8%	Default	-0.007	-0.010

Depicted is the percentage of positively and negatively weighted voxels (weighted voxels ratio) in 8 large-scale brain networks, derived from an adapted, network solution of the Yeo and the Buckner atlases, for LV1-4. Additionally, for each LV, weighted voxels, total voxels, median and mean weights of the positively and negatively weighted voxels belonging to each network are provided.

eTable 16. Correlation Coefficient between Latent Scores in Discovery and Replication Sample

	**Discovery (N = 453)**		**Replication (N = 225)**	
	Rho	P-value	Rho	P-value
LV1	0.60	< .001	0.65	< .001
LV2	0.56	< .001	0.48	< .001
LV3	0.27	< .001	0.19	0.005
LV4	0.22	< .001	0.21	0.001

The correlation coefficient between the latent scores, i.e., the optimization criterion for the SPLS analysis, for each LV was assessed using spearman correlation. *Abbreviations:* LV, Latent Variable.

eTable 17. SVM-C Performance Metrics

See additional Excel file.

eTable 18. Number of High- and Low-Scorers per SVM-C Prediction Model

Model	Total Sample Available	High-Scorer	Low-Scorer	Intermediate
LV3				
Medication	560	49 (42.24%)	67 (57.76%)	444
Psychosocial	607	48 (42.86%)	64 (57.14%)	495
Neurocognition	589	45 (42.45%)	61 (57.55%)	483
LV4				
Medication	560	55 (47.41%)	61 (52.59%)	444
Psychosocial	607	51 (48.57%)	54 (51.43%)	502
Neurocognition	589	46 (45.54%)	55 (54.46%)	488

Only individuals with complete data, whose brain and blood parameter latent scores fell within the top or bottom quartile of the latent score distribution, were included in the SVM-C analyses. This selection resulted in varying sample sizes across the prediction models. Individuals with complete data but latent scores which did not fall within the top or bottom quartile in both domains were assigned to the "intermediate" group and not included in the analysis.

eTable 19. Comparison of Psychosocial Predictors between LV3 Low- and High-Scorers

	All	Low-Scorer	High-Scorer	z	P-value
N	112	64	48		
CTQ					
Emotional Abuse	8 [6-12]	9 [6-12]	7 [6-11.75]	0.90	1.00
Physical Abuse	5 [5-7]	5 [5-7]	5 [5-6]	1.35	1.00
Sexual Abuse	5 [5-5]	5 [5-6]	5 [5-5]	1.53	1.00
Emotional Neglect	10 [7-14]	10 [7-14]	10 [7-15]	0.20	1.00
Physical Neglect	6 [5-8.5]	6 [5-9]	6 [5-8]	0.34	1.00
Denial	0 [0-0]	0 [0-1]	0 [0-0]	0.74	1.00
PAS					
Childhood					
Sociability and Withdrawal	1 [0-2]	0 [0-2]	1 [0-2]	-1.63	1.00
Peer Relationships	2 [0-2]	1 [0-2]	2 [0-2]	-0.08	1.00
Scholastic Performance	2 [1-3]	2 [1-3]	2 [0.5-2]	0.34	1.00
Adaptation To School	0 [0-1]	0 [0-1]	0 [0-1.5]	0.12	1.00
Early Adolescence					
Sociability and Withdrawal	1 [0-2]	1 [0-2]	1 [0-2]	-0.40	1.00
Peer Relationships	2 [1-2]	2 [1-2]	2 [1-2]	0.12	1.00
Scholastic Performance	2 [1.5-3]	2.5 [2-3]	2 [1-3]	1.50	1.00
Adaptation To School	1 [0-2]	1 [0-2]	1 [0-2]	0.36	1.00
Social-sexual Aspects	1 [0-2]	1 [0-2]	1 [0-2]	-0.46	1.00
Late Adolescence					
Sociability and Withdrawal	1 [0-2]	1 [0-2]	1 [0-2]	-0.96	1.00
Peer Relationships	2 [0-2]	1 [0-2]	2 [0.25-2]	-0.63	1.00
Scholastic Performance	2 [1-3]	2 [1-3]	2 [1.25-3]	-0.41	1.00
Adaptation To School	1 [0-2]	0.5 [0-1.5]	1 [0-2]	-1.24	1.00
Social-sexual Aspects	0 [0-2]	0 [0-2]	1 [0-2]	-1.43	1.00
GAF					
S: Highest Lifetime	83 [75-90]	81 [73-88.5]	83.5 [79-90]	-1.11	1.00

S: Highest Past Year	71 [61-85]	70 [60-80]	75 [62.5-85]	-1.76	1.00
S: Highest Past Month	51 [45-77]	53 [46-70]	51 [45-80.5]	0.14	1.00
D/I: Highest Lifetime	82.5 [79.5-85]	81 [75-85]	85 [80-85]	-1.34	1.00
D/I: Highest Past Year	75 [61-82]	71 [61-81]	75 [65-85]	-1.49	1.00
D/I: Highest Past Month	52 [45-80]	55 [46-72]	50.5 [42-80.5]	0.2	1.00
GF					
Social: Current	7 [6-8]	7 [6-8]	6 [6-8]	0.85	1.00
Social: Lowest Past Year	6 [5-8]	6 [5-7.5]	6 [5-8]	-0.26	1.00
Social: Highest Past Year	8 [7-8]	8 [7-8]	8 [6.5-8]	0.31	1.00
Social Highest Lifetime	8 [8-9]	8 [8-9]	8 [7.5-9]	0.75	1.00
Role: Current	6 [5-8]	6 [5-8]	7 [5-8]	-0.27	1.00
Role: Lowest Past Year	6 [5-7]	6 [5-7]	6 [5-7]	-0.62	1.00
Role: Highest Past Year	8 [7-8]	7.5 [7-8]	8 [7-8]	-0.32	1.00
Role: Highest Lifetime	8 [8-9]	8 [8-9]	8 [8-9]	0.01	1.00
NEO-FFI					
Neuroticism	41 [32-46]	42 [32.25-47.5]	38 [29.5-43]	2.02	1.00
Extraversion	37 [30-42]	38 [31-42]	35 [30-42.75]	0.61	1.00
Openness	40 [37-44]	39.5 [37-43]	40 [37-45.75]	-0.89	1.00
Agreeableness	44 [36.5-47]	44 [38-47]	42 [35.25-47]	0.71	1.00
Conscientiousness	42 [37-46]	42 [38-46]	40 [36.25-46]	1.2	1.00
WHOQOL-BREF					
Environment	30 [26-34]	29 [24-33]	31 [28-34]	-2.51	.522
Physical	24 [20-30]	23 [20-28.75]	26 [23-31]	-1.86	1.00
Psychosocial	17 [14-23]	17 [13-23]	18 [15-24]	-1.32	1.00
Social relationship	10 [7.5-12]	10 [7-11]	11 [8-13]	-1.59	1.00

Values represent the median [IQR]. Group-level differences were assessed using the Mann-Whitney U-test. P-values were adjusted for multiple testing using Bonferroni correction, the entire table representing a family of tests. Significant P-values are highlighted in bold. Abbreviations: CTQ, Childhood Trauma Questionnaire; GAF, Global Assessment of Functioning (S, Symptomatology; D/I, Disability/Impairment); GF, Global Functioning; NEO-FFI, NEO Five-Factor Inventory; PAS, Premorbid Adjustment Scale; WHOQOL-BREF, WHO Quality of Life Short Version.

eTable 20. Comparison of Neurocognitive Predictors between LV3 Low- and High-Scorers

	All	Low-Scorer	High-Scorer	*z*	P-value
N	106	61	45		
Social Cognition	-0.01 [-0.42-0.79]	-0.01 [-0.52-0.39]	-0.01 [-0.42-0.79]	-0.78	1.00
Working Memory	-0.13 [-0.88-0.87]	-0.38 [-0.88-0.18]	0.37 [-0.45-1.18]	-3.4	**.005**
Speed of Processing	-0.02 [-0.37-0.34]	-0.11 [-0.41-0.39]	0.04 [-0.17-0.29]	-1.37	1.00
Verbal Learning	0.14 [-0.53-0.7]	0.03 [-0.56-0.48]	0.37 [-0.19-1.04]	-2.1	.253
Reasoning	0.17 [-0.54-0.65]	-0.06 [-0.77-0.41]	0.41 [0.12-0.88]	-3.78	**.001**
Attention	0.21 [-1.24-1.13]	0.11 [-1.06-1.12]	0.21 [-1.39-1.22]	0.24	1.00
Global Score	0.01 [-2.44-2.6]	-0.61 [-2.74-2.07]	0.84 [-2.07-3.22]	-2.15	.219

Values represent the median [IQR]. Group-level differences were assessed using the Mann-Whitney U-test. P-values were adjusted for multiple testing using Bonferroni correction, the entire table representing a family of tests. Significant P-values are highlighted in bold.

eTable 21. Comparison of Medication Predictors between LV3 Low- and High-Scorers

	All	Low-Scorer	High-Scorer	*z*	P-value
N	116	67	49		
Diazepam-Eq	0 [0-21]	0 [0-31.75]	0 [0-17.5]	-0.07	1.00
Chlorpromazine-Eq	0 [0-1300]	0 [0-1320]	0 [0-760]	0.29	1.00
Olanzapine	0 [0-43.55]	0 [0-44]	0 [0-25.4]	0.29	1.00
SSRI-Eq	0 [0-991.67]	0 [0-1170.97]	0 [0-372.28]	1.03	1.00

Values represent the median [IQR]. Group-level differences were assessed using the Mann-Whitney U-test. P-values were adjusted for multiple testing using Bonferroni correction, the entire table representing a family of tests. Abbreviations: Eq, Equivalents; SSRI, Selective Serotonin Reuptake Inhibitors.

eTable 22. Comparison of Psychosocial Predictors between LV4 Low- and High-Scorers

	All	Low-Scorer	High-Scorer	*z*	P-value
N	105	54	51		
CTQ					
Emotional Abuse	7 [5-10]	6 [5-8.25]	9 [6-11]	-3.06	.096
Physical Abuse	5 [5-6]	5 [5-6]	5 [5-7]	-1.83	1.00
Sexual Abuse	5 [5-5]	5 [5-5]	5 [5-5]	-0.80	1.00
Emotional Neglect	8.5 [5.5-12.5]	7 [5-11]	9 [6.25-13]	-1.61	1.00
Physical Neglect	6 [5-8]	5 [5-7]	6 [5-9]	-2.32	.863
Denial	0 [0-1]	1 [0-2]	0 [0-1]	2.35	.799
PAS					
Childhood					
Sociability and Withdrawal	1 [0-2]	0 [0-1]	1 [0-2]	-1.43	1.00
Peer Relationships	1 [0-2]	1 [0-2]	2 [0-2]	-1.72	1.00
Scholastic Performance	2 [0-2]	1 [0-2]	2 [1-3]	-3.36	**0.034**
Adaptation To School	0 [0-1]	0 [0-1]	0 [0-1.75]	-1.75	1.00
Early Adolescence					
Sociability and Withdrawal	1 [0-2]	0.5 [0-2]	1 [0-3]	-2.13	1.00
Peer Relationships	2 [0-2]	1 [0-2]	2 [1-2]	-2.07	1.00
Scholastic Performance	2 [0.75-3]	1 [0-2]	3 [2-4]	-4.43	**3.99E-4**
Adaptation To School	1 [0-1.25]	0 [0-1]	1 [0-2]	-2.22	1.00
Social-sexual Aspects	1 [0-2.25]	1 [0-2]	1 [0-3]	-1.79	1.00
Late Adolescence					
Sociability and Withdrawal	1 [0-2]	0 [0-1]	2 [0-2]	-2.68	.319
Peer Relationships	1 [0-2]	1 [0-2]	2 [0-2]	-1.75	1.00
Scholastic Performance	2 [1-3]	1.5 [0-2]	2 [2-3]	-3.04	.102
Adaptation To School	1 [0-2]	0 [0-1]	1 [0-2]	-3.25	**.049**
Social-sexual Aspects	0 [0-2]	0 [0-1]	0.5 [0-2]	-1.2	1.00
GAF					
S: Highest Lifetime	85 [78-90]	90 [85-91]	80 [71.5-85]	4.68	**1.21E-4**

S: Highest Past Year	80 [65-88]	85 [80-91]	65 [60-76.5]	5.7	**5.08E-7**
S: Highest Past Month	80 [65-85]	85 [80-90]	70 [61-80]	5.53	**1.82E-5**
D/I: Highest Lifetime	65 [50-85]	85 [65-90]	55 [44.25-65]	4.86	**5.83E-6**
D/I: Highest Past Year	85 [78-90]	90 [85-91]	80 [71.5-85]	5.06	**1.21E-4**
D/I: Highest Past Month	80 [65-88]	85 [80-91]	65 [60-76.5]	5.27	**5.08E-7**
GF					
Social: Current	7 [6-9]	8 [7-9]	6 [5-7]	5.26	**6.31E-6**
Social: Lowest Past Year	7 [5-8]	8 [7-9]	6 [5-7]	5.26	**6.32E-6**
Social: Highest Past Year	8 [7-9]	9 [8-9]	7 [7-8]	4.7	**1.10E-4**
Social Highest Lifetime	8 [8-9]	9 [8-9]	8 [8-8]	4.05	**.002**
Role: Current	7 [5-8]	8 [7-9]	6 [4-7]	5.44	**2.35E-6**
Role: Lowest Past Year	7 [5-8]	8 [7-9]	5 [4-7]	5.55	**1.21E-6**
Role: Highest Past Year	8 [7-9]	9 [8-9]	7 [7-8]	5.11	**1.39E-5**
Role: Highest Lifetime	8 [8-9]	9 [8-9]	8 [8-8]	4.19	**.001**
NEO-FFI					
Neuroticism	34 [26-41.75]	31 [20-36]	40.5 [30-46]	-4.25	**9.24E-4**
Extraversion	38 [29-46]	43 [31-47]	34.5 [28-44]	2.03	1.00
Openness	41 [36-46]	41.5 [37-46]	40 [35-45.25]	1.29	1.00
Agreeableness	45 [40.75-49]	47 [41-50]	43 [39-48]	2.09	1.00
Conscientiousness	42 [35.25-49]	44 [38.75-50.25]	41 [31-48]	1.85	1.00
WHOQOL-BREF					
Environment	32 [27-34]	32.5 [29-36]	31 [27-33]	1.68	1.00
Physical	27 [22-31]	30 [25-32]	24 [19.75-28.25]	3.46	**.023**
Psychosocial	21 [15-25]	24 [20.75-26]	17 [13-21.5]	4.31	**6.97E-4**
Social relationship	11 [8-12]	11 [10-13]	10 [7-11]	2.59	.412

Values represent the median [IQR]. Group-level differences were assessed using the Mann-Whitney U-test. P-values were adjusted for multiple testing using Bonferroni correction, the entire table representing a family of tests. Significant P-values are highlighted in bold. Abbreviations: CTQ, Childhood Trauma Questionnaire; GAF, Global Assessment of Functioning (S, Symptomatology; D/I, Disability/Impairment); GF, Global Functioning; NEO-FFI, NEO Five-Factor Inventory; PAS, Premorbid Adjustment Scale; WHOQOL-BREF, WHO Quality of Life Short Version.

eTable 23. Comparison of Neurocognitive Predictors between LV4 Low- and High-Scorers

	All	Low-Scorer	High-Scorer	*z*	P-value
N	101	55	46		
Social Cognition, mean (SD)	0.39 [-0.42-0.79]	0.39 [-0.01-0.79]	-0.01 [-0.82-0.39]	2.43	.105
Working Memory, mean (SD)	-0.13 [-0.38-0.62]	0.12 [-0.38-1.06]	-0.13 [-0.63-0.37]	2.22	.186
Speed of Processing, mean (SD)	0.12 [-0.17-0.44]	0.24 [-0.11-0.6]	0.07 [-0.24-0.31]	1.68	.649
Verbal Learning, mean (SD)	0.14 [-0.42-0.59]	0.14 [-0.12-0.59]	-0.08 [-0.75-0.59]	1.91	.394
Reasoning, mean (SD)	0.17 [-0.54-0.65]	0.41 [-0.24-0.88]	-0.06 [-0.77-0.41]	2.88	**.028**
Attention, mean (SD)	0.55 [-0.88-1.35]	0.6 [-0.35-1.57]	0.5 [-1.2-1.22]	1.16	1.00
Global Score, mean (SD)	0.84 [-1.68-2.98]	1.8 [0.26-3.96]	-0.41 [-3.44-1.97]	3.10	**.014**

Values represent the median [IQR]. Group-level differences were assessed using the Mann-Whitney U-test. P-values were adjusted for multiple testing using Bonferroni correction, the entire table representing a family of tests. Significant P-values are highlighted in bold.

eTable 24. Comparison of Medication Predictors between LV4 Low- and High-Scorers

	All	Low-Scorer	High-Scorer	*z*	P-value
N	116	61	55		
Diazepam-Eq	0 [0-0]	0 [0-0]	0 [0-77.5]	-3.80	**5.69E-4**
Chlorpromazine-Eq	0 [0-650]	0 [0-0]	0 [0-5123.44]	-3.21	**.005**
Olanzapine-Eq	0 [0-21.7]	0 [0-0]	0 [0-175.63]	-3.21	**.005**
SSRI-Eq	0 [0-88.89]	0 [0-0]	0 [0-350.98]	-2.16	.123

Values represent the median [IQR]. Group-level differences were assessed using the Mann-Whitney U-test. P-values were adjusted for multiple testing using Bonferroni correction, the entire table representing a family of tests. Significant P-values are highlighted in bold. Abbreviations: Eq, Equivalents; SSRI, Selective Serotonin Reuptake Inhibitors.

eFigures

eFigure 1. Observational Study Design of PRONIA

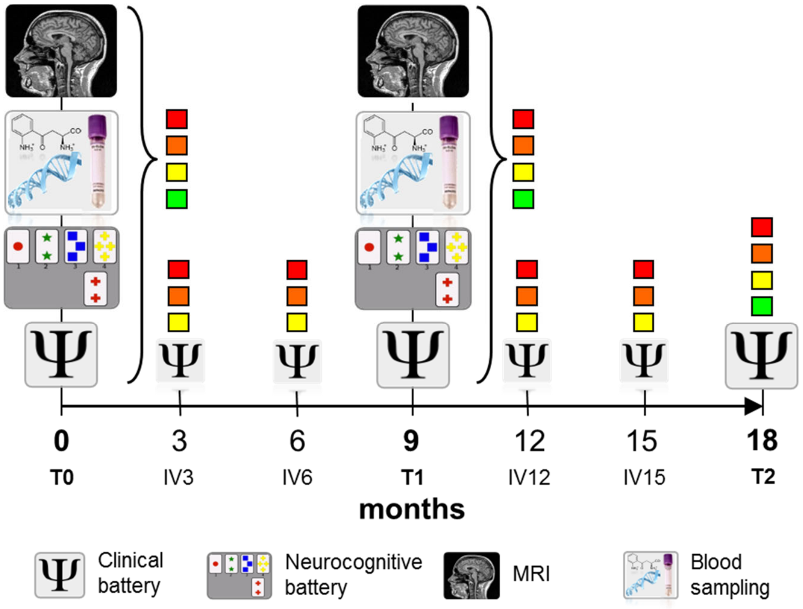

Colored boxes indicate the type of assessment conducted in each of the study groups: Healthy control (HC, green) individuals, patients with recent-onset depression (ROD, yellow), individuals with a clinical high-risk for psychosis (CHR-P, orange), patients with recent-onset psychosis (ROP, red). Individuals recruited at the Munich site were additionally followed up to 36 months, with 9-month intervals after the 18-month follow-up assessment. Previously published in Koutsouleris et al. [2] and reprinted with permission.

eFigure 2. CONSORT Chart and Overview of Analysis Steps

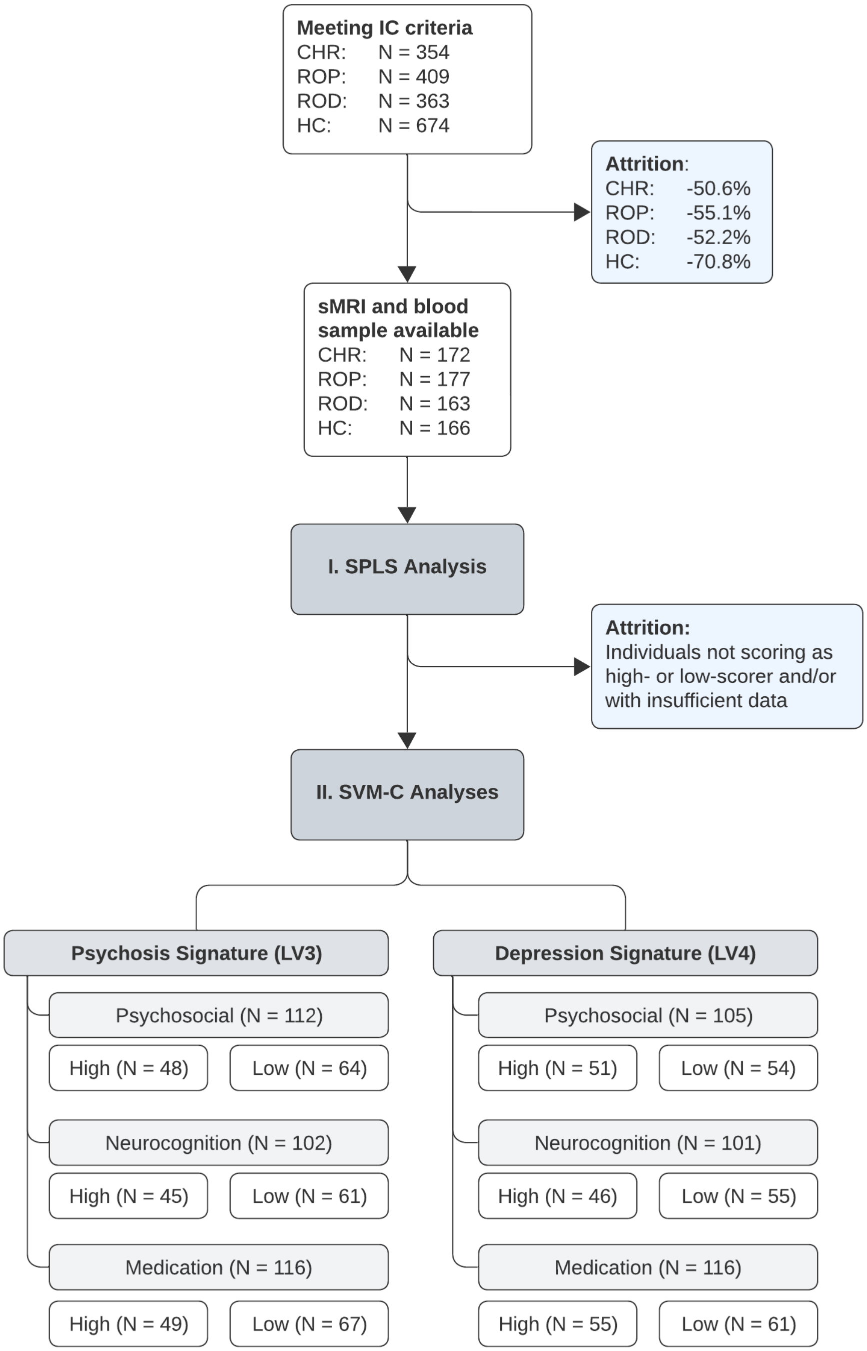

CONSORT chart providing PRONIA study group sample sizes at screening, study inclusion as well as after checking the availability and quality of patients' structural MRI and blood sample data, which entered SPLS analysis and subsequently the SVM-C analyses. Attrition rates per study group after screening and study inclusion due to missing or low-quality data are given in percentages. The latent scores of high- and low-scorers on the psychosis staging (LV3) and the depression (LV4) signature were predicted with psychosocial, neurocognitive and medication data. Sample sizes reduced and varied due to the selection of high- and low-scorers and data availability. *Abbreviations*: CHR-P, Clinical High-Risk for Psychosis; HC, Healthy Control; SPLS; Sparse Partial Least Squares; SVM-C, Support Vector Machine Classification; ROD; Recent-onset Depression; ROP, Recent-onset Psychosis; sMRI, structural Magnetic Resonance Imaging.

eFigure 3. Histogram of CAT12 Image Quality Rating (IQR) of the Study Sample

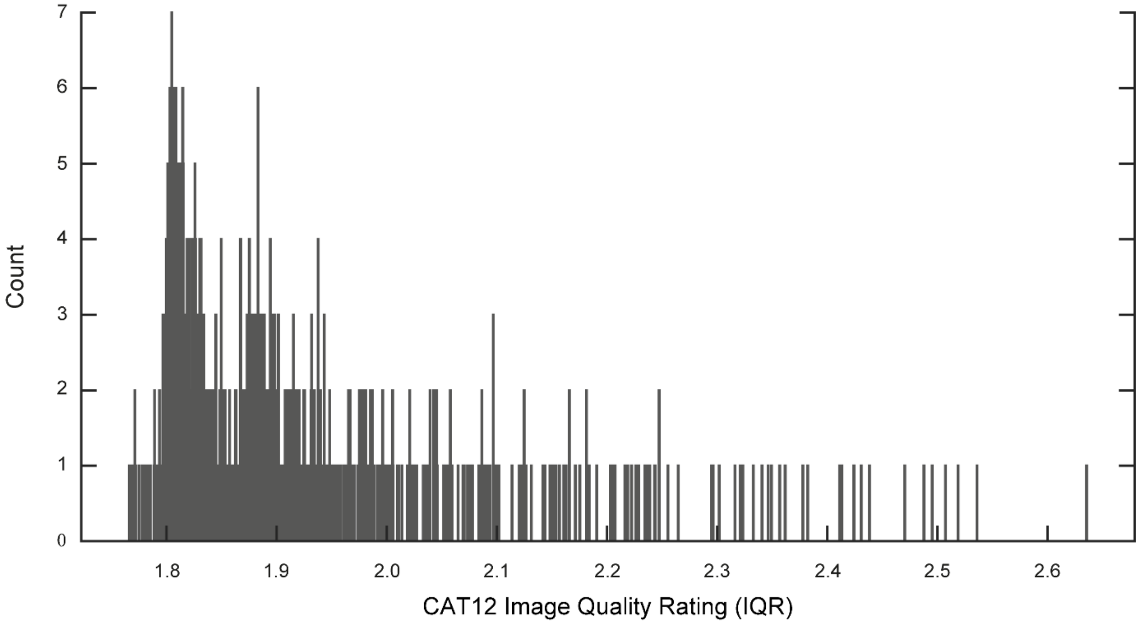

The IQR measure is scaled from 0.5 to 10.5, where 0.5 is a ‘perfect/excellent’ score and 10.5 is deemed ‘unacceptable/failed’. Values around 1 and 2 represent ‘(very) good’ image quality, whereas values of 5 and higher indicate problematic images.[18]

eFigure 4. Nested Cross-Validation Framework

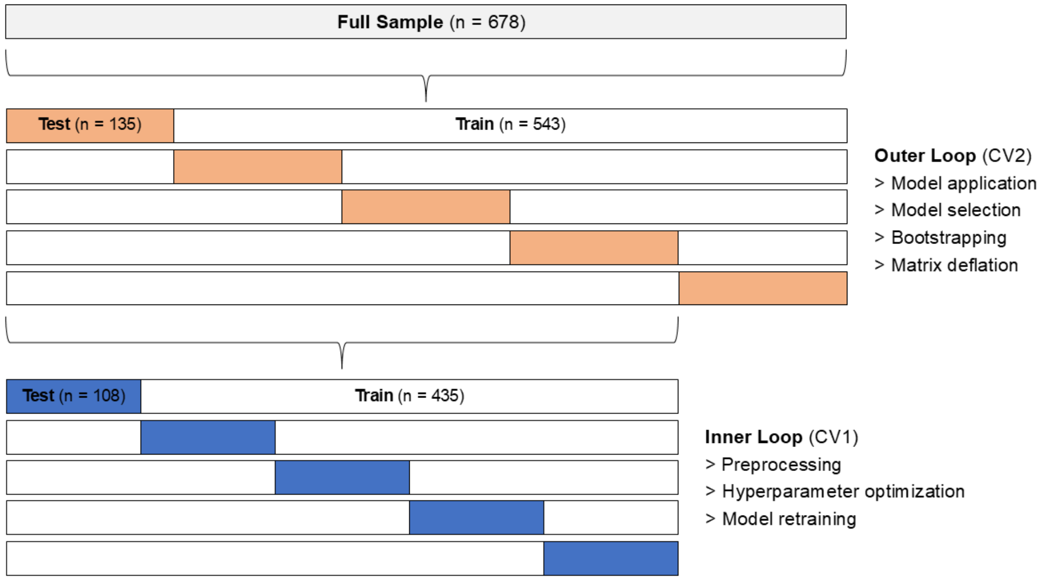

Depicted is the nested cross-validation (NCV) framework with 5x5 folds on the CV2 (Outer loop) and CV1 (Inner loop) level. Preprocessing, hyperparameter optimization of c_u and c_v and model retraining are performed on the CV1 level, whereas testing of the optimized model including model application and selection, bootstrapping as well as matrix deflation are done on the CV2 level. The best model of all 5 CV2 iterations is chosen as the LV model (in accordance with the omnibus hypothesis). Adapted from Popovic et al.[30] and reprinted with permission.

eFigure 5. Age-informed Signature of LV1

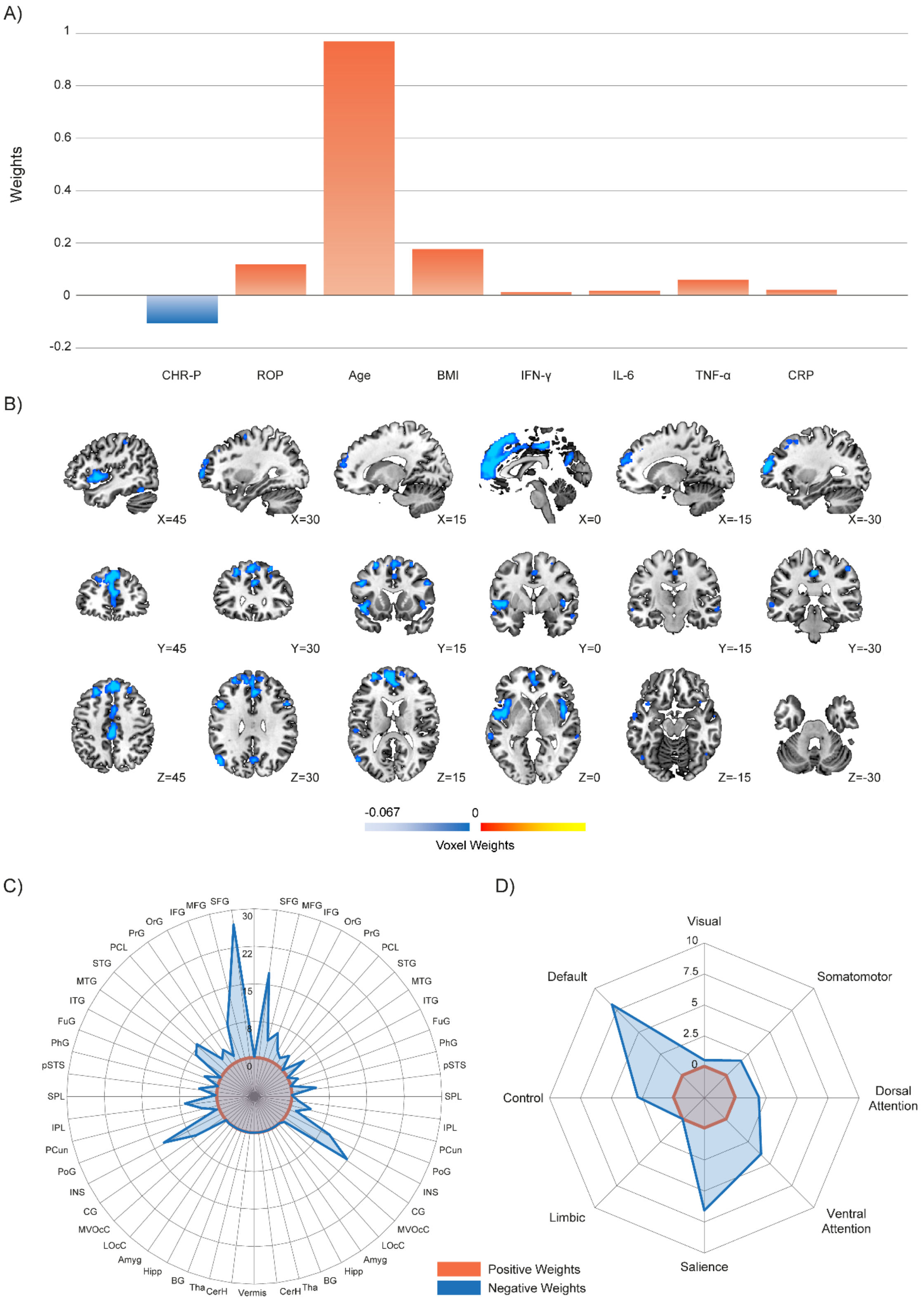

A) The barplot visualizes direction and the values of the weights included in the Blood parameter pattern of LV1. If two feature weights have the same sign (i.e., both positive or both negative), the respective features covary positively with each other, an opposite direction of feature weights represents a negative covariation. Zero weights indicate that there is no significant contribution of the respective features to the covariance signature. Positive weights were assigned to ROP status, age, BMI, IFN-

γ, IL-6, TNF-α and CRP. CHR-P status was negatively weighted. The brain pattern of LV1 was mapped onto the MNI152 standard space via the open-source 3-dimensional rendering software Connectome Workbench v1.4.2 (https://humanconnectome.org/software/connectome-workbench). The spider plots highlight the (C) top neuroanatomic brain regions (derived from the Brainnetome[51] and Diedrichsen[53] atlases) and (D) corresponding functional networks (derived from an adapted, 8-network solution of the Yeo and Buckner atlases) according to the percentage of positively and negatively voxels in these regions. (B-D) Positively weighted voxels are displayed in warm colors, negatively weighted voxels in blue-scale colors. *Abbreviations*: Amyg, Amygdala; BG, Basal Ganglia; BMI, Body Mass Index; CerH, Cerebellum Hemisphere; CG, Cingulate Gyrus; CHR-P, Clinical High-Risk *for Psychosis*; CRP, C-reactive Protein; FuG, Fusiform Gyrus; Hipp, Hippocampus; IFG, Inferior Frontal Gyrus; IFN, Interferon; IL, Interleukin; INS, Insular Gyrus; IPL, Inferior Parietal Lobule; ITG, Inferior Temporal Gyrus; LOcC, Lateral Occipital Cortex; MFG, Middle Frontal Gyrus; MVOcC, MedioVentral Occipital Cortex; MTG, Middle Temporal Gyrus; OrG, Orbital Gyrus; PCL, Paracentral Lobule; PCun, Precuneus; PhG, Parahippocampal Gyrus; PoG, Postcentral Gyrus; PrG, Precentral Gyrus; pSTS, posterior Superior Temporal Sulcus; ROP, Recent-Onset Psychosis; SFG, Superior Frontal Gyrus; SPL, Superior Parietal Lobule; STG, Superior Temporal Gyrus; TFN, Tumor Necrosis Factor; Tha, Thalamus.

eFigure 6. Sex-and IQR-informed Signature of LV2

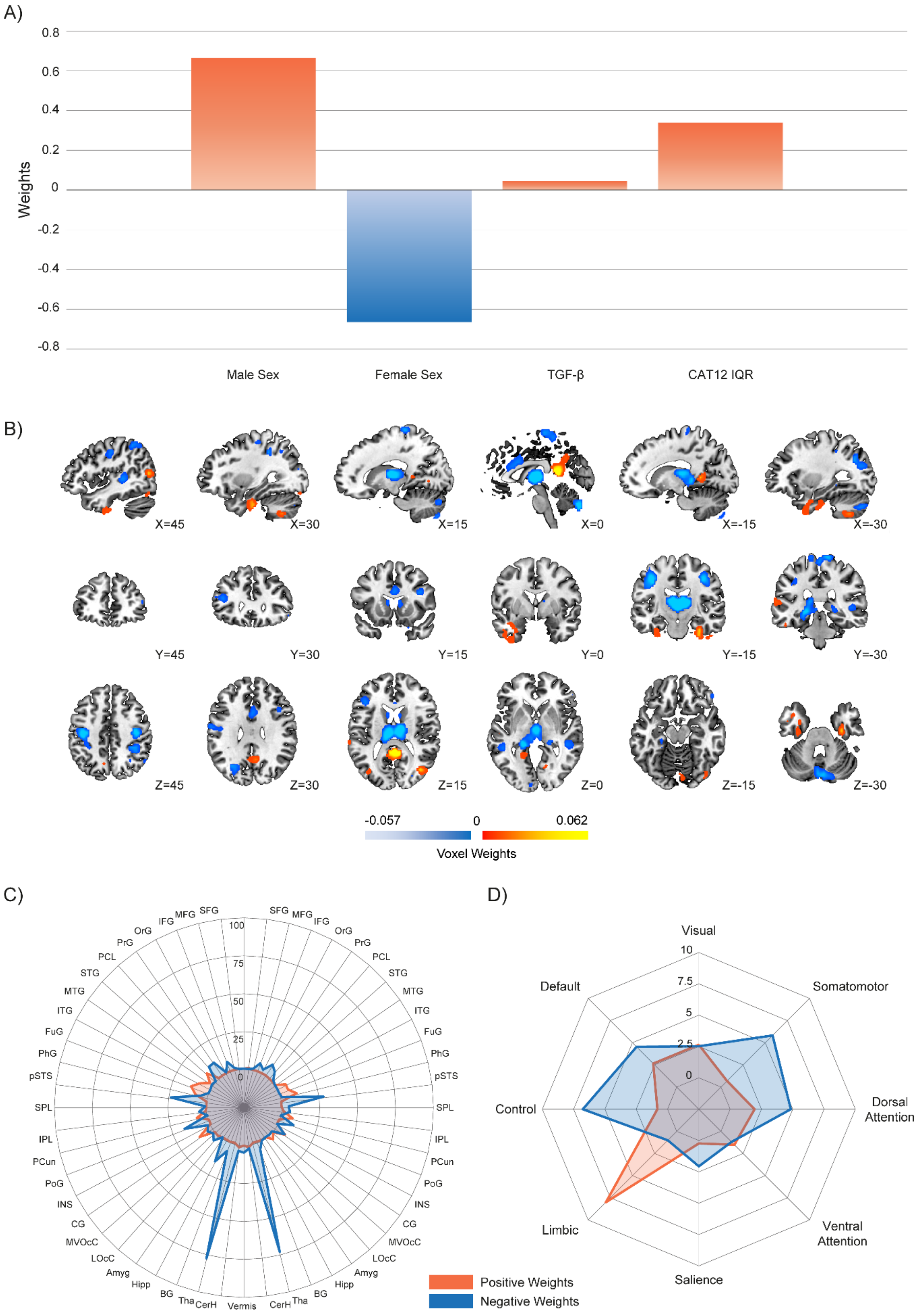

A) The barplot visualizes the direction and the values of the weights included in the Blood parameter pattern of LV2. If two feature weights have the same sign (i.e., both positive or both negative), the respective features covary positively with each other, an opposite direction of feature weights represents a negative covariation. Zero weights indicate that there is no significant

contribution of the respective features to the covariance signature. Male sex, TGF-β and IQR were positively weighted, while female sex received a negative weight. B)-D) see legend eFigure 5. *Abbreviations:* Amyg, Amygdala; BG, Basal Ganglia; CerH, Cerebellum Hemisphere; CG, Cingulate Gyrus; FuG, Fusiform Gyrus; Hipp, Hippocampus; IFG, Inferior Frontal Gyrus; INS, Insular Gyrus; IPL, Inferior Parietal Lobule; ITG, Inferior Temporal Gyrus; IQR, Image Quality Rating; LOcC, Lateral Occipital Cortex; MFG, Middle Frontal Gyrus; MVOcC, MedioVentral Occipital Cortex; MTG, Middle Temporal Gyrus; OrG, Orbital Gyrus; PCL, Paracentral Lobule; PCun, Precuneus; PhG, Parahippocampal Gyrus; PoG, Postcentral Gyrus; PrG, Precentral Gyrus; pSTS, posterior Superior Temporal Sulcus; SFG, Superior Frontal Gyrus; SPL, Superior Parietal Lobule; STG, Superior Temporal Gyrus; TGF, Transforming Growth Factor; Tha, Thalamus.

eFigure 7. Group-level Analysis of the Most Salient Brain and Blood Parameter Features Across All Four LVs

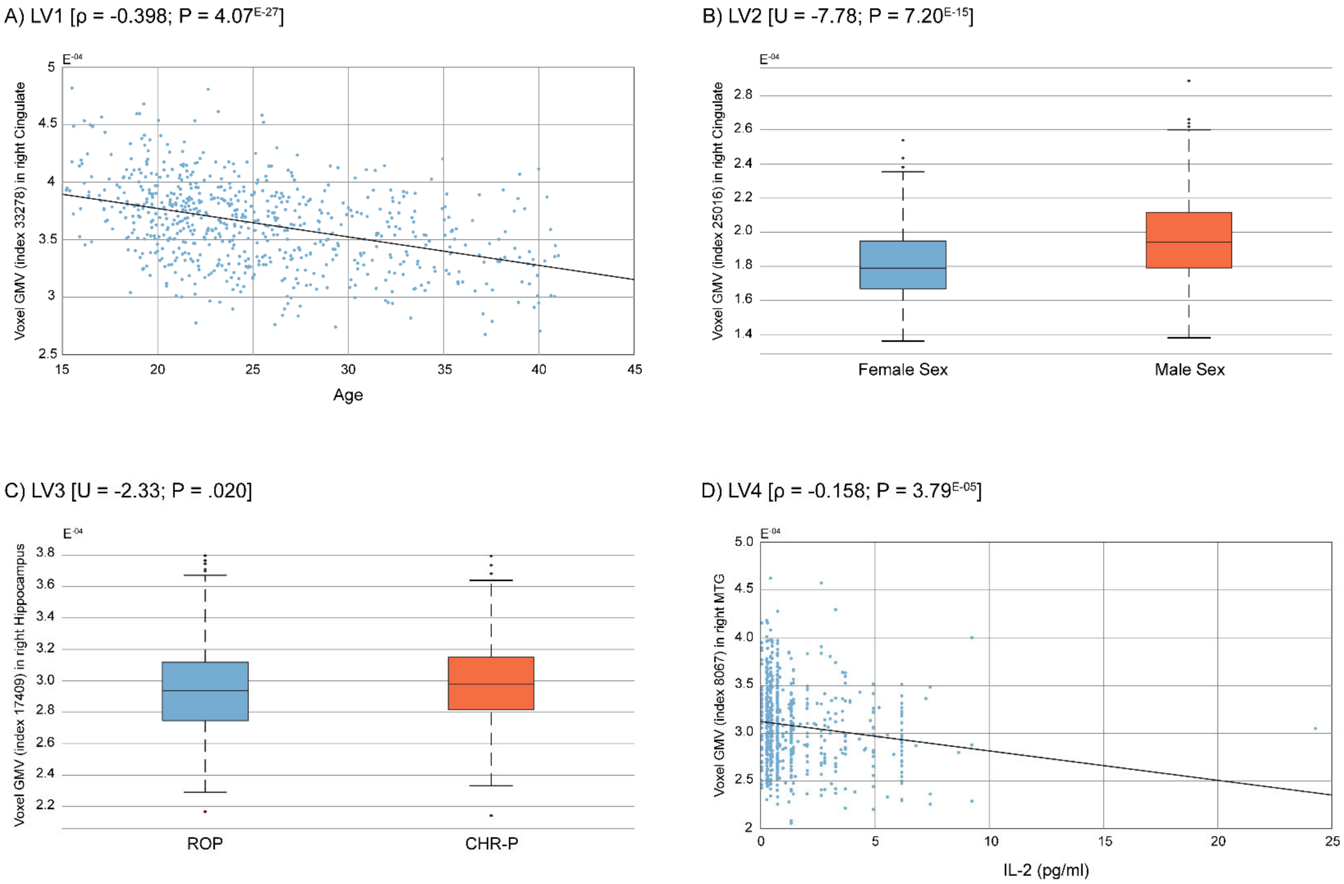

A) Spearman correlation between GMV of voxel 33278 (right Cingulate Gyrus) and age. B) Mann-Whitney-U-Test of GMV of voxel 25016 (right Cingulate Gyrus) between female and male participants. B) Mann-Whitney-U-Test of GMV of voxel 17409 (right Hippocampus) between ROP and CHR-P individuals. D) Spearman correlation between GMV of voxel 8067 (right MTG, Middle Temporal Gyrus) and IL-2 serum levels. The voxel indices were computed from vectorized 3D GMV MR images using the SPM12 toolbox. *Abbreviations:* CHR-P, Clinical High-Risk for Psychosis; IL, Interleukin; MTG, Middle Temporal Gyrus ROP, Recent-Onset Psychosis.

eFigure 8. Medication-based Prediction of LV3 and LV4 High- and Low-Scorers

A) Psychosis Signature

B) Depression Signature

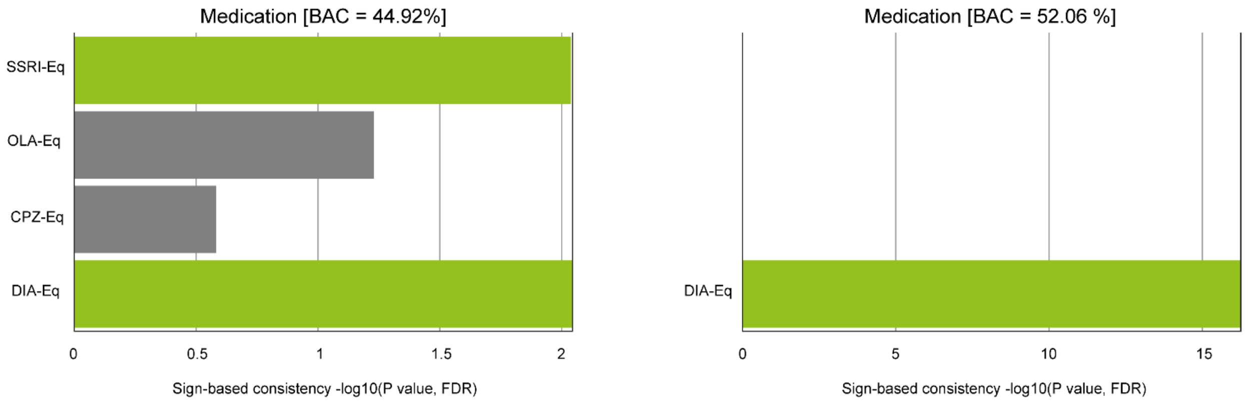

(A-B) Prediction of LV3 (A; BAC = 44.92%) and LV4 (B; BAC = 52.06%) high- and low-scorers using medication data. Abbreviations: BAC, Balanced Accuracy; CPZ, Chlorpromazine; DIA, Diazepam; Eq, Equivalents; OLA, Olanzapine equivalents; SSRI, Selective Serotonin Reuptake Inhibitors.

eFigure 9. Receiver Operating Characteristic (ROC) Curves for SVM Predictive Models of High- and Low-Scorers on LV3 and LV4

A) Psychosis Signature

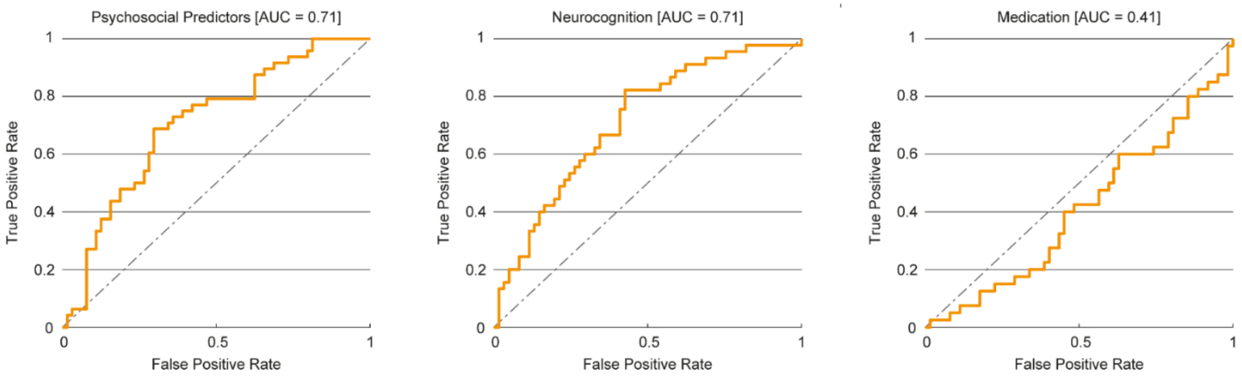

B) Depression Signature

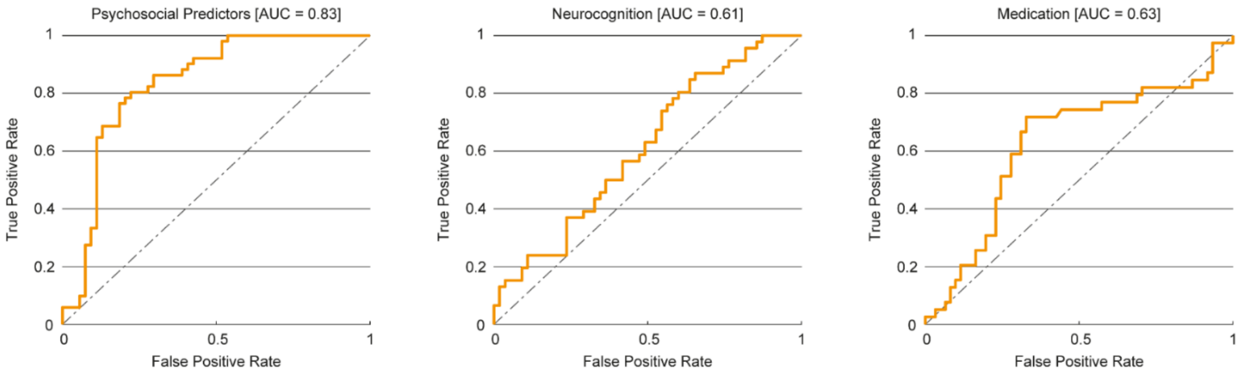

The ROC curves illustrate the diagnostic performance of the SVM models in distinguishing between high- and low scorers on LV3 (A) and LV4 (B) using psychosocial, neurocognitive and medication data. The x-axis represents the false positive rate (1 - specificity), and the y-axis represents the true positive rate (sensitivity). The area under the curve (AUC) values for each model are displayed, indicating the overall discriminative ability, with an AUC of 1.0 representing perfect discrimination and an AUC of 0.5 indicating no discriminative power.

eReferences

1. Bernstein DP, Fink L, Handelsman L, et al. Initial reliability and validity of a new retrospective measure of child abuse and neglect. *Am J Psychiatry*. 1994;151(8):1132-1136. doi:10.1176/ajp.151.8.1132

2. Koutsouleris N, Kambeitz-Ilankovic L, Ruhrmann S, et al. Prediction Models of Functional Outcomes for Individuals in the Clinical High-Risk State for Psychosis or With Recent-Onset Depression: A Multimodal, Multisite Machine Learning Analysis. *JAMA Psychiatry*. 2018;75(11):1156-1172. doi:10.1001/jamapsychiatry.2018.2165

3. Schultze-Lutter F, Addington J, Ruhrmann S, Klosterkötter J. Schizophrenia Proneness Instrument – Adult Version. Published online 2007.

4. Fux L, Walger P, Schimmelmann B, Schultze-Lutter F. The Schizophrenia Proneness Instrument, Child and Youth version (SPI-CY): Practicability and discriminative validity. *Schizophr Res*. 2013;146:69-78. doi:10.1016/j.schres.2013.02.014

5. Miller TJ, McGlashan TH, Rosen JL, et al. Prodromal assessment with the structured interview for prodromal syndromes and the scale of prodromal symptoms: predictive validity, interrater reliability, and training to reliability. *Schizophr Bull*. 2003;29(4):703-715. https://www.ncbi.nlm.nih.gov/pubmed/14989408

6. Gaebel W, Hasan A, Falkai P. *S3-Leitlinie Schizophrenie*. Vol 1. Deutsche Gesellschaft für Psychiatrie und Psychotherapie, Psychosomatik und Nervenheilkunde e. V. (DGPPN); 2019. https://www.awmf.org/leitlinien/detail/ll/038-009.html

7. Kay SR, Fiszbein A, Opler LA. The positive and negative syndrome scale (PANSS) for schizophrenia. *Schizophr Bull*. 1987;13(2):261-276. doi:10.1093/schbul/13.2.261

8. Beck AT, Steer RA. Internal consistencies of the original and revised Beck Depression Inventory. *J Clin Psychol*. 1984;40(6):1365-1367. doi:10.1002/1097-4679(198411)40:6<1365::aid-jclp2270400615>3.0.co;2-d

9. Pedersen G, Hagtvet KA, Karterud S. Generalizability studies of the Global Assessment of Functioning-Split version. *Compr Psychiatry*. 2007;48(1):88-94. doi:10.1016/j.comppsych.2006.03.008

10. Cornblatt BA, Auther AM, Niendam T, et al. Preliminary findings for two new measures of social and role functioning in the prodromal phase of schizophrenia. *Schizophr Bull*. 2007;33(3):688-702. doi:10.1093/schbul/sbm029

11. Cannon-Spoor HE, Potkin SG, Wyatt RJ. Measurement of premorbid adjustment in chronic schizophrenia. *Schizophr Bull*. 1982;8(3):470-484. doi:10.1093/schbul/8.3.470

12. Costa PT, McCrae RR. *Revised NEO Personality Inventory (NEO PI-R) and NEO Five-Factor Inventory (NEO-FFI)*. Psychological Assessment Resources; 1992.

13. Skevington SM, Lotfy M, O'Connell KA, WHOQOL Group. The World Health Organization's WHOQOL-BREF quality of life assessment: psychometric properties and results of the international field trial. A report from the WHOQOL group. *Qual Life Res*. 2004;13(2):299-310. doi:10.1023/B:QURE.0000018486.91360.00

14. Nuechterlein KH, Green MF, Kern RS, et al. The MATRICS Consensus Cognitive Battery, part 1: test selection, reliability, and validity. *Am J Psychiatry*. 2008;165(2):203-213. doi:10.1176/appi.ajp.2007.07010042

15. Haas SS, Antonucci LA, Wenzel J, et al. A multivariate neuromonitoring approach to neuroplasticity-based computerized cognitive training in recent onset psychosis. *Neuropsychopharmacology*. Published online October 7, 2020. doi:10.1038/s41386-020-00877-4

16. Manjon JV, Tohka J, Garcia-Marti G, et al. Robust MRI brain tissue parameter estimation by multistage outlier rejection. *Magn Reson Med*. 2008;59(4):866-873. doi:10.1002/mrm.21521

17. Rajapakse JC, Giedd JN, Rapoport JL. Statistical approach to segmentation of single-channel cerebral MR images. *IEEE Trans Med Imaging*. 1997;16(2):176-186. doi:10.1109/42.563663

18. Collins DL, Zijdenbos AP, Kollokian V, et al. Design and construction of a realistic digital brain phantom. *IEEE Transactions on Medical Imaging*. 1998;17(3):463-468. doi:10.1109/42.712135

19. Pardoe HR, Kucharsky Hiess R, Kuzniecky R. Motion and morphometry in clinical and nonclinical populations. *Neuroimage*. 2016;135:177-185. doi:10.1016/j.neuroimage.2016.05.005

20. Savalia NK, Agres PF, Chan MY, Feczko EJ, Kennedy KM, Wig GS. Motion-related artifacts in structural brain images revealed with independent estimates of in-scanner head motion. *Hum Brain Mapp*. 2017;38(1):472-492. doi:10.1002/hbm.23397

21. McMinn MR, Wiens AN, Crossen JR. Rey auditory-verbal learning test: Development of norms for healthy young adults. *Clin neuropsychol*. 1988;2(1):67-87. doi:10.1080/13854048808520087

22. Benedict RHB, Schretlen D, Groninger L, Brandt J. Hopkins verbal learning test – revised: Normative data and analysis of inter-form and test-retest reliability. *Clin Neuropsychol*. 1998;12(1):43-55. doi:10.1076/clin.12.1.43.1726

23. Penzel N, Antonucci AA, L, Betz. Association between Age of Cannabis Initiation and Gray Matter Covariance Networks in Recent Onset Psychosis. *Neuropsychopharmachology*.

24. Nowicki S Jr, Duke MP. Individual differences in the nonverbal communication of affect: The diagnostic analysis of nonverbal accuracy scale. *J Nonverbal Behav*. 1994;18(1):9-35. doi:10.1007/bf02169077

25. Wechsler D, Coalson DL, Raiford SE. *Wechsler Adult Intelligence Scale-Fourth Edition*. Pearson; 2008.

26. Sánchez-Cubillo I, Periáñez JA, Adrover-Roig D, et al. Construct validity of the Trail Making Test: role of task-switching, working memory, inhibition/interference control, and visuomotor abilities. *J Int Neuropsychol Soc*. 2009;15(3):438-450. doi:10.1017/S1355617709090626

27. Harrison JE, Buxton P, Husain M, Wise R. Short test of semantic and phonological fluency: Normal performance, validity and test-retest reliability. *Br J Clin Psychol*. 2000;39(2):181-191. doi:10.1348/014466500163202

28. Cornblatt BA, Risch NJ, Faris G, Friedman D, Erlenmeyer-Kimling L. The Continuous Performance Test, identical pairs version (CPT-IP): I. New findings about sustained attention in normal families. *Psychiatry Res*. 1988;26(2):223-238. doi:10.1016/0165-1781(88)90076-5

29. Monteiro JM, Rao A, Shawe-Taylor J, Mourão-Miranda J. A multiple hold-out framework for Sparse Partial Least Squares. *J Neurosci Methods*. 2016;271:182-194. doi:10.1016/j.jneumeth.2016.06.011

30. Popovic D, Ruef A, Dwyer DB, et al. Traces of trauma: A multivariate pattern analysis of childhood trauma, brain structure, and clinical phenotypes. *Biol Psychiatry*. 2020;88(11):829-842. doi:10.1016/j.biopsych.2020.05.020

31. SPLS Toolbox (2022). GitHub. Accessed November 1, 2024. https://github.molgen.mpg.de/DavidPopovic/SPLS_Toolbox_2022

32. Wegelin, Jacob A %J University of Washington, Rep T. A survey of Partial Least Squares (PLS) methods, with emphasis on the two-block case. Published online 2000.

33. Witten DM, Tibshirani R, Hastie T %J B. A penalized matrix decomposition, with applications to sparse principal components and canonical correlation analysis. 2009;10(3):515-534.

34. Zou H, Hastie T. Regularization and variable selection via the elastic net. 2005;67(2):301-320. doi:10.1111/j.1467-9868.2005.00503.x

35. Witten DM, Tibshirani RJ. Extensions of sparse canonical correlation analysis with applications to genomic data. *Stat Appl Genet Mol Biol*. 2009;8:Article28. doi:10.2202/1544-6115.1470

36. Mackey L. Deflation Methods for Sparse PCA. *Advances in Neural Information Processing Systems 21 - Proceedings of the 2008 Conference*. 2008;21:1017-1024.

37. Monteiro JM, Rao A, Ashburner J, Shawe-Taylor J, Mourão-Miranda J. *Leveraging Clinical Data to Enhance Localization of Brain Atrophy*. (Langs G, Wehbe L, Cecchi G, Chang KMK, Murphy B, eds.). Springer International Publishing; 2016.

38. Nichols TE, Holmes AP. Nonparametric permutation tests for functional neuroimaging: a primer with examples. *Hum Brain Mapp*. 2002;15(1):1-25. doi:10.1002/hbm.1058

39. Dwyer DB, Falkai P, Koutsouleris N. Machine Learning Approaches for Clinical Psychology and Psychiatry. *Annu Rev Clin Psychol*. 2018;14:91-118. doi:10.1146/annurev-clinpsy-032816-045037

40. Dukart J, Schroeter ML, Mueller K, Alzheimer's Disease Neuroimaging Initiative. Age correction in dementia--matching to a healthy brain. *PLoS One*. 2011;6(7):e22193. doi:10.1371/journal.pone.0022193

41. Krishnan A, Williams LJ, McIntosh AR, Abdi H. Partial Least Squares (PLS) methods for neuroimaging: a tutorial and review. *Neuroimage*. 2011;56(2):455-475. doi:10.1016/j.neuroimage.2010.07.034

42. Koutsouleris N, Kahn RS, Chekroud AM, et al. Multisite prediction of 4-week and 52-week treatment outcomes in patients with first-episode psychosis: a machine learning approach. *Lancet Psychiatry*. 2016;3(10):935-946. doi:10.1016/S2215-0366(16)30171-7

43. Ruschhaupt M, Huber W, Poustka A, Mansmann U. A compendium to ensure computational reproducibility in high-dimensional classification tasks. *Stat Appl Genet Mol Biol*. 2004;3:Article37. doi:10.2202/1544-6115.1078

44. Varma S, Simon R. Bias in error estimation when using cross-validation for model selection. *BMC Bioinformatics*. 2006;7:91. doi:10.1186/1471-2105-7-91

45. Varmuza K, Filzmoser P, Hilchenbach M, Krüger H, Silén J. KNN classification — evaluated by repeated double cross validation: Recognition of minerals relevant for comet dust. *Chemometrics and Intelligent Laboratory Systems*. 2014;138:64-71. doi:10.1016/j.chemolab.2014.07.011

46. Saeys Y, Inza I, Larrañaga P. A review of feature selection techniques in bioinformatics. *Bioinformatics*. 2007;23(19):2507-2517. doi:10.1093/bioinformatics/btm344

47. Noble WS. What is a support vector machine? *Nat Biotechnol*. 2006;24(12):1565-1567. https://www.nature.com/articles/nbt1206-1565

48. Koutsouleris N, Riecher-Rössler A, Meisenzahl EM, et al. Detecting the psychosis prodrome across high-risk populations using neuroanatomical biomarkers. *Schizophr Bull*. 2015;41(2):471-482. doi:10.1093/schbul/sbu078

49. Lalousis PA, Malaviya A, Khatibi A, et al. Anhedonia as a potential transdiagnostic phenotype with immune-related changes in recent-onset mental health disorders. *Biol Psychiatry*. 2024;96(7):615-622. doi:10.1016/j.biopsych.2024.05.019

50. SPM12. Accessed November 1, 2024. https://www.fil.ion.ucl.ac.uk/spm/software/spm12/

51. Fan L, Li H, Zhuo J, et al. The Human Brainnetome Atlas: A New Brain Atlas Based on Connectional Architecture. *Cereb Cortex*. 2016;26(8):3508-3526. doi:10.1093/cercor/bhw157

52. Brainnetome Atlas. Accessed November 1, 2024. http://atlas.brainnetome.org/index.html

53. Diedrichsen J, Balsters JH, Flavell J, Cussans E, Ramnani N. A probabilistic MR atlas of the human cerebellum. *Neuroimage*. 2009;46(1):39-46. doi:10.1016/j.neuroimage.2009.01.045

54. SUIT - Probabilistic atlas of the human cerebellum. Accessed November 1, 2024. https://diedrichsenlab.org/imaging/propatlas.htm

55. Fjell AM, Walhovd KB. Structural brain changes in aging: courses, causes and cognitive consequences. *Rev Neurosci*. 2010;21(3):187-221. doi:10.1515/revneuro.2010.21.3.187

56. Jernigan TL, Archibald SL, Fennema-Notestine C, et al. Effects of age on tissues and regions of the cerebrum and cerebellum. *Neurobiol Aging*. 2001;22(4):581-594. doi:10.1016/s0197-4580(01)00217-2

57. Blinkouskaya Y, Caçoilo A, Gollamudi T, Jalalian S, Weickenmeier J. Brain aging mechanisms with mechanical manifestations. *Mech Ageing Dev*. 2021;200:111575. doi:10.1016/j.mad.2021.111575

58. Ferreira LK, Busatto GF. Resting-state functional connectivity in normal brain aging. *Neuroscience & Biobehavioral Reviews*. 2013;37(3):384-400. doi:10.1016/j.neubiorev.2013.01.017

59. Vidal-Piñeiro D, Valls-Pedret C, Fernández-Cabello S, et al. Decreased Default Mode Network connectivity correlates with age-associated structural and cognitive changes. *Front Aging Neurosci*. 2014;6:256. doi:10.3389/fnagi.2014.00256

60. Reas DL, Nygård JF, Svensson E, Sørensen T, Sandanger I. Changes in body mass index by age, gender, and socio-economic status among a cohort of Norwegian men and women (1990–2001). *BMC Public Health*. 2007;7(1):269. doi:10.1186/1471-2458-7-269

61. Ruigrok ANV, Salimi-Khorshidi G, Lai MC, et al. A meta-analysis of sex differences in human brain structure. *Neurosci Biobehav Rev*. 2014;39:34-50. doi:10.1016/j.neubiorev.2013.12.004

62. Lotze M, Domin M, Gerlach FH, et al. Novel findings from 2,838 Adult Brains on Sex Differences in Gray Matter Brain Volume. *Sci Rep*. 2019;9(1):1671. doi:10.1038/s41598-018-38239-2

63. Ritchie SJ, Cox SR, Shen X, et al. Sex Differences in the Adult Human Brain: Evidence from 5216 UK Biobank Participants. *Cerebral Cortex*. 2018;28(8):2959-2975. doi:10.1093/cercor/bhy109

64. DeCasien AR, Guma E, Liu S, Raznahan A. Sex differences in the human brain: a roadmap for more careful analysis and interpretation of a biological reality. *Biology of Sex Differences*. 2022;13(1):43. doi:10.1186/s13293-022-00448-w

65. Williams CM, Peyre H, Toro R, Ramus F. Sex differences in the brain are not reduced to differences in body size. *Neuroscience & Biobehavioral Reviews*. 2021;130:509-511. doi:10.1016/j.neubiorev.2021.09.015

66. Lentini E, Kasahara M, Arver S, Savic I. Sex differences in the human brain and the impact of sex chromosomes and sex hormones. *Cereb Cortex*. 2013;23(10):2322-2336. doi:10.1093/cercor/bhs222

67. Gur RC, Gur RE. Complementarity of sex differences in brain and behavior: From laterality to multimodal neuroimaging. *J Neurosci Res*. 2017;95(1-2):189-199. doi:10.1002/jnr.23830

68. Gur RE, Maany V, Mozley PD, Swanson C, Bilker W, Gur RC. Subcortical MRI volumes in neuroleptic-naive and treated patients with schizophrenia. *Am J Psychiatry*. 1998;155(12):1711-1717. doi:10.1176/ajp.155.12.1711

69. Reuter M, Tisdall MD, Qureshi A, Buckner RL, van der Kouwe AJW, Fischl B. Head motion during MRI acquisition reduces gray matter volume and thickness estimates. *Neuroimage*. 2015;107:107-115. doi:10.1016/j.neuroimage.2014.12.006

70. Rosen AFG, Roalf DR, Ruparel K, et al. Quantitative assessment of structural image quality. *Neuroimage*. 2018;169:407-418. doi:10.1016/j.neuroimage.2017.12.059

71. Alexander-Bloch A, Clasen L, Stockman M, et al. Subtle in-scanner motion biases automated measurement of brain anatomy from in vivo MRI. *Hum Brain Mapp*. 2016;37(7):2385-2397. doi:10.1002/hbm.23180

72. Siegel JS, Mitra A, Laumann TO, et al. Data quality influences observed links between functional connectivity and behavior. *Cereb Cortex*. 2017;27(9):4492-4502. doi:10.1093/cercor/bhw253
