# Supplementary material for: Multivariate Brain-Blood Signatures in Early-Stage Depression and Psychosis
Source: JAMA Psychiatry. 2025 Dec 17;83(2):172–84. doi: 10.1001/jamapsychiatry.2025.3803 (PMC12712837; doi:10.1001/jamapsychiatry.2025.3803)
Supplement: Supplement 3. — PRONIA Consortium members [file jamapsychiatry-e253803-s003.pdf]

Supplemental Online Content: Nonauthor Collaborators
*First name, last name, and suffix (if applicable) are required and will appear in PubMed.

***Group Name(s): PRONIA Consortium**							
***First Name and Middle Initial(s)**	***Last Name**	***Suffix (eg, Jr, III)**	Academic Degrees	Institution	Location (city, state/province, country)	Role or Contribution, eg, chair, principal investigator	Group (if more than 1 Group listed in the byline) and/or Subgroup (eg, Steering Committee)
Shalaila	Haas			Department of Psychiatry and Psychotherapy, Ludwig-Maximilian-University, Munich, Germany	Munich, Germany	Researcher	
Alkomiet	Hasan			Department of Psychiatry and Psychotherapy, Ludwig-Maximilian-University, Munich, Germany	Munich, Germany	Researcher	
Claudius	Hoff			Department of Psychiatry and Psychotherapy, Ludwig-Maximilian-University, Munich, Germany	Munich, Germany	Researcher	
Ifrah	Khanyaree			Department of Psychiatry and Psychotherapy, Ludwig-Maximilian-University, Munich, Germany	Munich, Germany	Researcher	
Camilla	Krämer			Department of Psychiatry and Psychotherapy, Ludwig-Maximilian-University, Munich, Germany	Munich, Germany	Researcher	
Aylin	Melo			Department of Psychiatry and Psychotherapy, Ludwig-Maximilian-University, Munich, Germany	Munich, Germany	Researcher	
Susanna	Muckenhuber-Sternbauer			Department of Psychiatry and Psychotherapy, Ludwig-Maximilian-University, Munich, Germany	Munich, Germany	Researcher	

Supplemental Online Content: Nonauthor Collaborators
*First name, last name, and suffix (if applicable) are required and will appear in PubMed.

***First Name and Middle Initial(s)**	***Last Name**	***Suffix (eg, Jr, III)**	Academic Degrees	Institution	Location (city, state/province, country)	Role or Contribution, eg, chair, principal investigator	Group (if more than 1 Group listed in the byline) and/or Subgroup (eg, Steering Committee)
Yanis	Köhler			Department of Psychiatry and Psychotherapy, Ludwig-Maximilian-University, Munich, Germany	Munich, Germany	Researcher	
Ömer	Öztürk			Department of Psychiatry and Psychotherapy, Ludwig-Maximilian-University, Munich, Germany	Munich, Germany	Researcher	
Nora	Penzel			Department of Psychiatry and Psychotherapy, Ludwig-Maximilian-University, Munich, Germany	Munich, Germany	Researcher	
Adrian	Rangnick			Department of Psychiatry and Psychotherapy, Ludwig-Maximilian-University, Munich, Germany	Munich, Germany	Researcher	
Sebastian	von Saldern			Department of Psychiatry and Psychotherapy, Ludwig-Maximilian-University, Munich, Germany	Munich, Germany	Researcher	
Rachele	Sanfelici			Department of Psychiatry and Psychotherapy, Ludwig-Maximilian-University, Munich, Germany	Munich, Germany	Researcher	
Moritz	Spangemacher			Department of Psychiatry and Psychotherapy, Ludwig-Maximilian-University, Munich, Germany	Munich, Germany	Researcher	

*First name, last name, and suffix (if applicable) are required and will appear in PubMed.

***First Name and Middle Initial(s)**	***Last Name**	***Suffix (eg, Jr, III)**	Academic Degrees	Institution	Location (city, state/province, country)	Role or Contribution, eg, chair, principal investigator	Group (if more than 1 Group listed in the byline) and/or Subgroup (eg, Steering Committee)
Ana	Tupac			Department of Psychiatry and Psychotherapy, Ludwig-Maximilian-University, Munich, Germany	Munich, Germany	Researcher	
Maria Fernanda	Urquijo			Department of Psychiatry and Psychotherapy, Ludwig-Maximilian-University, Munich, Germany	Munich, Germany	Researcher	
Johanna	Weiske			Department of Psychiatry and Psychotherapy, Ludwig-Maximilian-University, Munich, Germany	Munich, Germany	Researcher	
Antonia	Wosgien			Department of Psychiatry and Psychotherapy, Ludwig-Maximilian-University, Munich, Germany	Munich, Germany	Researcher	
Karsten	Blume			Department of Psychiatry and Psychotherapy, University of Cologne, Cologne, Germany	Cologne, Germany	Researcher	
Dennis	Hedderich			Department of Psychiatry and Psychotherapy, University of Cologne, Cologne, Germany	Cologne, Germany	Researcher	
Dominika	Julkowski			Department of Psychiatry and Psychotherapy, University of Cologne, Cologne, Germany	Cologne, Germany	Researcher	
Nathalie	Kaiser			Department of Psychiatry and Psychotherapy, University of Cologne, Cologne, Germany	Cologne, Germany	Researcher	
Thorsten	Lichtenstein			Department of Psychiatry and Psychotherapy, University of Cologne, Cologne, Germany	Cologne, Germany	Researcher	

Supplemental Online Content: Nonauthor Collaborators
*First name, last name, and suffix (if applicable) are required and will appear in PubMed.

***First Name and Middle Initial(s)**	***Last Name**	***Suffix (eg, Jr, III)**	Academic Degrees	Institution	Location (city, state/province, country)	Role or Contribution, eg, chair, principal investigator	Group (if more than 1 Group listed in the byline) and/or Subgroup (eg, Steering Committee)
Ruth	Milz			Department of Psychiatry and Psychotherapy, University of Cologne, Cologne, Germany	Cologne, Germany	Researcher	
Alexandra	Nikolaides			Department of Psychiatry and Psychotherapy, University of Cologne, Cologne, Germany	Cologne, Germany	Researcher	
Tanja	Pilgram			Department of Psychiatry and Psychotherapy, University of Cologne, Cologne, Germany	Cologne, Germany	Researcher	
Mauro	Seves			Department of Psychiatry and Psychotherapy, University of Cologne, Cologne, Germany	Cologne, Germany	Researcher	
Martina	Wassen			Department of Psychiatry and Psychotherapy, University of Cologne, Cologne, Germany	Cologne, Germany	Researcher	
Christina	Andreou			Department of Psychiatry (Psychiatric University Hospital, UPK), University of Basel, Switzerland	Basel, Switzerland	Researcher	
Laura	Egloff			Department of Psychiatry (Psychiatric University Hospital, UPK), University of Basel, Switzerland	Basel, Switzerland	Researcher	
Fabienne	Harrisberger			Department of Psychiatry (Psychiatric University Hospital, UPK), University of Basel, Switzerland	Basel, Switzerland	Researcher	
Ulrike	Heitz			Department of Psychiatry (Psychiatric University Hospital, UPK), University of Basel, Switzerland	Basel, Switzerland	Researcher	

*First name, last name, and suffix (if applicable) are required and will appear in PubMed.

***First Name and Middle Initial(s)**	***Last Name**	***Suffix (eg, Jr, III)**	Academic Degrees	Institution	Location (city, state/province, country)	Role or Contribution, eg, chair, principal investigator	Group (if more than 1 Group listed in the byline) and/or Subgroup (eg, Steering Committee)
Claudia	Lenz			Department of Psychiatry (Psychiatric University Hospital, UPK), University of Basel, Switzerland	Basel, Switzerland	Researcher	
Letizia	Leanza			Department of Psychiatry (Psychiatric University Hospital, UPK), University of Basel, Switzerland	Basel, Switzerland	Researcher	
Amatya	Mackintosh			Department of Psychiatry (Psychiatric University Hospital, UPK), University of Basel, Switzerland	Basel, Switzerland	Researcher	
Renata	Smieskova			Department of Psychiatry (Psychiatric University Hospital, UPK), University of Basel, Switzerland	Basel, Switzerland	Researcher	
Erich	Studerus			Department of Psychiatry (Psychiatric University Hospital, UPK), University of Basel, Switzerland	Basel, Switzerland	Researcher	
Anna	Walter			Department of Psychiatry (Psychiatric University Hospital, UPK), University of Basel, Switzerland	Basel, Switzerland	Researcher	
Sonja	Widmayer			Department of Psychiatry (Psychiatric University Hospital, UPK), University of Basel, Switzerland	Basel, Switzerland	Researcher	
Chris	Day			Institute of Mental Health & School of Psychology, University of Birmingham, United Kingdom	Birmingham, United Kingdom	Researcher	

Supplemental Online Content: Nonauthor Collaborators
*First name, last name, and suffix (if applicable) are required and will appear in PubMed.

***First Name and Middle Initial(s)**	***Last Name**	***Suffix (eg, Jr, III)**	Academic Degrees	Institution	Location (city, state/province, country)	Role or Contribution, eg, chair, principal investigator	Group (if more than 1 Group listed in the byline) and/or Subgroup (eg, Steering Committee)
Mariam	Iqbal			Institute of Mental Health & School of Psychology, University of Birmingham, United Kingdom	Birmingham, United Kingdom	Researcher	
Mirabel	Pelton			Institute of Mental Health & School of Psychology, University of Birmingham, United Kingdom	Birmingham, United Kingdom	Researcher	
Pavan	Mallikarjun			Institute of Mental Health & School of Psychology, University of Birmingham, United Kingdom	Birmingham, United Kingdom	Researcher	
Alexandra	Stainton			Institute of Mental Health & School of Psychology, University of Birmingham, United Kingdom	Birmingham, United Kingdom	Researcher	
Ashleigh	Lin			Institute of Mental Health & School of Psychology, University of Birmingham, United Kingdom	Birmingham, United Kingdom	Researcher	
Alexander	Denissoff			Department of Psychiatry, University of Turku, Finland	Turku, Finland	Researcher	
Anu	Ellilä			Department of Psychiatry, University of Turku, Finland	Turku, Finland	Researcher	
Tiina	From			Department of Psychiatry, University of Turku, Finland	Turku, Finland	Researcher	
Markus	Heinimaa			Department of Psychiatry, University of Turku, Finland	Turku, Finland	Researcher	
Tuula	Ilonen			Department of Psychiatry, University of Turku, Finland	Turku, Finland	Researcher	
Päivi	Jalo			Department of Psychiatry, University of Turku, Finland	Turku, Finland	Researcher	
Heikki	Laurikainen			Department of Psychiatry, University of Turku, Finland	Turku, Finland	Researcher	
Antti	Luutonen			Department of Psychiatry, University of Turku, Finland	Turku, Finland	Researcher	

*First name, last name, and suffix (if applicable) are required and will appear in PubMed.

***First Name and Middle Initial(s)**	***Last Name**	***Suffix (eg, Jr, III)**	Academic Degrees	Institution	Location (city, state/province, country)	Role or Contribution, eg, chair, principal investigator	Group (if more than 1 Group listed in the byline) and/or Subgroup (eg, Steering Committee)
Akseli	Mäkela			Department of Psychiatry, University of Turku, Finland	Turku, Finland	Researcher	
Janina	Paju			Department of Psychiatry, University of Turku, Finland	Turku, Finland	Researcher	
Henri	Pesonen			Department of Psychiatry, University of Turku, Finland	Turku, Finland	Researcher	
Reetta-Liina	Säilä			Department of Psychiatry, University of Turku, Finland	Turku, Finland	Researcher	
Anna	Toivonen			Department of Psychiatry, University of Turku, Finland	Turku, Finland	Researcher	
Otto	Turtonen			Department of Psychiatry, University of Turku, Finland	Turku, Finland	Researcher	
Sonja	Botterweck			Department of Psychiatry (Psychiatric University Hospital LVR/HHU Düsseldorf), University of Düsseldorf, Germany	Düsseldorf, Germany	Researcher	
Norman	Kluthausen			Department of Psychiatry (Psychiatric University Hospital LVR/HHU Düsseldorf), University of Düsseldorf, Germany	Düsseldorf, Germany	Researcher	
Gerald	Antoch			Department of Psychiatry (Psychiatric University Hospital LVR/HHU Düsseldorf), University of Düsseldorf, Germany	Düsseldorf, Germany	Researcher	
Julian	Caspers			Department of Psychiatry (Psychiatric University Hospital LVR/HHU Düsseldorf), University of Düsseldorf, Germany	Düsseldorf, Germany	Researcher	

Supplemental Online Content: Nonauthor Collaborators
*First name, last name, and suffix (if applicable) are required and will appear in PubMed.

***First Name and Middle Initial(s)**	***Last Name**	***Suffix (eg, Jr, III)**	Academic Degrees	Institution	Location (city, state/province, country)	Role or Contribution, eg, chair, principal investigator	Group (if more than 1 Group listed in the byline) and/or Subgroup (eg, Steering Committee)
Hans-Jörg	Wittsack			Department of Psychiatry (Psychiatric University Hospital LVR/HHU Düsseldorf), University of Düsseldorf, Germany	Düsseldorf, Germany	Researcher	
Grazia	Caforio			Department of Basic Medical Science, Neuroscience and Sense Organs - University of Bari Aldo Moro	Bari, Italy	Researcher	
Leonardo	Fazio			Department of Basic Medical Science, Neuroscience and Sense Organs - University of Bari Aldo Moro	Bari, Italy	Researcher	
Tiziana	Quarto			Department of Basic Medical Science, Neuroscience and Sense Organs - University of Bari Aldo Moro	Bari, Italy	Researcher	
Barbara	Gelao			Department of Basic Medical Science, Neuroscience and Sense Organs - University of Bari Aldo Moro	Bari, Italy	Researcher	
Raffaella	Romano			Department of Basic Medical Science, Neuroscience and Sense Organs - University of Bari Aldo Moro	Bari, Italy	Researcher	
Ileana	Andriola			Department of Basic Medical Science, Neuroscience and Sense Organs - University of Bari Aldo Moro	Bari, Italy	Researcher	

Supplemental Online Content: Nonauthor Collaborators
*First name, last name, and suffix (if applicable) are required and will appear in PubMed.

***First Name and Middle Initial(s)**	***Last Name**	***Suffix (eg, Jr, III)**	Academic Degrees	Institution	Location (city, state/province, country)	Role or Contribution, eg, chair, principal investigator	Group (if more than 1 Group listed in the byline) and/or Subgroup (eg, Steering Committee)
Andrea	Falsetti			Department of Basic Medical Science, Neuroscience and Sense Organs - University of Bari Aldo Moro	Bari, Italy	Researcher	
Marina	Barone			Department of Basic Medical Science, Neuroscience and Sense Organs - University of Bari Aldo Moro	Bari, Italy	Researcher	
Roberta	Passiatore			Department of Basic Medical Science, Neuroscience and Sense Organs - University of Bari Aldo Moro	Bari, Italy	Researcher	
Marina	Sangiuliano			Department of Basic Medical Science, Neuroscience and Sense Organs - University of Bari Aldo Moro	Bari, Italy	Researcher	
Marian	Surmann			Department of Psychiatry and Psychotherapy of the University of Münster, Germany	Münster, Germany	Researcher	
Olga	Bienek			Department of Psychiatry and Psychotherapy of the University of Münster, Germany	Münster, Germany	Researcher	
Ana Beatriz	Solana			General Electric Global Research Inc., USA	Munich, Germany	Researcher	
Manuela	Abraham			General Electric Global Research Inc., USA	Munich, Germany	Researcher	
Timo	Schirmer			General Electric Global Research Inc., USA	Munich, Germany	Researcher	
Carlo	Altamura			University of Milan, Italy	Milan, Italy	Researcher	
Marika	Belleri			University of Milan, Italy	Milan, Italy	Researcher	
Francesca	Bottinelli			University of Milan, Italy	Milan, Italy	Researcher	

Supplemental Online Content: Nonauthor Collaborators
*First name, last name, and suffix (if applicable) are required and will appear in PubMed.

***First Name and Middle Initial(s)**	***Last Name**	***Suffix (eg, Jr, III)**	Academic Degrees	Institution	Location (city, state/province, country)	Role or Contribution, eg, chair, principal investigator	Group (if more than 1 Group listed in the byline) and/or Subgroup (eg, Steering Committee)
Adele	Ferro			University of Milan, Italy	Milan, Italy	Researcher	
Marta	Re			University of Milan, Italy	Milan, Italy	Researcher	
Emiliano	Monzani			University of Milan, Italy	Milan, Italy	Researcher	
Maurizio	Sberna			University of Milan, Italy	Milan, Italy	Researcher	
Armando	D’Agostino			University of Milan, Italy	Milan, Italy	Researcher	
Lorenzo	Del Fabro			University of Milan, Italy	Milan, Italy	Researcher	
Giampaolo	Perna			University of Milan, Italy	Milan, Italy	Researcher	
Maria	Nobile			University of Milan, Italy	Milan, Italy	Researcher	
Alessandra	Alciati			University of Milan, Italy	Milan, Italy	Researcher	
Matteo	Balestrieri			University of Udine, Italy	Udine, Italy	Researcher	
Carolina	Bonivento			University of Udine, Italy	Udine, Italy	Researcher	
Giuseppe	Cabras			University of Udine, Italy	Udine, Italy	Researcher	
Franco	Fabbro			University of Udine, Italy	Udine, Italy	Researcher	
Marco	Garzitto			University of Udine, Italy	Udine, Italy	Researcher	
Sara	Piccin			University of Udine, Italy	Udine, Italy	Researcher	
