# Supplementary material for: Multivariate Brain-Blood Signatures in Early-Stage Depression and Psychosis
Source: JAMA Psychiatry. 2025 Dec 17;83(2):172–84. doi: 10.1001/jamapsychiatry.2025.3803 (PMC12712837; doi:10.1001/jamapsychiatry.2025.3803)
Supplement: Supplement 4. — Data sharing statement [file jamapsychiatry-e253803-s004.pdf]

Data Sharing Statement

Popovic. Multivariate Brain-Blood Signatures in Early-Stage Depression and Psychosis. *JAMA Psychiatry*. Published December 17, 2025. doi:10.1001/jamapsychiatry.2025.3803

Data
Data available: Yes
Data types: Deidentified participant data, Other (please specify)
Additional Information: All data is available through request from the PRONIA Consortium (Principal Investigator: Nikolaos Koutsouleris)
How to access data: Nikolaos.Koutsouleris@med.uni-muenchen.de
When available: With publication

Supporting Documents
Document types: None

Additional Information
Who can access the data: Anyone requesting the data, submitting a proposal and receiving approval of this proposal through the PRONIA Consortium
Types of analyses: For any purpose.
Mechanisms of data availability: After approval of a proposal
